# Maternal and infant gut microbiome

**DOI:** 10.1002/imt2.70151

**Published:** 2026-08-04

**Authors:** Huidi Wang, Felicia Tin, Hui Chen, Fuxin Jiao, Min Wu, Shanshan Sun, Lisha Lin, Die Li, Huimin Zheng, Zhenxin Niu, Mengyao Lan, Bahtiyar Yilmaz, Johan G. Eriksson, Mengjia Wang, Andrew Macpherson, Jose C. Clemente, Jia Xu, Ri‐hua Xie, Xiaojiao Zheng, Tao Zhou, Jinfeng Wang, Wei Shen, Bing Huang, Xia Chen, Hai Li, Yan He

**Affiliations:** ^1^ Microbiome Medicine Center, Department of Laboratory Medicine, Zhujiang Hospital Southern Medical University Guangzhou China; ^2^ Institute for Human Development and Potential, Agency for Science Technology and Research (A*STAR) Singapore Singapore; ^3^ School of Nursing Southern Medical University Guangzhou China; ^4^ Center for Translational Medicine, Shanghai Key Laboratory of Diabetes Mellitus Shanghai Sixth People's Hospital Affiliated to Shanghai Jiao Tong University School of Medicine Shanghai China; ^5^ Faculty of Life and Health Sciences Shenzhen University of Advanced Technology Shenzhen China; ^6^ College of Food Science & Nutritional Engineering China Agricultural University Beijing China; ^7^ Department of Pediatrics, Nanfang Hospital Southern Medical University Guangzhou China; ^8^ Guangdong Provincial Key Laboratory of Gastroenterology, Department of Gastroenterology, Nanfang Hospital Southern Medical University Guangzhou China; ^9^ Ningbo Key Laboratory of Human Microbiome and Precision Medicine, Central Laboratory of the Medical Research Center The First Affiliated Hospital of Ningbo University Ningbo China; ^10^ UNSW Microbiome Research Centre St George and Sutherland Clinical Campuses, UNSW Sydney Australia; ^11^ State Key Laboratory of Immune Response and Immunotherapy, Department of Pediatrics, The First Affiliated Hospital of USTC, Division of Life Sciences and Medicine University of Science and Technology of China Hefei China; ^12^ Department of Visceral Surgery and Medicine, Bern University Hospital University of Bern Bern Switzerland; ^13^ Human Potential Translational Research Programme, Yong Loo Lin School of Medicine National University of Singapore Singapore Singapore; ^14^ Department of Obstetrics and Gynaecology, Yong Loo Lin School of Medicine National University of Singapore Singapore Singapore; ^15^ Department of General Practice and Primary Health Care University of Helsinki Helsinki Finland; ^16^ Department of Genetics and Genomic Science Icahn School of Medicine at Mount Sinai New York New York USA; ^17^ The Affiliated Foshan Women and Children Hospital, School of Nursing Guangdong Medical University Foshan China; ^18^ State Key Laboratory of Multi‐organ Injury Prevention and Treatment Southern Medical University Guangzhou China; ^19^ Guangdong Provincial Clinical Research Center for Laboratory Medicine Guangzhou China; ^20^ Key Laboratory of Mental Health of the Ministry of Education Guangzhou China; ^21^ Guangdong‐Hong Kong‐Macao Greater Bay Area Center for Brain Science and Brain‐Inspired Intelligence Guangzhou China; ^22^ Guangdong Basic Research Center of Excellence for Integrated Traditional and Western Medicine for Qingzhi Diseases Guangzhou China

**Keywords:** critical windows, development, dysbiosis, early‐life, infant, maternal, microbiome

## Abstract

Early‐life gut microbiome assembly is a pivotal determinant of lifelong health; however, the integrated frameworks governing this process across developmental milestones remain insufficiently defined. This review establishes a multidimensional framework by delineating the crosstalk between the gut microbiome and the host throughout the preconception, prenatal, postpartum, and early childhood stages. We first highlight the emerging paradigm of biparental microbial contributions during the preconception period, detailing how paternal and maternal niches jointly prime offspring development. Moving into pregnancy, we examine the maternal reservoir, integrating the role of gut microbiota‐derived metabolites across multiple trimesters in prenatal priming and vertical transmission. For the postpartum period, we discuss the development of the multikingdom gut microbiome and address the impacts of delivery modes and clinical interventions. Here, we articulate a critical knowledge gap: the discrepancy between taxonomic “catch‐up” and true functional restoration, particularly in vulnerable cohorts such as preterm infants. Furthermore, we propose a “developmental synchronization” model within the maternal–infant–microbiome continuum. This model posits that early‐life “windows of opportunity” are defined by the obligate temporal coupling of host physiological maturation with stage‐specific microbial metabolic signals. From a translational perspective, we discuss how this framework informs the development of precision interventions, such as stage‐specific probiotics, prebiotics, or metabolic modulators. These therapies aim to restore not only the microbial composition but also the synchronized functional dialog between the microbiome and host development. By mapping the “microbiota–metabolite–host target–physiological phenotype” network, we provide a systematic roadmap for precision‐targeted interventions during the first 1000 days of life.

## Highlights

- Maternal and early‐life microbiota shape developmental programming from preconception to childhood.
- Microbial dysbiosis during critical windows contributes to reproductive, immune, metabolic, and neurodevelopmental disorders.
- Microbiome‐based diagnostics and interventions hold promise for improving maternal and child health.

## Graphical abstract

The maternal–infant continuum involves critical windows of host–microbiota co‐adaptation. Preconception, maternal and paternal microbiomes modulate immunity and reproduction. During pregnancy, a gut–placenta axis emerges, where microbial metabolites influence fetal growth or contribute to complications like gestational diabetes mellitus (GDM), preeclampsia (PE) and preterm birth (PTB) if dysbiosis occurs. At birth, delivery mode dictates the pioneer founder microbiota; vaginal delivery provides beneficial taxa (*Bacteroides*, *Bifidobacterium*), whereas cesarean section introduces skin and environmental microbes. Postnatally, diet (breast milk vs. solid food) drives succession toward an adult‐like state. This continuum profoundly shapes immune, metabolic, and neurodevelopmental trajectories, linking early dysbiosis to later disorders like autism spectrum disorder (ASD), attention‐deficit/hyperactivity disorder (ADHD), asthma, inflammatory bowel disease (IBD), systemic lupus erythematosus (SLE), obesity, metabolic dysfunction‐associated fatty liver disease (MAFLD), and malnutrition. Translational approaches aim to restore homeostasis via diagnostics and targeted modulations.

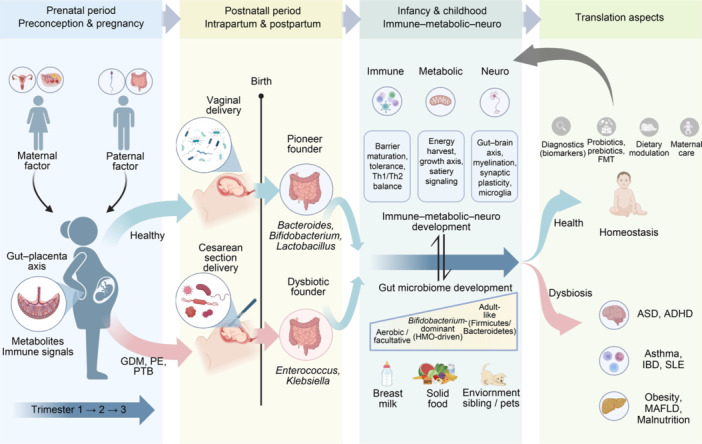

## INTRODUCTION

Maternal–infant health is increasingly recognized as an ecosystem phenomenon, in which maternal and paternal microbial communities interact with endocrine and immune circuits to shape fertility, placental function, and neonatal development. Across the reproductive timeline, microbial states are not static “snapshots” but dynamic programs that coadapt with host physiology: during pregnancy, for example, the gut microbiome remodels in a gestation‐stage‐specific manner, and growing evidence supports a gut–placenta signaling axis in which microbial metabolites (e.g., short‐chain fatty acids (SCFAs), bile acids (BAs), and indoles) are decoded by placental receptors to tune immune tolerance, vascular remodeling, and metabolic reprogramming. After birth, infant gut assembly follows a nonrandom ecological succession that is highly sensitive to delivery mode, antibiotics, and nutrition. Breast milk serves as a sustained route for both microbial transmission and a selection of substrates that steer early community structure and function. Disruption of these time‐locked microbial trajectories is associated with major perinatal and early‐life morbidities, motivating a life‐course framework that integrates gut microbiomes, metabolite‐mediated crosstalk, and critical developmental windows.

However, current microbiome research is often confined to a descriptive synthesis of taxonomic shifts, leaving several critical gaps unresolved or potentially misinterpreted. First, the field frequently overlooks the multikingdom nature of the early‐life ecosystem (including the virome, mycobiome, and resistome) and the emerging paradigm of biparental, particularly paternal microbial contributions. Second, there is a pervasive tendency in clinical studies to equate taxonomic “catch‐up” with true functional restoration. This misinterpretation is especially evident in preterm infants, where compositional recovery often masks persistent functional deficits. Another prominent example is vaginal microbiota transplantation (VMT) for cesarean section‐born infants; while VMT has been shown to partially restore the taxonomic structure of the infant microbiota, whether this intervention can sustainably achieve functional recovery or reduce the long‐term risk of diseases such as asthma and obesity remains unknown. Finally, studies predominantly characterize the “critical window” chronologically and lack a clear understanding of the obligate temporal coupling between microbial metabolic succession and host physiological milestones.

**FIGURE 1:**
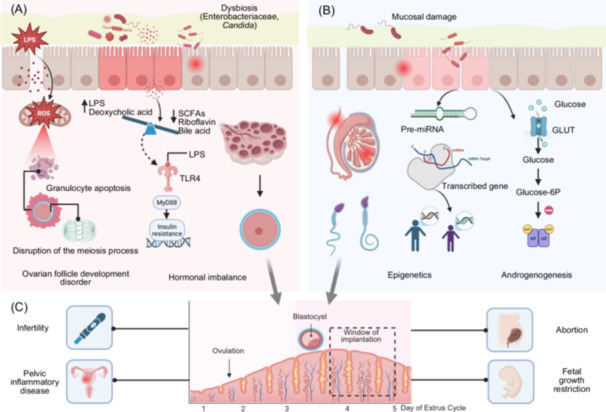
The impact of maternal and paternal gut dysbiosis on reproductive physiology and pregnancy outcomes. (A) Maternal mechanisms of reproductive impairment. Gut dysbiosis, characterized by an overgrowth of opportunistic taxa (e.g., Enterobacteriaceae and *Candida*), compromises intestinal barrier integrity and leads to the systemic translocation of lipopolysaccharide (LPS). Elevated LPS induces reactive oxygen species (ROS) accumulation, which triggers granulocyte apoptosis, disrupts the meiosis process, and culminates in ovarian follicle development disorders. Concurrently, microbial shifts alter the systemic metabolic profile, featured by elevated LPS and deoxycholic acid while depleted short‐chain fatty acids (SCFAs), riboflavin, and bile acids. Systemic LPS further activates the Toll‐like receptor 4 (TLR4) and myeloid differentiation primary response 88 (MyD88) signaling cascade, driving insulin resistance and subsequent hormonal imbalance. (B) Paternal mechanisms of reproductive impairment. Mucosal damage and dysbiosis‐driven systemic inflammation compromise the blood–testis barrier and spermatogenesis. This inflammatory microenvironment induces epigenetic modifications in sperm via altered pre‐miRNA processing and gene transcription. Furthermore, metabolic disruptions impair glucose transport (via glucose transporter (GLUT)) and glucose‐6‐phosphate (Glucose‐6P) availability, which subsequently blunts androgenesis by interfering with dihydrotestosterone (DHT) and androgen receptor (AR) signaling. (C) Clinical reproductive and developmental consequences. The convergence of compromised maternal oocyte quality/hormonal environments and epigenetically altered paternal sperm directly impacts the estrus cycle, ovulation, and the critical window of implantation for the blastocyst. Ultimately, these biparental dysbiotic factors contribute to severe clinical outcomes, including infertility, pelvic inflammatory disease, abortion, and fetal growth restriction.

To bridge these gaps, this review moves beyond descriptive observations to establish a “developmental synchronization” conceptual framework. Here, we synthesize current evidence from preconception to early childhood, explicitly mapping the “microbiota–metabolite–host target–physiological phenotype” regulatory network. By identifying areas that are poorly understood, such as the persistence of functional dysbiosis and the scarcity of intergenerational longitudinal cohorts, we aim to guide the field away from generic taxonomic profiling toward a mechanistic understanding of early‐life dysbiosis and the development of precision‐targeted, function‐restoring interventions (Figure 1).

## PRENATAL PERIOD

### Preconception

In women of reproductive age, the gut and vaginal microbiota function as coupled ecosystems that modulate systemic immunity and endocrine profiles through mechanisms such as the “estrobolome,” which regulates circulating steroid hormones. In parallel, paternal fertility is influenced by both semen‐resident microbes and gut‐derived systemic inflammation, which can compromise sperm quality and function through oxidative stress and disruption of the blood–testis barrier (Figure 1).

### Maternal factors

#### Gut and vaginal microbiota in reproductive‐age women: Composition and determinants

In women of reproductive age, the gut and vaginal microbiota are mature yet highly plastic ecosystems, best understood as an integrated phenotype shaped by long‐term determinants (diet, body composition, and hormones) and short‐term perturbations (cycle phase and sexual activity) [1, 2]. While the gut community settles into a relatively stable configuration post‐adolescence, the notion of a “standard” adult microbiota remains elusive due to persistent inter‐individual variation driven by sex, diet, and body mass index (BMI) [1, 2, 3]. This heterogeneity underscores a key limitation in the field: identifying universal compositional “norms” is less clinically useful than understanding the functional resilience of these communities in the face of disruption. Dominance at the phylum level generally rests with Firmicutes and Bacteroidetes. Sex‐specific signatures, such as lower Bacteroidetes abundance in women, further link microbial ecology to gonadal steroid activity [2, 3, 4, 5, 6].

The interplay of ovarian steroids adds a crucial temporal dimension that is often overlooked in cross‐sectional studies. Experimental data indicate that estrogens and progesterone sculpt the gut ecosystem, yet human sampling frequently fails to adequately control for the menstrual phase, potentially blurring our view of true baseline variation [6, 7, 8, 9]. In contrast, the vaginal microbiota is defined by its relative simplicity and functional focus: *Lactobacillus* predominance drives lactic acid production and maintains a low pH, which is a cornerstone of reproductive tract defense [10]. Dysbiosis, most notably bacterial vaginosis (BV), represents a well‐characterized collapse of this acidified state. An overgrowth of anaerobes such as *Gardnerella* and *Atopobium* serves as a clear microbial signature of risk [11, 12]. While cycle‐driven instability is evident during menses, which is marked by increased diversity and *Lactobacillus* decline, the clinical significance of these transient shifts versus persistent community disruption requires a more granular distinction [13, 14, 15, 16].

Crucially, these two niches are not isolated. There is compelling evidence for ecological coupling wherein the rectum serves as a reservoir for vaginal *Lactobacillus* strains, suggesting that gut health directly underpins vaginal homeostasis [17]. Furthermore, the gut's role in estrogen metabolism creates a systemic axis through which diet and metabolic status might indirectly modulate the vaginal environment [18]. This interconnected view shifts the preconception focus from isolated bacterial counts to the functional dialog between the gut's metabolic output and the vaginal ecosystem's stability.

#### How gut and vaginal microbiota modulate estrogen and progesterone: Endocrine mechanisms

The interplay between sex steroids and mucosal microbial ecosystems represents a bidirectional axis that is central to reproductive‐age physiology. Host‐derived estrogens and progesterone reshape epithelial architecture and immune tone in both the gut and vaginal niches. Concurrently, microbial enzymatic repertoires reciprocally modulate steroid bioavailability, thereby influencing endocrine signaling throughout the preconception period. This is not merely a unidirectional effect of hormones on bacteria; rather, the microbiota actively participates in calibrating the host's hormonal milieu. This recognition reframes the “baseline” endocrine status as a product of both ovarian output and microbial metabolism.

A principal mechanism through which the gut microbiota influences estrogen exposure is the deconjugation of excreted estrogens via bacterial β‐glucuronidase activity, a functional capacity collectively termed the “estrobolome” [19, 20, 21]. By cleaving glucuronic acid moieties in the intestinal lumen, this enzymatic activity liberates estrogens for reabsorption into the systemic circulation rather than fecal elimination. Taxa such as *Bifidobacterium*, *Clostridium*, and *Lactobacillus*, alongside species including *Bacteroides thetaiotaomicron* and *Clostridium perfringens*, contribute to this pool of β‐glucuronidase activity [20, 22]. This functional capacity carries significant clinical implications: inter‐individual variation in estrobolome composition may translate into differential estrogenic tone, with potential consequences for menstrual cyclicity, ovulation, and early pregnancy support. Moreover, preferential deconjugation of specific estrogens (e.g., estradiol vs. estrone) suggests that the microbiota can sculpt not only the total estrogen burden but also the qualitative profile of circulating steroids [20, 23].

Beyond estrogens, microbial contributions to progestin and androgen metabolism are increasingly recognized. While direct modulation of circulating progesterone levels by gut microbes remains less established, compelling evidence demonstrates the microbial generation of neuroactive progestins from glucocorticoid precursors. Notably, *Eggerthella lenta* and *Gordonibacter pamelaeae* produce allopregnanolone via a 21‐dehydroxylation pathway [24, 25]. This finding holds substantial conceptual significance for preconception endocrinology: it identifies a specific microbial biosynthetic route to a steroid that modulates stress responsivity and luteal‐phase neuroendocrine function, thereby linking gut ecology to central reproductive circuits. Furthermore, emerging work on the “gut–ovary axis” reveals that microbial metabolites can directly influence ovarian function. β‐resorcylic acid (β‐RA) from *Limosilactobacillus reuteri* attenuates chemotherapy‐induced ovarian damage by suppressing granulosa cell apoptosis, while 3,4‐dihydroxyphenylacetic acid (DHPAA) ameliorates polycystic ovary syndrome (PCOS)‐like phenotypes via bone morphogenetic protein signaling and anti‐Müllerian hormone reduction [26, 27].

The evidence increasingly positions the mucosal microbiota as a tunable endocrine modifier. The estrobolome serves as a critical control point for the enterohepatic recirculation of estrogens. Consequently, dysbiosis could potentially skew estrogen exposure in ways that contribute to PCOS or subfertility. Concurrently, discrete microbial pathways generate bioactive progestins and ovarian‐protective metabolites, extending the reach of the gut ecosystem beyond local immunity into the core of reproductive endocrine regulation. For the preconception period, this bidirectional crosstalk implies that interventions targeting gut microbial composition or function may offer adjunctive strategies to stabilize or optimize the hormonal environment required for successful conception.

### Paternal factors

#### Microbial composition of human semen

Human semen is not sterile. Older culture‐based work often concluded that semen from healthy men was bacteria‐free or only intermittently colonized, but sequencing‐based approaches have consistently revealed microbial DNA in ejaculate, including taxa that are difficult to culture and present at very low abundance [28, 29, 30]. The term semen microbiome is typically used to describe the microbial community detected in ejaculate (seminal plasma plus sperm‐associated fractions), while recognizing that semen is a composite biological fluid and that measured profiles can reflect both resident organisms and transient microbial signals [31]. Across studies of healthy men, community structure is variable, and no universally accepted “core semen microbiome” has been defined [32]. At the phylum level, Proteobacteria and Actinobacteria are frequently reported as predominant, with Bacteroidetes and Firmicutes also contributing substantially [32]. At finer taxonomic resolution, next‐generation sequencing (NGS) studies have reported diverse genera: one study described communities dominated (among others) by *Pelomonas, Bosea, Xylanimicrobium*, and *Phyllobacterium*, and species such as *Corynebacterium simulans* [33]. Another sequencing study identified approximately 21 genera in semen, including *Ralstonia, Corynebacterium*, and *Lactobacillus* [29]. Between‐person variability is a consistent observation; rather than converging on a single stable profile, the semen microbiota often appears individualized and shaped by both host and environmental contexts [29, 34].

Even within ostensibly healthy cohorts, semen microbiota composition varies with individual‐level factors. Environmental exposures, personal hygiene habits, and age have been explicitly linked to differences in semen microbiome composition [34]. Sexual behavior adds another axis: the exchange of microorganisms between sexual partners has been highlighted as a relevant contributor to semen microbial states and as a reason to consider the semen microbiota in the broader context of couple‐level genital microbiology [29, 35]. This distinction matters for composition‐focused interpretation, as taxa detected in semen may reflect recent sexual exchange and transient carriage rather than stable colonization of the male tract.

A central challenge in interpreting semen microbiome composition is that ejaculate is a mixture of secretions from multiple anatomical sites, meaning that detected taxa can plausibly arise from several upstream niches. Accessory glands provide most of the seminal fluid. Specifically, the prostate, seminal vesicles, and bulbourethral glands contribute the bulk of the ejaculate volume, with approximately a quarter of the ejaculate originating from the prostatic epithelium [36]. Accordingly, microbial DNA detected in semen may reflect contributions from these glands as well as from the urethra and urinary tract. At the same time, data from upper‐tract sampling underscore how careful one must be in assigning provenance. Testicular microbiota studies have produced mixed conclusions, and rigorous negative‐control approaches can substantially alter whether a “testicular microbiota” signal remains after decontamination [37, 38]. In low‐biomass testicular sperm samples, contamination may account for 50%–70% of detected bacterial reads [38]. More broadly, reproductive‐tract microbiome studies require stringent negative controls and careful interpretation of low‐biomass signals [39, 40]. Furthermore, because semen represents a low‐biomass environment, stringent controls, thoughtful sampling design, and cautious interpretation of “core taxa” are prerequisites for drawing reproducible conclusions.

#### Microbial influences on sperm quality and function

Multiple mechanistic layers plausibly connect gut dysbiosis to impaired sperm function. The gut microbiota can release soluble spermatotoxic factors, including lipopolysaccharide (LPS) and hemolysins, as well as other secreted effectors that impair sperm physiology [41, 42]. Bacteria‐induced inflammatory responses can drive reactive oxygen species (ROS) generation, leading to lipid peroxidation of the sperm membrane (which is rich in polyunsaturated fatty acids), mitochondrial dysfunction, protein damage, and DNA fragmentation [43, 44]. These cellular injuries manifest clinically as reduced motility and viability, higher DNA fragmentation rates, and compromised fertilization potential [43, 44].

Clinically, seminal microbiota differences have been reported between infertile men with and without leukocytospermia. Leukocytospermia was associated with a reduction in *Lactobacillus* species and an increase in *Streptococcus* species in one comparison [45]. Similarly, in the context of chronic prostatitis, a reduction in seminal *Lactobacillus* and an enrichment of Proteobacteria have been reported, consistent with an inflammatory ecology that can impair semen quality [46].

While the semen microbiota can act locally, systemic microbial signals (particularly gut‐derived inflammatory triggers) may prime testicular and epididymal dysfunction. LPS‐driven inflammation in animal models can compromise the blood–epididymal barrier (e.g., via the downregulation of barrier‐associated defensin pathways), worsen sperm injury, and disrupt immune privilege through cytokine‐mediated tight junction impairment in the blood–testis and blood–epididymis barriers [47, 48]. In parallel, endotoxin‐driven immune recruitment can increase ROS production within the reproductive tract, while oxidative stress‐mediated lipid peroxidation and DNA damage can reduce sperm motility and fertilization capacity [49, 50]. Diet‐associated dysbiosis provides a plausible upstream exposure: a high‐fat diet (HFD)‐induced gut microbial shift has been linked to impaired spermatogenesis and sperm motility in experimental work. This supports a model in which systemic inflammation and oxidative stress connect the host's metabolic state to sperm function [51].

#### Paternal and biparental microbial contributions to preconception health and offspring outcomes

The association between the seminal microbiome and male reproductive health has been widely substantiated. Specific microbial dysbiosis and abnormal bacterial loads are closely linked to sperm DNA fragmentation, elevated oxidative stress, and adverse outcomes such as recurrent pregnancy loss. Recent studies have further delineated the potential indirect pathways through which the paternal microbiome influences offspring health. First, seminal microbial community types enriched in taxa such as *Prevotella* or *Pseudomonas* have been associated with poorer semen quality and may contribute to reproductive dysfunction through inflammatory or oxidative‐stress‐related pathways [52]. Second, BV‐associated organisms carried by male partners may contribute to reinoculation and recurrence of BV, thereby perturbing *Lactobacillus*‐dominated vaginal homeostasis [53, 54]. These findings suggest that the paternal microbiome indirectly modulates reproductive outcomes by impairing sperm quality and perturbing the partner's reproductive tract ecology. However, its primary role currently appears confined to reshaping pre‐fertilization sperm function and maternal niche conditions.

Theoretically, semen serves as a paternal microbial vehicle capable of vertical transmission via initial intrauterine colonization and postpartum skin contact. This transmission could potentially synergize with the maternal microbiome to regulate embryo implantation and offspring immune development. However, such hypotheses regarding a “biparental microbiome co‐sculpting offspring health” remain largely speculative. Current investigations are constrained by technical challenges, including sequencing contamination in low‐biomass samples, the absence of prospective intergenerational cohorts, and insufficient strain‐level tracking methodologies. Consequently, definitive evidence confirming the direct colonization of the fetal or neonatal gut and skin by paternal strains is still lacking. It is therefore imperative to delineate the specific limitations within the existing research framework. Specifically, the direct colonization efficiency of vertical paternal microbial transmission remains unelucidated, and the synergistic or antagonistic effects of the biparental microbiota on offspring health have not been quantified. Future investigations should leverage large‐scale parent–offspring cohorts, high‐precision gnotobiotic animal models, and multi‐omics interaction networks to bridge this critical gap.

### Limitations and future perspectives

Despite growing evidence supporting the role of the gut and vaginal microbiota in modulating female and male fertility, several critical limitations remain. First, most current studies are observational and cross‐sectional, limiting causal inference regarding the microbiota–fertility relationship. The dynamic nature of microbial communities across the menstrual cycle, pregnancy, and life stages necessitates longitudinal designs with repeated sampling. Such designs are essential to capture temporal trajectories and identify stable versus transient microbial signatures. Second, the field suffers from substantial heterogeneity in study design, sequencing platforms, bioinformatic pipelines, and taxonomic resolution. This inconsistency hampers cross‐study comparability and the identification of reproducible microbial biomarkers. Third, while mechanistic insights have been gained from animal models, translation to humans remains challenging. This difficulty arises primarily from interspecies differences in microbiota composition, host physiology, and reproductive endocrinology. Fourth, the majority of research has focused on the female microbiota. Conversely, male contributions, particularly the semen microbiome and its systemic interactions, remain underexplored. Finally, confounding factors such as diet, hormonal status, sexual activity, and medication use are often inadequately controlled. This oversight introduces bias and limits interpretability.

Future research should prioritize well‐powered, multi‐omics longitudinal studies that integrate metagenomics, metabolomics, and host transcriptomics to unravel causal mechanisms. Intervention studies, including those utilizing probiotic, prebiotic, or dietary modulation, are urgently needed. These trials will establish whether manipulating the microbiota can improve fertility outcomes in a clinical setting. The concept of the “estrobolome” and its role in endocrine regulation warrants deeper mechanistic exploration. Additionally, the paternal microbiota and its influence on sperm quality, embryo development, and offspring health should be systematically investigated. Standardization of protocols and reporting guidelines will be essential to advance the field toward clinical translation. Ultimately, a couple‐based microbial perspective may offer a more integrated and personalized approach to fertility assessment and treatment.

### Pregnancy

**FIGURE 2:**
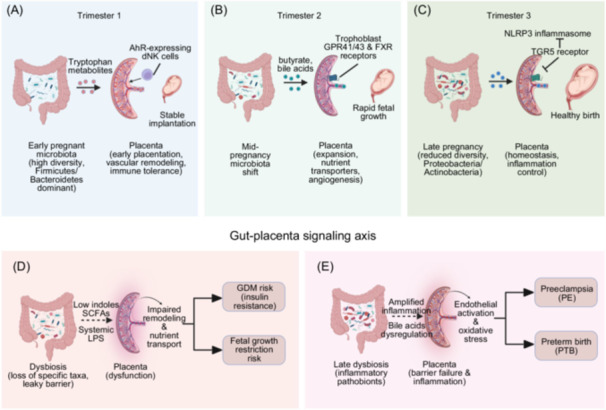
The spatiotemporal evolution of the gut–placenta signaling axis during normal pregnancy and dysbiosis‐driven complications. (A) Trimester 1. The early‐pregnancy maternal gut microbiota exhibits high diversity and is dominated by Firmicutes and Bacteroidetes. These microbes produce tryptophan metabolites (e.g., indoles) that activate the aryl hydrocarbon receptor (AhR) on decidual natural killer (dNK) cells, facilitating early placentation, essential vascular remodeling, selective immune tolerance, and stable embryo implantation. (B) Trimester 2. As pregnancy progresses, a mid‐gestational microbiota shift alters the metabolic pool, enriching short‐chain fatty acids (e.g., butyrate) and bile acids. These metabolites activate G‐protein‐coupled receptors (GPR41/43) and farnesoid X receptors (FXR) on placental trophoblasts, driving placental expansion, angiogenesis, and trans‐barrier nutrient transport to support rapid fetal growth. (C) Trimester 3. In late pregnancy, microbial diversity naturally declines alongside a physiological enrichment of Proteobacteria and Actinobacteria. Specific microbial signals interact with the Takeda G protein‐coupled receptor‐5 (TGR5) and inhibit the NOD‐like receptor family pyrin domain containing 3 (NLRP3) inflammasome, buffering metabolic load, controlling late‐stage inflammation, and maintaining placental homeostasis for a healthy birth. (D) Early/Mid‐pregnancy dysbiosis. A loss of beneficial taxa and intestinal barrier integrity (leaky gut) leads to diminished indoles and SCFAs, coupled with elevated systemic lipopolysaccharide (LPS). This disruption impairs placental remodeling and nutrient transport, driving insulin resistance and increasing the risk of gestational diabetes mellitus (GDM) and fetal growth restriction. (E) Late‐pregnancy dysbiosis. The proliferation of inflammatory pathobionts precipitates amplified systemic inflammation and bile acid dysregulation. At the maternal–fetal interface, this causes placental barrier failure, profound endothelial activation, and oxidative stress, culminating in severe clinical complications such as preeclampsia (PE) and preterm birth (PTB).

Compared to the preconception stage, the maternal gut microbiota undergoes dynamic remodeling during pregnancy, establishing a spatiotemporally specific host–microbiota co‐adaptation. Microbial metabolites act through the placental signaling hub to stage‐dependently regulate immune tolerance, vascular remodeling, and metabolic adaptation. Deviation from this adaptive range may increase the risk of gestational diabetes mellitus (GDM) and preeclampsia (PE). By providing substrates for microbial fermentation and shaping microbial metabolic outputs, the maternal diet may influence both placental signaling and infant microbial assembly. This section systematically reviews the evolution of the gut microbiota during pregnancy, its influencing factors, placental signaling mechanisms, and pregnancy complications, and discusses clinical translation prospects (Figure 2).

### Maternal gut microbiota during pregnancy and influencing factors

Pregnancy is a state of host–microbiota co‐adaptation. In the first trimester, the maternal gut microbiome resembles that of non‐pregnant women, dominated by *Bacteroidetes* and Firmicutes (e.g., Clostridiales) [55]. By the third trimester, α‐diversity decreases and β‐diversity increases, while Proteobacteria and Actinobacteria rise [55]. Specifically, Clostridiales decrease, and Enterobacteriaceae expand [55]. In non‐pregnant populations, such changes are often associated with metabolic syndrome, but they also occur in healthy third‐trimester pregnancies, suggesting a normal adaptive role. Yet, late gestation is also the peak period for GDM and PE [56, 57], and unfavorable microbiota shifts may contribute to complications. Germ‐free (GF) mice transplanted with third‐trimester human fecal microbiota developed features of metabolic syndrome (weight gain, insulin resistance, and low‐grade inflammation), whereas first‐trimester donors did not [55]. This experiment strongly suggests that the late‐pregnancy gut microbiota is sufficient to actively drive metabolic changes, rather than being a bystander.

Hormones, especially progesterone, drive gut microbiota changes, expanding *Bifidobacterium* in the third trimester [58]. This bifidobacterial bloom may be adaptive, as *Bifidobacterium* improves metabolic status and reduces inflammatory cytokines [59]. Higher *Bifidobacterium* levels predict better pregnancy outcomes, while lower levels increase preterm birth risk [60]. Clinical observations indicate that progesterone therapy reduces preterm birth recurrence by one‐third. This supports a progesterone–microbiota–outcome interplay, even though the microbiota was not directly assessed in those trials [61].

Among host and environmental factors that shape the maternal gut microbiome, maternal diet deserves particular emphasis as a modifiable driver that directly shapes microbial substrate availability and metabolic outputs. HFD increases the Firmicutes/Bacteroidetes ratio, enriches detrimental genera such as *Romboutsia* and *Dubosiella*, and suppresses SCFA‐producing Lachnospiraceae [62, 63]. The Mediterranean diet increases SCFA‐producing groups (Lachnospiraceae/Ruminococcaceae) and decreases *Campylobacter* [64]. Fermentable dietary fibers (inulin, resistant starch, and fructooligosaccharides) stably increase SCFA levels and improve insulin sensitivity. In GDM mice, inulin fructans reduce insulin resistance and dyslipidemia, enrich *Bifidobacterium* and *Akkermansia*, and reverse *Dubosiella* expansion [65]. In an in vitro fermentation model of GDM, fructooligosaccharides enriched *Bifidobacterium*, *Lactobacillus*, and *Roseburia*, reduced *Escherichia/Shigella*, and increased acetate production [66]. Diets enriched in fiber, vegetable protein, and fish oil have been associated with improved maternal mucosal immunity, lipid metabolism, and SCFA‐related microbial function. In contrast, HFD and maternal obesity can independently alter maternal immunity, the gut microbiota, and fetal or placental growth in mouse models [67, 68]. In human cohorts, maternal dietary patterns during pregnancy are associated with distinct maternal gut microbiota clusters, neonatal microbiota profiles, and infant growth trajectories during the first 18 months. This suggests that the maternal diet exerts a transgenerational influence [69]. Additionally, a study found that high maternal fat intake is linked to a depletion of *Bacteroides* in offspring [70]. Conversely, maternal vegetable and fruit consumption is associated with a low abundance of *Sutterella*, *Prevotella*, and *Lachnoclostridium* alongside a higher overall diversity in the infant gut [71]. Specific dietary components such as Neu5Gc (from red meat) can be incorporated into human milk oligosaccharides (HMOs), altering bacterial selection [72]. Maternal fish oil supplementation reduces defensive inflammation in breast milk, shaping an infant bacteriome with anti‐inflammatory potential [73], although excessive doses may harbor risks [74].

Despite these converging patterns, substantial inter‐individual heterogeneity exists: geography, diet, antibiotics, and pre‐pregnancy BMI contribute to divergent microbiota trajectories [75]. This reflects the staged nature of pregnancy: it requires sequential immune tolerance, placentation, and metabolic reprogramming [76, 77]. This explains why a static “key microbiota” has yielded inconsistent results across cohorts [76, 78]. Longitudinal studies find modest within‐individual taxonomic shifts, while host factors (BMI, diet) dominate between‐individual differences [79, 80]. Importantly, a relatively stable taxonomy can mask functional reprogramming. Metatranscriptomic data indicate late‐pregnancy shifts in expressed pathways consistent with altered energy processing (e.g., increased carbohydrate transport and fermentation signatures) [81]. Beyond bacteria, the gut mycobiome is even more dynamic and sensitive to maternal metabolic status [82]. Parity has been proposed as an “ecological memory” that shapes microbiota remodeling, a concept supported by controlled animal models [80]. Thus, a functional, time‐weighted view is warranted. Pregnancy microbiome changes may be adaptive, but deviations can track disease susceptibility [83, 84].

The maternal gut microbiota shifts described above do not directly affect the fetus. Instead, their metabolic signals are relayed through the maternal–fetal interface (MFI), i.e., the placenta, which decodes microbial cues into stage‐specific programs of fetal development and long‐term offspring health.

### From first to third trimester: The placenta as a microbial signal hub

#### First trimester (implantation and early placentation): Immune tolerance and vascular remodeling

In early pregnancy, the MFI must simultaneously establish selective immune tolerance and initiate spiral artery remodeling to secure early placental perfusion [85, 86]. The MFI receptor landscape is “primed” to decode low‐intensity immune tolerance signals. Uterine natural killer (uNK) cells, regulatory T cells (Tregs), and trophoblasts highly express the aryl hydrocarbon receptor (AhR). This expression enables the recognition of microbiota‐derived tryptophan metabolites (indole derivatives) that drive tolerogenic programs [85, 87]. Concurrently, activation of the indoleamine 2,3‐dioxygenase (IDO) pathway limits effector T‐cell proliferation, bridging microbial signals to immune quiescence [88, 89]. Moderate AhR signaling redirects uNK cells from cytotoxicity toward angiogenic phenotypes. This shift promotes the secretion of factors such as vascular endothelial growth factor (VEGF) and facilitates spiral artery transformation [90, 91, 92]. At the same time, local immune‐regulatory mechanisms at the maternal–fetal interface help balance trophoblast invasion, immune tolerance, and protection from excessive inflammatory injury [93].

For AhR signaling, inadequate activation undermines tolerance, whereas excessive activation inhibits vascular remodeling. However, this dose–effect relationship remains unquantified, limiting the clinical translation of AhR‐targeted interventions. When maternal gut microbial function is misaligned at this stage (e.g., due to reduced tryptophan metabolites, insufficient AhR ligands, or persistent low‐grade inflammatory signaling), the consequences can be severe. Even in the absence of infection, these disruptions lead to tolerance destabilization, impaired trophoblast invasion, and defective vascular remodeling [93]. More importantly, early placental set points are difficult to reset later in gestation. The first‐trimester intervention window is much narrower than those in mid‐ or late pregnancy. Yet, almost all clinical trials start from the second trimester, which may explain their limited efficacy [93].

#### Second trimester (placental expansion): Metabolic amplification and trans‐barrier nutrient transfer

As pregnancy enters mid‐gestation, fetal demands accelerate. This acceleration shifts the MFI toward expanding vascular capacity and nutrient transport while maintaining low inflammation [94]. Maternal gut‐derived metabolites may contribute to maternal–fetal interface adaptation and fetal growth regulation through gut–placental signaling pathways [95]. Butyrate and other SCFAs activate GPR signaling, promote angiogenesis, and upregulate glucose/lipid transport [96, 97, 98]. Importantly, MFI immune regulation is also recalibrated. Complement regulators such as CD55 peak in mid‐pregnancy, buffering inflammation during high cellular turnover and nutrient flux [94]. This coupling of metabolic amplification with immune stability stabilizes fetal growth during rapid organ enlargement.

However, overactive placental nutrient transport may lead to macrosomia. The appropriateness of microbial signals matters more than their absolute abundance, and no safe upper limit for SCFAs has been defined. If microbial metabolic signaling is blunted, the earliest consequences are expected to be suboptimal vascularization and nutrient transport, sometimes even occurring without strong inflammatory signatures [94, 95, 99, 100]. Because mid‐gestation sets fetal growth velocity, these deficits may program birth weight and later metabolic health, even if the pregnancy continues to term [94, 95, 99, 100]. This implies that focusing solely on delivery outcomes may underestimate the intergenerational impact of mid‐gestational dysbiosis.

#### Third trimester (homeostasis and risk management): Metabolic buffering, barrier integrity, and inflammation control

In late gestation, the system faces peak metabolic load and increasing inflammatory readiness as parturition approaches. Developmental priorities shift from organ building to functional maturation, energy storage, and resilience to inflammatory exposures. Accordingly, the microbiota's value lies more in sustaining metabolic buffering, barrier support, and controlled inflammation. BA receptors, such as the farnesoid X receptor (FXR) and Takeda G protein‐coupled receptor‐5 (TGR5), may help maintain placental metabolic stability [93, 95]. Furthermore, AhR remains expressed in trophoblasts, and based on its known functions in other epithelial systems (barrier maintenance, antioxidant defense, and metabolic homeostasis), it may undergo a functional switch in late pregnancy [100, 101, 102]. Stage‐dependent receptor expression and ligand fluctuations suggest that the same receptor might respond differently depending on the gestational week; however, direct evidence remains lacking [99, 100, 102, 103, 104, 105].

BA signaling can limit NOD‐like receptor family pyrin domain containing 3 (NLRP3) inflammasome activation and pro‐inflammatory immune responses at the maternal–fetal interface, helping maintain a less inflammatory pregnancy environment [106]. Moderate inflammation, conversely, supports the terminal maturation of fetal nervous, lung, and immune systems [107, 108]. When homeostatic microbial functions deteriorate, the MFI exhibits lowered inflammatory thresholds, which increases vulnerability to preterm birth [83, 94, 109]. Late‐gestation dysbiosis‐linked immune activation more readily elevates interleukin‐17 (IL‐17). This elevation potentially compromises placental barrier function and intersects with fetal neurodevelopmental trajectories, an observation that is consistent with a prenatal “microbiota–immune–brain axis” model [85, 92, 93]. Such homeostatic imbalance in late pregnancy may disproportionately affect perinatal health and long‐term offspring risk [93, 110, 111].

Translating this perspective into practice, microbiota‐targeted strategies should be mild, stage‐weighted “signal calibration” rather than aggressive remodeling [112, 113]. For instance, interventions may include first‐trimester tryptophan‐related immunomodulation support [85, 114]. Fermentable fibers, including inulin‐type fructans, can increase SCFA production and may improve glucose and lipid metabolic regulation in experimental GDM contexts [115, 116, 117]. Finally, pregnancy and lactation data suggest that well‐characterized probiotics and prebiotics are generally safe, but their use should remain strain‐specific, indication‐specific, and cautious, particularly in late pregnancy [118, 119, 120, 121, 122]. A double‐blind randomized controlled trial in overweight or obese pregnant women found no significant probiotic effect on GDM prevention or major metabolic outcomes [123], reinforcing the need for individualized, evidence‐weighted interventions [124, 125, 126, 127, 128]. When calibration fails to prevent imbalance, complication‐driven gut dysbiosis pushes the MFI beyond its adaptive range. This destabilizes the interface and amplifies pathology.

### Pregnancy complications: Gestational diabetes mellitus, preeclampsia, intrahepatic cholestasis of pregnancy, and preterm birth

Pregnancy entails coordinated metabolic and immune adaptations that may be accompanied by subtle changes in gut microbial function. Complications such as gestational diabetes mellitus (GDM), preeclampsia (PE), intrahepatic cholestasis of pregnancy (ICP), and preterm birth (PTB) can be viewed as departures from this adaptive range. Within this context, microbiome–metabolite–host interactions may amplify inflammatory tone, alter nutrient handling, or perturb BA levels and vascular pathways. Although evidence varies across study designs, we focus here on convergent pathways and areas with mechanistic or predictive support.

#### Gestational diabetes mellitus

Several lines of evidence link the maternal gut microbiome to GDM, including early‐pregnancy metabolite signatures and microbial community features. A metabolome‐wide association analysis identified early‐ to mid‐pregnancy serum metabolite clusters, partly derived from microbial metabolism, that predicted subsequent GDM diagnosis [129]. Across studies, GDM is often associated with a reduced abundance of SCFA‐producing taxa and an enrichment of opportunistic organisms, though the specific taxa vary by cohort [84]. To capture this dysbiosis more consistently, network‐ or guild‐based approaches, such as XNP_Guild1, have been proposed [84].

Evidence for a causal contribution is supported by transplantation studies, where fecal microbiota from early‐pregnancy donors who later developed GDM induced insulin resistance and inflammation in GF mice [130]. Maternal–fetal metabolomic analyses further suggest that maternal metabolic alterations are transmitted to the fetus through placental exposures rather than fetal colonization [131]. SCFAs and tryptophan‐derived metabolites have been implicated as candidate mediators linking microbial metabolism to glucose homeostasis [132].

Diet–microbiome interactions may further shape GDM risk. Higher circulating linoleic acid was associated with reduced GDM risk, but this relationship was attenuated in women with a higher abundance of *Bilophila*, highlighting context‐dependent dietary effects [133].

Intervention studies support a potentially “modifiable” role of the microbiome. However, results remain inconsistent, as meta‐analyses of randomized trials suggest limited and potentially population‐, timing‐, and strain‐specific benefits of probiotics for GDM prevention or metabolic control [134, 135].

Together, the evidence supports a model in which GDM risk can be preceded by microbiome‐associated inflammatory signals. Meanwhile, fetal exposure is mediated primarily through metabolites and inflammatory factors rather than *in utero* microbial colonization.

#### Preeclampsia

PE is characterized clinically by hypertension and proteinuria and biologically by placental dysfunction and maternal endothelial activation [136]. Metagenomic profiling has identified not only taxonomic shifts but also functional enrichment in transport systems and vitamin/cofactor pathways in individuals with PE. This suggests that microbial community function may be as important as taxonomic composition [136]. Case–control studies of severe PE report distinct gut community and fecal metabolite signatures compared with controls. Furthermore, some taxa have been correlated with blood pressure and inflammatory markers [137]. Metabolite‐mediated mechanisms are increasingly emphasized, including reduced SCFA availability and the activation of trimethylamine N‐oxide (TMAO)‐related pathways linked to endothelial dysfunction and PE‐like phenotypes in animal models [90]. These microbial and metabolic changes may contribute to placental underperfusion, fetal growth restriction, and later‐life cardiovascular risk in offspring [138].

Observational data suggest that PE is associated with gut dysbiosis and increased circulating microbial or microbiota‐related metabolites, including LPS and TMAO [139]. In a rat model, PE‐associated gut dysbiosis has been linked to reduced SCFA‐producing bacteria and SCFA availability. Crucially, restoration with *Akkermansia muciniphila*, propionate, or butyrate alleviated PE‐like features. This protective effect is potentially mediated through macrophage polarization and trophoblast‐related placental remodeling [90]. In parallel, PE‐derived gut microbiota induced hypertension, proteinuria, fetal and placental growth impairment, and immune imbalance in recipient mice. This implicates intestinal barrier leakage and bacterial translocation as a mechanistic bridge between gut dysbiosis and PE‐like disease [91]. Observational studies also suggest that probiotic milk intake may be associated with reduced risk of PE in cohort analyses, although evidence from interventional trials in humans remains scarce [140]. Overall, longitudinal and well‐controlled human studies are needed to distinguish upstream microbial drivers from downstream consequences of established disease.

#### Intrahepatic cholestasis of pregnancy

ICP provides a clear link between pregnancy complication risk and BA physiology. Reported microbiome changes in ICP include the enrichment of *Escherichia*/*Shigella* and *Turicibacter* and the depletion of genera such as *Megamonas*. These shifts correlate with total BA levels and liver enzyme markers [141]. These associations are consistent with a role for the gut microbiome in modulating BA metabolism and enterohepatic circulation. However, the causal direction remains uncertain and is likely bidirectional.

#### Preterm birth and inflammation

The maternal gut microbiome has also emerged as a potential early predictor of PTB. In a large multi‐cohort study, early‐pregnancy microbiome features were associated with later PTB risk. Consequently, specific taxa were proposed as candidate biomarkers [142]. Functional hypotheses have been proposed in relation to hormonal and inflammatory regulation, but mechanistic attribution in humans remains challenging [142].

PTB also intersects with microbial signals from other compartments and with placental inflammation. Although the existence of a distinct placental microbiome remains controversial due to low biomass and contamination risk, some studies report microbial DNA signatures associated with PTB, noting overlaps with oral and vaginal taxa [143]. These findings reinforce the need to integrate across microbial niches when interpreting gut‐based predictors and to apply rigorous controls in low‐biomass studies.

### Limitations and perspectives

Maternal microbiota effects follow a time‐weighted evolution rather than a simple stage switching. Our “time‐window and functional‐demand” framework identifies stage‐specific nodes susceptible to microbial modulation. However, this is not a rigid blueprint. Time windows overlap and buffer each other, while individual variability alters their weight. Furthermore, redundant host mechanisms (e.g., endocrine, nutritional, and metabolic pathways) can partially compensate for the attenuation of microbial pathways. Thus, the microbiota acts as an important modulator, not a sole switch.

Studies of the gestational gut microbiome face several recurring limitations. Sampling protocols often misalign with key clinical time points, undermining translational value [84, 133]. Cross‐sectional or post‐diagnosis designs risk reverse causality (diet and medication reshape the microbiome) [133]. Confounders (e.g., BMI, parity, and antibiotic exposure) often explain more variance than the complication phenotype itself. Consequently, inconsistent statistical adjustment across cohorts can lead to spurious associations [79]. Furthermore, technical heterogeneity such as the limited taxonomic resolution of 16S rRNA sequencing and the sensitivity of metagenomics and metabolomics to batch effects constrains cross‐study synthesis. Additionally, most data come from high‐income populations, raising doubts about the generalizability of identified biomarkers [144].

Future research should precisely identify individualized bottlenecks that cause window disruption or signal decoding failure. This approach shifts interventions from “pursuing an idealized temporal sequence” to “correcting functional mismatches at critical nodes”. Using the maternal–fetal interface as a hub, dispersed pregnancy complications can be integrated into network‐level functional abnormalities, revealing shared mechanisms that transcend single‐disease labels. However, the prenatal perspective is only part of a continuum. The intrapartum and early postpartum windows critically mediate vertical microbial transmission and neonatal gut establishment. These processes are shaped by delivery mode, antibiotic use, breast milk, and the care environment, all of which introduce analogous methodological challenges. Therefore, fully understanding the gestational microbiota requires integrating its postpartum carryover effects.

## POSTNATAL PERIOD

### Intrapartum and early postpartum

**Figure 3:**
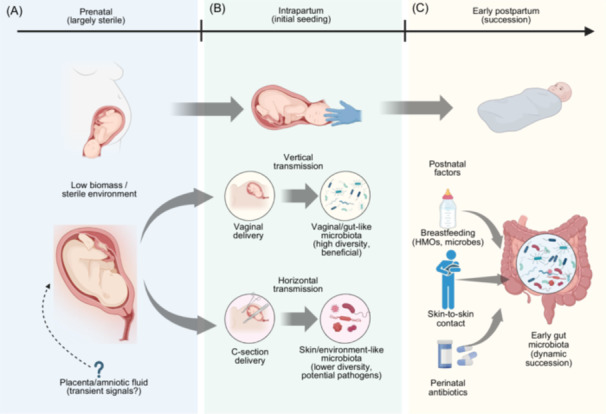
Dynamics of infant gut microbiota assembly and succession across the prenatal, intrapartum, and early postpartum periods. (A) Prenatal period. The fetus develops in a predominantly sterile, low‐biomass environment *in utero*. Current evidence indicates that there is no consistent resident placental or fetal microbiota; although transient microbial signals may cross the maternal–fetal interface via the placenta or amniotic fluid, they do not establish stable colonization prior to birth. (B) Intrapartum period (initial seeding). Delivery mode acts as a primary ecological filter that dictates the first microbial inoculum. Vaginal delivery facilitates continuous vertical transmission, exposing the neonate to maternal vaginal and gut reservoirs and resulting in a highly diverse, beneficial microbiota. In contrast, cesarean section (C‐section) delivery bypasses these primary maternal niches, driving a horizontal transmission route. This reroutes early colonization toward maternal skin and environmental sources, yielding a microbiota with lower diversity and a higher proportion of potential opportunistic pathogens. (C) Early postpartum period (succession). Following the initial intrapartum seeding, the neonatal gut microbiota undergoes dynamic ecological succession shaped by modifiable postnatal care factors. Breastfeeding provides continuous microbial input and essential prebiotics, such as human milk oligosaccharides (HMOs), to selectively reinforce beneficial communities. Concurrently, routine practices like skin‐to‐skin contact stabilize early microbial dynamics through repeated maternal exposure. Conversely, clinical interventions such as perinatal antibiotics can impose severe ecological disturbances, depleting commensal taxa and profoundly altering the trajectory of early gut microbiota maturation.

The intrapartum and immediate postpartum period represents a critical window for microbial and host development [145, 146, 147]. During this time, neonates acquire a founder gut microbiota. Neonatal colonization arises from multiple maternal sources, including the vagina, gut, skin, oral cavity, and breast milk. Furthermore, this process varies with delivery mode and perinatal exposures [148, 149, 150]. Early microbial assembly is best conceptualized as a multikingdom ecological process (involving bacteria, fungi, and viruses). This process is shaped by clinical pressures such as antibiotic use, which may also introduce antimicrobial resistance determinants. Rather than reflecting a single dominant source, colonization emerges from sequential microbial inputs followed by ecological filtering during birth and early postnatal care (Figure 3).

### The first colonizer debate

Early support for an *in utero* microbiota originated from culture‐ and sequencing‐based analyses of placental tissue, amniotic fluid, meconium, and fetal gut samples. These studies detected low‐biomass microbial signals and proposed maternal–fetal transmission routes [151, 152, 153]. However, such samples are highly vulnerable to contamination from reagents, instruments, operating environments, adjacent maternal tissues, and the so‐called “kitome” [151, 154, 155, 156, 157, 158]. Re‐analyses found that several reported taxa were biologically implausible and likely reflected reagent contamination. This underscores the need for stringent negative controls and careful decontamination procedures [151, 154, 155, 156, 157, 158].

A more balanced interpretation is that current evidence does not support a consistent resident placental or fetal microbiota. However, transient microbial signals may cross the maternal–fetal interface and influence fetal immune development without establishing stable colonization [159, 160, 161]. Pathologic exposures (e.g., intra‐amniotic infection, chorioamnionitis, membrane rupture, or inflammation) should not be misconstrued as normal colonization. Overall, the fetus most likely develops in a largely sterile environment, while stable microbial colonization begins around delivery and consolidates during the early postpartum period.

### Sources of first colonizers

#### Vagina and gut

In vaginally delivered neonates, early gut microbiota often resembles the maternal vaginal microbiota, dominated by *Lactobacillus*, *Prevotella*, or *Sneathia* species [162, 163]. However, strain‐level metagenomic analyses show that this resemblance is not equivalent to durable inheritance. Birth involves rapid exposure to microbes from multiple maternal sources, followed by strong ecological selection during the first days of life. Although strains associated with the maternal vagina and skin are readily acquired at birth, their presence in the infant gut is typically transient. In contrast, a substantial proportion of neonatal gut strains that achieve stable engraftment show high genetic similarity to strains derived from the maternal gut, consistent with the fecal–oral vertical transmission occurring during delivery and early postnatal life [148].

Across microbial kingdoms, early fungal establishment appears to involve maternal transfer and age‐dependent succession, shifting from *Candida* at birth to *Malassezia* and *Cystofilobasidium* at 6 months and later to *Trichosporon* in infancy [164, 165]. Vertical viral transmission is more selective: about 63% of the infant bacterial microbiome, but only 15% of the virome, was traceable to maternal sources [166]. Infant viromes were enriched in Myoviridae, Podoviridae, and Siphoviridae, whereas maternal viromes were enriched in Microviridae [166]. More recent longitudinal data have shown a dynamic infant virome with more active temperate phages and detectable mother–infant viral strain sharing [167]. Birth antibiotics may further disrupt vertical transmission and reshape the early resistome [168]. Maternal vaginal exposure contributes primarily to initial inoculation, whereas the maternal gut more strongly supports persistent microbial inheritance.

#### Skin and oral cavity

Maternal skin and oral microbes are readily transferred to neonates through routine postnatal contact, including skin‐to‐skin care and close caregiving. However, strain‐level evidence indicates that these contact‐based reservoirs contribute mainly to transient exposure rather than stable intestinal engraftment [148, 169].

The influence of skin‐to‐skin contact on the infant gut microbiota appears to be mediated less by direct, durable strain transfer and more by indirect effects, such as reduced stress responses and stabilization of early microbial dynamics [169]. Similarly, maternal oral taxa can be transmitted to infants through close contact, but their persistence in the infant gut is limited. Among oral–gut shared taxa, *Streptococcus salivarius* and *Rothia mucilaginosa* show the highest strain‐level concordance between oral and fecal samples, suggesting only a transient cross‐niche presence during early life [149].

Consistent with this interpretation, evidence from an adoptive mother–child and household‐based study indicates that oral microbial transmission is driven mainly by contact intensity and cohabitation rather than host genetic relatedness [170]. Skin and oral reservoirs therefore function primarily as repeated exposure sources that modulate early microbial dynamics, while contributing less to persistent gut colonization than maternal gut‐associated reservoirs.

#### Breast milk

Breast milk provides continuous microbial and metabolic input that reinforces gut colonization after birth. Milk‐ and skin‐derived bacteria can be prominent in the first month, accounting for nearly 40% of the gut microbiota in predominantly breastfed infants [171]. Beyond direct transfer, HMOs act as key prebiotic substrates that selectively promote *Bifidobacterium*‐enriched communities while supporting gut barrier function and immune modulation [172].

Unlike the transient microbial exposures associated with birth, breastfeeding provides sustained ecological input that promotes *Bifidobacterium* and *Lactobacillus* dominance, delays the expansion of adult‐associated taxa such as Clostridia, and shapes metabolic maturation [173]. Breast milk may also contribute fungi, bacteriophages, and resistance genes, although these remain less well characterized. Breastfeeding therefore acts as a continuing ecological force that consolidates early colonization into a more stable developmental trajectory.

### Factors that shape early colonizers

#### Delivery mode

Delivery mode acts as an early ecological filter by determining which maternal and environmental sources dominate the first inoculum of the infant gut. In vaginally delivered infants, the maternal gut and vaginal microbes provide the major inputs. This facilitates the vertical transmission of key commensals, particularly *Bacteroides*, *Bifidobacterium*, and *Parabacteroides*, which account for nearly 70% of the neonatal microbiota in the first weeks of life [163, 174]. Strain‐level studies indicate that maternal gut‐derived strains are more likely than strains from other sources to persist in the infant gut. Specific taxa, including *Bacteroides vulgatus* and vertically transmitted *Bifidobacterium longum* strains, can be shared between mothers and infants and may persist for months [148, 174, 175].

Cesarean section should therefore be understood not simply as the absence of vaginal exposure, but as a disruption of maternal gut‐associated strain transmission and a shift toward non‐gut maternal and environmental sources during early colonization [176, 177]. This shift is characterized by reduced transfer or delayed acquisition of key commensals, particularly *Bacteroides* and *Bifidobacterium*, and increased exposure to opportunistic taxa such as *Staphylococcus*, *Enterococcus*, and *Klebsiella* [176, 177, 178]. Although many early‐acquired strains do not persist over time [175], the resulting perturbation may prolong a vulnerable window of immune and metabolic programming. Consequently, this disruption has been linked to increased risks of asthma later in life [179].

Beyond bacteria, delivery mode also shapes the broader ecological context of colonization, including early mycobiome trajectories and patterns of viral exposure. This suggests that the birth route reorganizes the multikingdom environment in which colonization begins [165, 166, 180]. These insights have motivated strategies to partially restore maternal microbial transmission. Daily skin‐to‐skin contact may modestly support the normalization of gut community structure by increasing maternal contact and stabilizing early microbial dynamics [169]. More direct approaches, including vaginal seeding and VMT, aim to restore missing maternal vaginal exposure and have shown potential to shift infant microbiome development toward a more vaginal‐delivery‐like trajectory [181, 182]. A triple‐blind randomized controlled trial further showed that VMT was well tolerated and did not increase short‐term adverse events. Furthermore, it was associated with partial restoration of gut microbiome maturation and improved neurodevelopmental outcomes at 6 months [182]. These findings support the feasibility of VMT under strict safety protocols, while highlighting the need for larger studies with longer follow‐up.

#### Intrapartum antibiotics

Intrapartum antibiotics are essential for preventing and treating maternal and neonatal infections, particularly through penicillin‐based prophylaxis against group B *Streptococcus* (GBS), which has substantially reduced early‐onset neonatal GBS disease [183, 184]. However, their use during a critical window of microbiome assembly imposes a major ecological disturbance and can select for antimicrobial resistance [183, 185, 186]. Intrapartum antibiotic exposure is associated with measurable perturbations of maternal and infant microbial ecosystems. These perturbations include increased risks of postnatal maternal and neonatal yeast infections [187] and neonatal exposure to ampicillin‐resistant Enterobacteriaceae [188]. Furthermore, exposure alters early gut microbiota trajectories, which are characterized by reduced Actinobacteria and Bacteroidetes, together with an enrichment of β‐lactamase‐encoding resistance genes [189]. These findings indicate that antibiotic use at birth not only suppresses pathogens but also reshapes the composition and functional potential of early colonization.

The extent of disruption depends on the antibiotic spectrum, duration, and timing. Compared with prophylaxis, broader‐spectrum or longer‐duration regimens administered for maternal infections, perioperative care, or direct neonatal treatment are associated with a more pronounced and persistent microbial depletion. Specifically, these regimens deplete Bacteroidetes and Actinobacteria (particularly *Bifidobacterium*), alongside an enrichment of Proteobacteria and opportunistic taxa such as *Klebsiella* and *Enterococcus* [185, 186, 190]. Although microbial diversity and overall composition may partially recover over time, residual compositional and functional alterations can remain detectable for up to 1 year of age [190]. From a developmental perspective, intrapartum antibiotics therefore exemplify a central tension in perinatal care. Immediate anti‐infective benefits may come at the cost of disrupting early microbiome maturation. This does not argue against antibiotic use when clearly indicated. Rather, it supports more precise use, including clear indications, preference for narrow‐spectrum agents, and the shortest effective duration. Adjunctive strategies such as probiotics and prebiotics are being explored to support post‐exposure recovery; however, evidence remains heterogeneous. The priority is therefore not antibiotic avoidance, but antibiotic stewardship that protects both infection control and early microbial development.

#### Postnatal care environment

The postnatal care environment is not merely the setting in which infant colonization occurs, but an active ecological filter during a highly plastic developmental window [169, 191]. Through routine caregiving practices and repeated environmental exposures, it influences which maternal and environmental microbes are encountered, reinforced, and ultimately retained. Among modifiable practices, immediate postnatal skin‐to‐skin contact, known as kangaroo mother care (KMC), supports microbial stability. This includes enhanced maternal microbial transfer, reduced neonatal stress responses, thermoregulation, and earlier initiation of breastfeeding [169]. Randomized evidence shows that immediate KMC improves survival in low‐birth‐weight infants, and a post‐hoc analysis of the same multicenter trial found that immediate KMC did not increase, and may reduce, neonatal sepsis risk [192, 193]. Additionally, skin‐to‐skin contact has been reported to reduce postpartum depressive symptoms and maternal physiological stress [194]. Given evidence that maternal postnatal psychosocial distress is associated with variation in the human milk microbiome, these maternal physiological pathways may indirectly influence early microbial succession [195].

Other routine practices operate in a similar direction. Delayed bathing preserves vernix caseosa, which contains antimicrobial peptides and contributes to the newborn's innate skin barrier; whether this directly shapes gut microbial colonization remains insufficiently tested [196]. Exclusive breastfeeding offers continuous microbial and metabolic input, while rooming‐in increases repeated exposure to maternal microbial sources [173, 197]. In contrast, environmental reservoirs can exert opposing pressures. Hospital surfaces, medical equipment, and healthcare personnel can seed early communities with healthcare‐associated taxa and antimicrobial resistance determinants [198]. Conversely, household exposures (e.g., pets, siblings, outdoor environments) tend to increase microbial diversity and ecological resilience [199]. This contrast is especially relevant after cesarean delivery, when reduced maternal microbial transfer can increase reliance on environmental sources, especially those within hospital settings [185, 191].

Overall, postnatal care can either reinforce or counteract delivery mode‐related differences in early microbiome assembly. More broadly, early microbiome development reflects the combined influence of birth mode and postnatal environmental and caregiving contexts.

### Limitations and future perspectives

Despite substantial progress, research on the intrapartum and early postpartum microbiome remains limited by observational designs, heterogeneous sampling, and residual confounding from co‐exposures including delivery mode, antibiotic use, feeding practices, and the hospital environment. Consequently, source attribution and strain‐level transmission are often inferred rather than directly demonstrated. Furthermore, the long‐term clinical relevance of early microbial shifts remains uncertain. Interventional evidence, including microbiome‐targeted approaches, is further constrained by small sample sizes, limited standardization, and short follow‐up. This leaves uncertainty about the durability of observed effects. Large, well‐controlled trials are needed to evaluate whether antibiotic stewardship, breastfeeding support, skin‐to‐skin care, and microbiome‐targeted interventions can enhance microbial resilience safely. Methodological harmonization and longer follow‐up are essential for translation into perinatal practice, particularly in preterm infants who are especially vulnerable.

### Preterm infants

**FIGURE 4:**
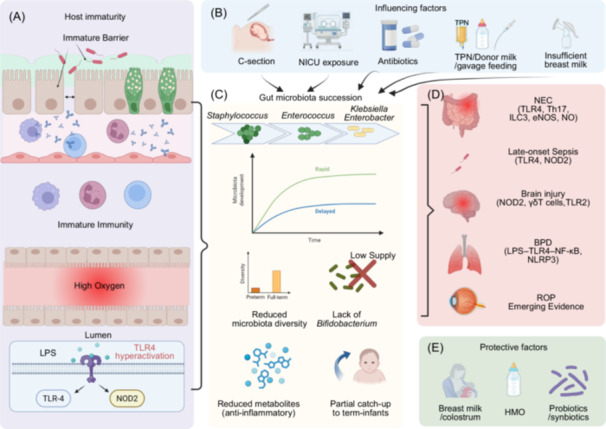
Mechanisms of preterm gut dysbiosis, exogenous pressures, and contributions to multi‐organ complications. (A) Host immaturity. Prematurity creates a developmentally immature intestinal niche in which barrier integrity, mucosal defense, immune sensing, and oxygen balance are not yet coordinated to support anaerobic microbial succession. Reduced intestinal alkaline phosphatase weakens lipopolysaccharide (LPS) detoxification, whereas elevated Toll‐like receptor 4 (TLR4) and altered nucleotide‐binding oligomerization domain‐containing protein 2 (NOD2)‐related sensing make the mucosa highly responsive to microbial ligands generated as dysbiosis develops. (B) Exogenous pressures. Cesarean section (C‐section), prolonged neonatal intensive care unit (NICU) exposure, broad‐spectrum antibiotics, total parenteral nutrition (TPN), reliance on donor milk or gavage feeding, and insufficient mother's own milk divert microbial assembly away from the full‐term, vaginally delivered, breastfed reference trajectory shaped by maternal transmission and human‐milk‐supported maturation. These pressures favor hospital‐associated facultative anaerobes and pathobionts while delaying milk‐adapted commensals. (C) Dysbiotic succession. Prematurity delays and distorts the normal maturation of the gut microbiota. Early communities often remain dominated by facultative anaerobes such as *Staphylococcus* and *Enterococcus*, with subsequent or persistent expansion of Enterobacteriaceae, including *Klebsiella* and *Enterobacter*, whereas recruitment of milk‐adapted anaerobes, especially *Bifidobacterium*, is postponed. This delayed trajectory is accompanied by reduced diversity, pathobiont enrichment, and depleted short‐chain fatty acid (SCFA) production. Although the preterm microbiota may shift toward a term‐like configuration, this catch‐up does not necessarily restore early microbial milestones or full functional maturity. (D) Multi‐organ complications. Dysbiosis, barrier immaturity, and metabolite loss provide a shared background for necrotizing enterocolitis (NEC), late‐onset sepsis (LOS), brain injury, bronchopulmonary dysplasia (BPD), and retinopathy of prematurity (ROP). NEC is most directly linked to Proteobacteria expansion and SCFA depletion in a TLR4‐high immature intestine, leading to epithelial injury, T helper 17 (Th17)/group 3 innate lymphoid cell (ILC3) inflammation, and impaired endothelial nitric oxide synthase (eNOS)–nitric oxide (NO) protection. LOS extends beyond the classical translocation model: although colonizing pathogens may cross an immature barrier, discordance between gut‐dominant taxa and bloodstream isolates suggests a broader role for dysbiosis‐driven immune dysfunction, including microbiota immaturity, reduced muramyl dipeptide (MDP)–NOD2 signaling, and weakened TLR4–myeloid differentiation primary response 88 (MyD88)–ILC3/granulocyte colony‐stimulating factor (G‐CSF)‐dependent granulopoiesis. BPD involves gut–lung inflammation via LPS–TLR4–nuclear factor kappa‐light‐chain‐enhancer of activated B cells (NF‐κB) signaling and SCFA–G‐protein‐coupled receptor 43 (GPR43) loss with NOD‐like receptor family pyrin domain containing 3 (NLRP3) activation. Brain injury is linked to altered metabolites, peptidoglycan sensing, and γδ T‐cell trafficking; ROP remains emerging. (E) Protective factors. Breast milk/colostrum, human milk oligosaccharides (HMOs), and strain‐defined probiotics or synbiotics may counteract dysbiosis, support *Bifidobacterium*‐centered maturation, restore protective metabolites, and redirect the preterm microbiota toward a developmentally synchronized trajectory.

In healthy full‐term infants, gut colonization generally follows a coordinated process shaped by maternal transmission, feeding, and physiological maturation. In preterm infants, however, this orderly progression is interrupted by both intrinsic immaturity and intensive medical care. This mismatch is increasingly recognized as a key mechanistic link between prematurity and major complications. Accordingly, the preterm microbiome should be understood not only as a taxonomic composition but also as a dynamic reflection of the developmental state (Figure 4).

### Characteristics of the preterm infant gut microbiota

#### The “gold standard” versus preterm divergence

The fecal microbiota of healthy, full‐term, vaginally delivered, and exclusively breastfed infants is widely regarded as the “gold standard.” It represents a stereotypical successional trajectory toward a mature, milk‐adapted, *Bifidobacterium*‐rich anaerobic community. In preterm infants, however, this trajectory is delayed and distorted. Their gut microbiota typically shows lower diversity, greater inter‐individual variability, and a persistent dominance of facultative anaerobes, particularly Enterobacteriaceae and *Enterococcus*. Conversely, beneficial anaerobes such as *Bifidobacterium* and *Bacteroides* are delayed or depleted [200, 201, 202, 203].

The divergence of the preterm microbiome should be interpreted as a problem of developmental timing rather than only as an altered taxonomic profile. Gut microbiome assembly in preterm infants is fundamentally constrained by profound physiological immaturity and the cumulative pressures of intensive clinical care [200]. Recent studies have used large‐scale cohort data to derive microbiome‐age models that infer postnatal age from microbiome features and evaluate the maturity status of the gut microbiota [204]. In preterm cohorts, lower microbiota age, or microbiota immaturity, has been independently linked to an increased risk of preterm complications [205]. Longitudinally, the preterm gut microbiota appears to follow a catch‐up trajectory, although the timing of convergence with term infants varies across cohorts. Partial attenuation of preterm–term differences has been reported during late infancy, by 6–12 months of chronological age [202, 206, 207]. However, preterm–term differences may still be detectable at 6 months in some cohorts [208], whereas evidence extending to 2 years suggests progression toward a more mature microbiome configuration [209]. At 3.5–4 years, prematurity‐associated microbial signatures may remain detectable [210, 211, 212]. These residual signatures suggest that early‐life prematurity may leave a detectable microbial imprint. Importantly, compositional convergence should not be equated with full functional recovery. Even when the microbiota becomes more similar to that of term infants, the disruption of key microbial milestones during critical early‐life windows may leave residual functional vulnerabilities and contribute to later health risks.

#### Host developmental immaturity and gestational age (GA) dynamics

Preterm infants harbor an unfavorable intestinal environment that promotes delayed microbial succession and pathobiont colonization because of profound gastrointestinal immaturity. Unlike full‐term neonates, preterm infants exhibit structural and functional deficits. These deficits generate a highly permeable and inherently pro‐inflammatory mucosal milieu [210]. Structural defects, including underdeveloped Paneth and goblet cells, reduced antimicrobial peptide secretion, a thinner mucus layer, immature tight junctions, and limited epithelial repair capacity, weaken barrier function and increase susceptibility to injury [210, 213, 214]. Immunologically, the preterm gut is also highly reactive. Low intestinal alkaline phosphatase reduces the ability to detoxify LPS, while elevated Toll‐like receptor 4 (TLR4) expression predisposes the immature intestine to exaggerated nuclear factor kappa‐light‐chain‐enhancer of activated B cells (NF‐κB)‐mediated inflammatory responses upon early microbial exposure [210, 215]. Deficient secretory immunoglobulin A (sIgA), sparse lamina propria lymphocytes, increased baseline inflammatory mediators, and persistently high luminal oxygen tension further compromise mucosal homeostasis and inhibit the establishment of obligate anaerobes [216]. Together, these features suggest that host developmental immaturity is an important driver of delayed microbial succession in preterm infants. Accordingly, these gestational‐age strata, reflecting different degrees of developmental maturity, should not be viewed merely as clinical categories. Rather, they represent distinct developmental–ecological niches shaped by barrier maturity, luminal oxygen tension, immune readiness, and the intensity of postnatal exposures.

#### Extremely preterm (EP, GA < 28 weeks)

Extremely preterm (EP) infants represent the most profound paradigm of gut microbiome immaturity and opportunistic pathogen colonization, a vulnerability consistent with their highest risk of severe complications and mortality [217]. Their gut community is rapidly dominated by opportunistic facultative anaerobes, particularly *Klebsiella* and *Enterococcus*, during postnatal Weeks 2–4 [202, 218, 219]. In extremely preterm infants, gut bacterial succession follows a patterned progression influenced by gestational or postmenstrual age, often transitioning from early facultative anaerobe‐dominated communities toward later anaerobic taxa [220, 221]. In contrast, *Bifidobacterium* recruitment is markedly delayed during the first month and only approaches term‐infant levels at around postnatal Day 120, indicating a prolonged catch‐up process [202]. This taxonomic delay is paralleled by functional immaturity. The microbiome retains an early carbohydrate‐focused metabolic profile before shifting toward amino acid and fatty acid metabolism near the corrected term age [222].

#### Very preterm (VP, GA 28–32 weeks)

Very preterm (VP) infants also pass through an early phase of opportunistic colonization, but their recovery trajectory is generally more resilient than that of EP infants. Early colonizers such as *Acinetobacter* and *Prevotella* are progressively replaced by *Enterococcus*, *Klebsiella*, and *Escherichia* between 2 and 6 weeks after birth. These taxa become dominant by Day 42 [223]. Compared with EP infants, however, VP infants show an earlier recovery of beneficial taxa, as *Bifidobacterium* usually begins to increase from around Day 14 [202].

#### Late and moderate preterm infants (LMPT, GA ≥ 32 weeks)

From initial colonization, late and moderate preterm (LMPT) infants exhibit a baseline microbial profile most closely resembling that of full‐term infants [201]. During the first 6 postnatal weeks, they show a higher abundance of *Bifidobacterium*, *Bacteroides*, and *Streptococcus*, with a microbiome dominated by Firmicutes rather than Proteobacteria [207]. Although minor taxonomic differences from term infants may persist at 1 month, these differences are generally resolved by 6 months after birth [207].

#### Dysbiosis driven by exogenous pressures

Foundational developmental influences such as delivery mode (e.g., the loss of maternal vaginal seeding via cesarean section) and maternal peripartum complications exert strong baseline pressures (as discussed in the previous section) [200, 224]. However, the overarching preterm successional trajectory is profoundly diverted by intensive postnatal clinical interventions within the hospital environment.

#### The neonatal intensive care unit environment and feeding pattern

For extremely preterm infants, the neonatal intensive care unit (NICU) becomes a major microbial reservoir because maternal microbial transfer is largely interrupted after birth. Strain‐level studies show extensive sharing between NICU surfaces and the preterm gut, with frequent colonization by hospital‐adapted opportunists such as *Staphylococcus epidermidis*, *Enterococcus faecalis*, *Pseudomonas aeruginosa*, and *Klebsiella pneumoniae* [198, 225]. Some strains can persist in the hospital environment and later colonize different infants [198]. Machine‐learning analyses further support this transmission pattern by identifying room‐specific microbial signatures that overlap with the gut microbiota of infants cared for there [225]. Clinical trials also suggest that environmental acquisition is strong enough for placebo‐group infants to acquire probiotic strains, especially when cared for in the same neonatal unit [226, 227, 228].

Feeding modality provides a major counterpressure to this hospital‐driven trajectory. Maternal milk acts in a dose‐dependent manner [229] and counteracts preterm gut immaturity by driving earlier microbial succession [230]. A lack of breastfeeding is another major ecological determinant of the preterm infant gut microbiome and is correlated with higher risks of major preterm complications [231, 232, 233, 234, 235]. However, profound gastrointestinal immaturity and early feeding intolerance often require alternative nutritional support. This may reshape the microbiome less favorably. Total parenteral nutrition is associated with a depletion of major commensal phyla and barrier‐supporting organisms such as *Akkermansia muciniphila*, together with an enrichment of opportunistic pathogens including *Escherichia*, *Salmonella*, and *Vibrio* [236]. Current evidence suggests that milk origin, especially mother's own milk versus donor human milk, has a stronger influence on the preterm gut microbiome than the fortifier itself [237, 238]. Holder pasteurization of donor milk further reduces indigenous microbes and alters its ecological profile compared with fresh maternal milk [239].

#### Antibiotic disruption and the resistome burden

Antibiotics are pervasive and ecologically disruptive interventions in the NICU. Because preterm infants have profoundly immature immune systems and are highly susceptible to infection, they often cannot be confidently distinguished from infants with early‐onset sepsis (EOS). Consequently, antibiotics are frequently used, with a substantial proportion of infants receiving prolonged empirical courses [240, 241]. Antibiotic exposure leads to perturbation of ecological balance of gut microbiota [204, 242, 243, 244]. The duration of exposure is a key determinant: very low birth weight infants exposed for at least 3 days show reduced *Bifidobacterium* abundance and lower α‐diversity at 2 and 4 weeks after birth compared with those receiving shorter courses [245]. Infants with delayed gut microbiome succession also have longer antibiotic exposure by postnatal Day 9 than those with rapid succession [205]. Broad‐spectrum agents such as meropenem, cefotaxime, and ticarcillin–clavulanate cause greater loss of richness than narrower regimens [246]. Meanwhile, co‐amoxiclav and gentamicin regimens can exert persistent effects, with lower α‐diversity detectable up to 1 year after birth [247].

Beyond compositional disruption, sustained antibiotic pressure can transform the preterm gut into a reservoir of antimicrobial resistance genes. Compared with term infants, preterm infants carry a heavier burden of multidrug, polymyxin, and glycopeptide resistance genes, often enriched in opportunistic pathogens including *Staphylococcus haemolyticus*, *Escherichia coli*, and *Enterococcus faecalis* [248, 249].

Taken together, these findings indicate that preterm dysbiosis is not solely an intrinsic consequence of prematurity. It is also shaped by exogenous environmental and clinical pressures in the NICU. Ecological correction strategies, including antibiotic stewardship, optimization of human milk feeding, and strain‐defined probiotic supplementation, may help redirect the preterm gut microbiota toward a more mature developmental trajectory. Antibiotic stewardship can limit avoidable dysbiosis and resistome expansion [248, 249]. Simultaneously, the prioritization of mother's own milk [230] helps preserve a milk‐adapted successional trajectory. Strain‐defined probiotics may further support microbiome maturation in preterm infants [227]. However, their effects are likely influenced by individual clinical and ecological contexts.

#### Preterm complications

Although necrotizing enterocolitis (NEC), late‐onset sepsis (LOS), bronchopulmonary dysplasia (BPD), retinopathy of prematurity (ROP), and brain injury manifest as distinct organ‐specific pathologies, they share a convergent dysbiosis background. This background includes reduced α‐diversity, Proteobacteria and Enterobacteriaceae accumulation, *Bifidobacterium* depletion, reduced SCFA production, disturbed BA homeostasis, and weakened barrier integrity. These shared features suggest that major preterm complications may represent organ‐specific manifestations of a broader microbiome–host developmental mismatch. However, the strength of causal evidence varies across diseases.

#### Necrotizing enterocolitis

NEC remains one of the most devastating gastrointestinal complications in very low birth weight (VLBW, <1500 g) infants. It has an incidence of 2%–10% and a clinical course that can rapidly progress from feeding intolerance to systemic shock [250]. Although the precise etiology of NEC remains incompletely understood, accumulating evidence supports an important role of the gut microbiome in its pathogenesis. NEC is preceded by a characteristic microbial pattern with a marked decline in microbial diversity, expansion of Proteobacteria, and depletion of beneficial commensals [231, 251, 252, 253]. This shift is accompanied by reduced protective metabolites, particularly SCFAs [254]. Beyond taxonomic imbalance, the accelerated replication of Enterobacteriaceae has been linked to impending NEC [252]. Notably, resistome dynamics may outperform taxonomic composition in predicting disease risk. For instance, the convergence of resistance profiles has been observed up to 9 days before NEC onset [248]. Maternal milk composition further modifies risk, as low concentrations of the protective oligosaccharide disialyllacto‐N‐tetraose (DSLNT) are associated with increased NEC susceptibility [231].

Mechanistically, this dysbiotic state promotes NEC largely through exaggerated TLR4 activation triggered by LPS‐rich Proteobacteria [255]. In the immature intestine, excessive TLR4 signaling drives epithelial apoptosis, autophagy, and necroptosis, thereby amplifying mucosal injury [255]. Excessive TLR4 signaling also reshapes local immunity by promoting pro‐inflammatory responses, including interleukin‐6 (IL‐6) and interleukin‐1β (IL‐1β) production by T helper 17 (Th17) cells and group 3 innate lymphoid cells (ILC3s). Furthermore, it increases Th1 cells and reduces Tregs [256]. These inflammatory injuries are further worsened by reduced endothelial nitric oxide synthase expression and diminished nitric oxide availability, which impair vasodilation and contribute to ischemic necrosis [257]. Microbial–metabolic intervention studies support this mechanistic interpretation. For example, *Bacteroides fragilis* carrying bile salt hydrolase (*bsh*) genes can reduce intestinal injury by lowering cytotoxic taurochenodeoxycholic acid through the FXR–NLRP3 pathway [258]. Additionally, *Lactobacillus gasseri* FWJL‐4 attenuates NEC via acetate production, barrier reinforcement, mucin 2 (MUC2) upregulation, and inhibition of ileal necroptosis through G‐protein‐coupled receptor 41 (GPR41) and G‐protein‐coupled receptor 43 (GPR43) signaling [259]. Clinically, meta‐analyses suggest that multi‐strain probiotics containing *Lactobacillus* and *Bifidobacterium* are most effective in reducing NEC and all‐cause mortality [260]. Furthermore, the ProPrem trial showed that a combination of *Bifidobacterium infantis*, *Streptococcus thermophilus*, and *Bifidobacterium lactis* reduced NEC as a secondary outcome in VLBW infants [261].

#### Late‐onset sepsis

Translocation of intestinal pathogens across an impaired mucosal barrier is considered one important mechanism of LOS. Colonization by Gram‐negative pathogens often precedes bloodstream infection [262, 263, 264]. This process can be directly visualized in experimental systems with bioluminescence imaging [216]. However, discrepancies between dominant gut taxa and bloodstream isolates suggest that translocation alone does not fully explain LOS pathogenesis [265]. In addition, gut dysbiosis may compromise host immune maturation and immune homeostasis through several routes [266]. Infants who later develop LOS often show reduced α‐diversity, expansion of Proteobacteria, depletion of obligate anaerobes, and a delayed microbiome developmental trajectory. This delay has emerged as an independent predictor of LOS risk [205, 262, 267, 268].

Gut dysbiosis may compromise host immune homeostasis through several routes. The absence of DL‐endopeptidase‐producing commensals such as *Enterococcus faecium* may limit muramyl dipeptide (MDP) generation. This limitation results in inadequate nucleotide‐binding oligomerization domain 2 receptor (NOD2) activation and defective cylindromatosis (CYLD)–NF‐κB‐mediated immune tolerance [205]. In parallel, antibiotic‐induced microbiota disruption can weaken intestinal signaling mediated by TLR4 and myeloid differentiation primary response 88 (MyD88), while reducing IL‐17‐producing ILC3s and granulocyte colony‐stimulating factor levels. This impairment consequently reduces bone marrow granulopoiesis and peripheral neutrophil recruitment, increasing susceptibility to *Klebsiella pneumoniae* and *Escherichia coli* K1 sepsis [269]. These dysbiosis‐linked immune alterations provide a rationale for microbiome‐directed interventions to reduce LOS risk. In clinical studies, *Lactobacillus plantarum* plus fructooligosaccharides significantly reduced the combined outcome of LOS and death in a large randomized trial involving 4556 infants, including late preterm infants [270]. Mechanistically, in preterm infants, administration of DL‐endopeptidase‐producing *Limosilactobacillus reuteri* DSM 17938 restored NOD2 activation capacity via MDP generation. Concurrently, *Enterococcus faecium* provides parallel mechanistic support from mouse models [205]. Nevertheless, most probiotic‐based strategies have not yet shown consistent reductions in overall LOS incidence [260].

#### Bronchopulmonary dysplasia

BPD results from the interplay of pulmonary immaturity, oxidative stress, and systemic inflammation. Emerging evidence highlights the gut–lung axis as a crucial modulator of this inflammatory cascade. Infants who later develop BPD typically exhibit a dysbiotic gut signature characterized by reduced microbial diversity, enrichment of Proteobacteria, and depletion of beneficial commensals [271, 272]. This ecological shift may drive distant lung injury through several interconnected pathways. Gut Proteobacteria‐derived LPS systemically activates pulmonary TLR4 and NF‐κB signaling [273]. Depletion of commensal‐derived SCFAs further compromises GPR43‐mediated anti‐inflammatory defenses, promoting NLRP3 inflammasome activation, IL‐1β and interleukin‐18 (IL‐18) release, and pyroptosis [274]. Gut dysbiosis may also mobilize immune cells, promoting migration of activated group 2 innate lymphoid cells (ILC2s) and ILC3 cells to the lung and amplifying Th17‐associated inflammation. This amplification thereby disrupts normal alveolarization [275]. Although targeted strains such as *Lactobacillus plantarum* L168 can attenuate hyperoxia‐induced lung injury in animal models [276], clinical evidence remains inconsistent. Currently, meta‐analyses do not support a universal protective effect of probiotics against BPD [277, 278].

#### Retinopathy of prematurity

Evidence implicating the gut microbiome in ROP remains suggestive but less mature than that for NEC or LOS. Small‐scale cohort studies indicate that gut–retina signaling may be mechanistically relevant. These studies have observed Enterobacteriaceae and *Staphylococcus* accumulation alongside *Bifidobacterium* deficiency in infants with ROP [279, 280]. However, it remains unclear whether this dysbiosis pattern is a true mechanistic driver or a marker of population‐specific risk and treatment exposures. Robust mechanistic validation and targeted clinical interventions focused on this gut–retina axis remain largely lacking.

#### Brain injury

Premature infants are exceptionally vulnerable to neurological morbidities because critical windows for neurogenesis coincide with the second and third trimesters [281]. Gut dysbiosis, marked by *Klebsiella* enrichment and *Bifidobacterium* depletion [282, 283], can rapidly translate into a disruptive metabolic and immune microenvironment. Increased plasma levels of tryptamine and galactitol by postnatal Day 3 and elevated fecal indoxyl sulfate by Day 7 have been identified in infants with brain injury [222]. Opportunistic pathogens such as *Klebsiella* exploit siderophore‐mediated iron acquisition and nitrate respiration to outcompete indigenous commensals. This exploitation strips the ecosystem of functional redundancy [283]. This compositional and functional dysbiosis reprograms fecal metabolism, depleting neuroprotective agents such as gamma‐aminobutyric acid (GABA), 5‐hydroxytryptamine (5‐HT), and N‐acetylaspartate (NAA) and increasing microbiome‐derived chenodeoxycholic acid. This elevation may promote a leaky gut state [222]. When intestinal barrier integrity is compromised, circulating gut‐derived lysophosphatidylcholines can increase and induce cerebral oxidative damage [284]. Microbiome‐derived peptidoglycan fragments may activate microglial NOD2 and TLR2 signaling and damage myelinating oligodendrocytes [285]. In parallel, intestinal *Klebsiella* accumulation can drive peripheral interleukin‐17A (IL‐17A)‐producing γδ T cells to migrate into the central nervous system (CNS) and exacerbate white matter hypoxic–ischemic injury [282].

### Current limitations and future perspectives

Despite the compelling mechanistic frameworks discussed above, microbiome research in preterm infants faces distinct clinical and methodological limitations that complicate causal interpretation. First, the intense medicalization of the NICU creates a uniquely confounding exposome. Beyond antibiotics, EP and VLBW infants are routinely exposed to prolonged positive‐pressure ventilation, frequent nil per os periods, exogenous surfactants, cyclooxygenase inhibitors for patent ductus arteriosus, and methylxanthines such as caffeine. The independent and combined effects of these interventions on mucosal microecology remain poorly characterized. This makes it difficult to distinguish microbial causality from iatrogenic effects [200]. Second, prematurity also introduces a fundamental chronobiological confounder, because preterm microbiome age reflects both postnatal and gestational age. Many cohort studies do not adequately separate these timelines, obscuring whether milestones such as delayed *Bifidobacterium* expansion are driven primarily by NICU exposure or by host developmental readiness. Third, the very low microbial biomass in EP infants during the first week of life makes early critical‐window sampling highly vulnerable to reagent contamination and NICU environment contamination. This blurs the distinction between true pioneer engraftment and transient hospital‐derived DNA signals [156]. Finally, modeling preterm morbidities in vivo remains challenging. Traditional murine models do not faithfully recapitulate human preterm complications, and developmental differences between mice and infants born at 24–28 weeks of gestation may overestimate mucosal resilience. More complex translational models, including preterm piglets and human‐derived neonatal organoids, are therefore needed [286].

Given these limitations, the current evidence base is strongest for microbiome‐centered involvement in NEC and LOS. Brain injury and BPD show increasingly persuasive mechanistic and human cohort evidence but remain more multicausal. ROP is biologically plausible and clinically promising, but is still earlier in evidentiary development.

In preterm infants, the critical question is not simply which organisms are present, but whether microbial succession is synchronized with host maturation. Future studies should therefore stage infants simultaneously by GA, postmenstrual age, and chronological age. Furthermore, they should prioritize clinically meaningful endpoints. These include microbiome age lag, pathobiont bloom behavior [252, 253], resistome burden [248], BA and SCFA deficiency [254], and the failure of strain‐level functional programs such as DL‐endopeptidase production [205]. Ultimately, the preterm gut microbiome should be understood as a state of host–microbiome developmental mismatch. In this state, microbial succession, immune–metabolic maturation, and NICU‐related exposures become temporally uncoupled during a vulnerable developmental window.

## INFANCY–CHILDHOOD

### Gut microbiota development

**FIGURE 5:**
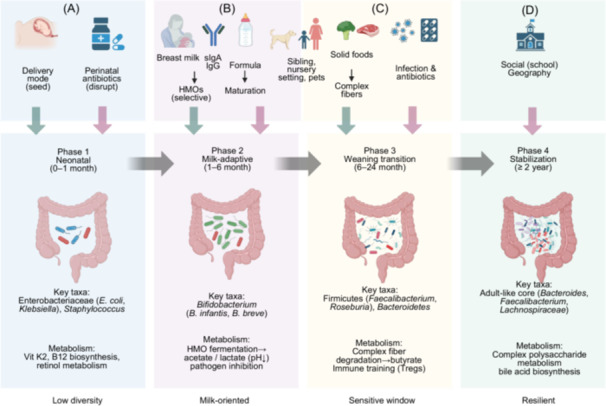
Successional dynamics, functional maturation, and multi‐dimensional drivers of the early‐life gut microbiome. (A) Phase 1: Neonatal (0–1 month). The initial colonization phase is a low‐diversity ecosystem characterized by the establishment of aerotolerant pioneer taxa, notably the family Enterobacteriaceae (including *Escherichia coli* and *Klebsiella*) and the genus *Staphylococcus*. The primary ecological drivers during this window are the delivery mode, which seeds the foundational microbiota, and the disruptive potential of perinatal antibiotics. Functionally, this transient community is enriched in metabolic pathways essential for early development, including vitamin K2 and B12 biosynthesis, as well as retinol metabolism. (B) Phase 2: Milk‐adaptive (1–6 months). Driven by the selective pressures of infant feeding and household exposures (e.g., siblings, pets, and nursery settings), the microbiota transitions to a milk‐oriented, strict anaerobic state. Breast milk supplies essential immunological factors (sIgA and IgG) and human milk oligosaccharides (HMOs), which serve as selective prebiotics that drive the absolute dominance of *Bifidobacterium* species (e.g., *B. infantis*, *B. breve*). The fermentation of HMOs produces acetate and lactate, lowering luminal pH and providing robust pathogen inhibition. Conversely, formula feeding can drive premature microbial maturation. (C) Phase 3: Weaning transition (6–24 months). The introduction of solid foods provides complex fibers that prompt a critical ecological shift, reducing *Bifidobacterium* dominance in favor of Bacteroidetes and Firmicutes (e.g., *Faecalibacterium* and *Roseburia*). This sensitive window is defined by a functional transition toward complex fiber degradation and the production of butyrate, which is vital for immune training and the generation of regulatory T cells (Tregs). Environmental perturbations such as infections and antibiotic use during this phase can significantly disrupt immune–microbiome crosstalk. (D) Phase 4: Stabilization (≥2 years). The gut microbiome matures into a highly diverse, resilient, and adult‐like ecosystem. It is increasingly shaped by broader environmental determinants, including social interactions (e.g., school/nursery) and geographical location. Taxonomically, the community stabilizes into an adult‐like core dominated by *Bacteroides*, *Faecalibacterium*, and Lachnospiraceae. Functionally, the microbiota is optimized for complex polysaccharide metabolism and secondary bile acid biosynthesis, establishing a lifelong regulatory hub for host homeostasis.

The development of the infant gut microbiota is a predictable, nonrandom process of ecological succession. It transitions from an initial state of low diversity, high instability, and dominance by facultative anaerobes to a complex, resilient ecosystem dominated by obligate anaerobes and capable of supporting host homeostasis. This trajectory is characterized by key developmental milestones that mirror the physiological maturation of the host, suggesting a co‐evolutionary imperative [148, 163, 174, 287, 288, 289]. Understanding these successional dynamics requires viewing the infant gut not just as a passive receptacle for bacteria, but as a dynamic ecosystem [290]. The gut microbiota follows general developmental trajectories. However, individual gut microbiome development is shaped by specific external factors. Drivers such as initial colonization, nutrition, medical history, and environmental context exert selective pressures that can accelerate, delay, or permanently divert the developmental course of the microbiome (Figure 5).

### Successional dynamics and functional maturation of the early‐life gut microbiome

#### Initial colonization: The transition from chaos to anaerobiosis (birth to ~1 month)

The neonatal gut is initially a heterogeneous and ecologically chaotic environment, characterized by high inter‐individual variability and low ecological stability [291]. Immediately after birth, the intestinal lumen contains residual oxygen, creating a transient aerobic or microaerophilic niche. This condition fundamentally differs from the strict anaerobic environment of the adult gut and acts as a primary ecological determinant. Consequently, the initial colonizers are predominantly aerotolerant pioneer species, mainly facultative anaerobes belonging to the Enterobacteriaceae family (such as *Escherichia coli* and *Klebsiella*) as well as *Staphylococcus* and *Streptococcus* species [163, 287, 288]. This physiological modification facilitates the subsequent establishment of strict anaerobes, including *Bifidobacterium*, *Bacteroides*, and *Clostridium* [163, 173]. The failure to effectively establish this anaerobic niche, which is often observed in preterm infants or following antibiotic exposure, may impede the maturation of the entire ecosystem and potentially sustain the dominance of opportunistic pathogens.

The neonatal microbiome functions as a vital metabolic factory adapted to the specific developmental needs of the infant. During the first month, the microbiome is significantly enriched in genes encoding vitamin K2 (menaquinone) biosynthesis, primarily contributed by *Bacteroides* and *Escherichia/Shigella* [173]. Furthermore, the neonatal microbial metabolism shows a distinct enrichment in pathways for retinol (vitamin A) metabolism and folate biosynthesis. These pathways are critical for the development of vision, bone growth, and rapid cellular proliferation. The upregulation of bacterial transport systems for iron, hemin, and heme supports vitamin B12 synthesis, which plays a vital role in infant iron metabolism and brain development [173, 292]. This suggests that the pioneer microbiota may play an unappreciated role in regulating early neural development. Thus, the initial colonization represents a critical window of metabolic programming, where the transient microbial community actively shapes the host's developmental trajectory.

#### The milk‐adapted phase: *Bifidobacterium* dominance and host–microbe crosstalk (~1 to 6 months)

As the pioneer species deplete luminal oxygen, the gut ecosystem undergoes its first major taxonomic turnover during this phase. The relative abundance of facultative anaerobes declines, favoring the growth of strict anaerobes. In healthy and breastfed infants, this phase is characterized by the dominance of the genus *Bifidobacterium*, particularly species such as *B. infantis*, *B. breve*, *B. bifidum*, and *B. longum* [163, 173, 204, 293, 294]. This dominance is driven by the unique composition of human breast milk. HMOs, the third most abundant solid component of milk, are complex glycans that are indigestible to the infant but serve as a premium, selective substrate for *Bifidobacterium* species [173, 295]. Notably, *Bifidobacterium longum* subsp. *infantis* possesses a large gene cluster dedicated to HMO metabolism. This allows it to efficiently consume several low‐molecular‐weight HMOs, a capability that is deficient in adult‐associated bifidobacteria, such as the closely related *B. longum* subsp. *longum* [296]. The fermentation of HMOs produces acetate and lactate, which acidify the gut lumen to inhibit the growth of pathogens [145, 173, 293, 297]. Another specific maternal milk component, sialylated milk oligosaccharides (SMOs), is metabolized by the gut microbiome to release bioactive compounds necessary for bone and muscle development. The deficiency of SMOs in maternal milk is potentially a key driver of growth failure in malnourished neonates [298]. During this period, gut microbial metabolism is prioritized for oxidative phosphorylation (energy production), lactate utilization, and the *de novo* synthesis of B‐vitamins, including riboflavin (B2), folate (B9), and biotin (B7) [173].

In contrast, infants who are fed formula harbor more bacteria belonging to Bacteroidetes and Firmicutes, such as *Bacteroides* and *Clostridium*. Consequently, they show a higher alpha diversity and a more mature microbial pattern compared with exclusively breastfed infants [288, 293, 298]. This microbiome is often characterized by adult‐like functions such as methanogenesis and secondary BA biosynthesis, metabolic activities that may be inappropriate for the immature gut mucosa [173]. Thus, formula induces a premature shift toward adult metabolism, which is not suitable for supporting the infant's biosynthetic and growth requirements [299]. Therefore, this milk‐adapted phase of gut microbiome development is vital for the proper maturation of the mucosal immune system and the endocrine pathways that govern long‐term health.

#### The weaning transition: Functional expansion and immune training (6 months to 2 years)

The introduction of solid foods (complementary feeding), typically beginning around 6 months of age, marks a pivotal ecological turning point. This phase involves the decline of *Bifidobacterium* dominance and the bloom of the phyla Firmicutes (specifically the Clostridia class, including *Faecalibacterium*, *Blautia*, *Roseburia*, and *Ruminococcus*) and Bacteroidetes [173, 174, 293, 300]. These taxa are functionally equipped with diverse carbohydrate‐active enzymes that help degrade complex dietary fibers introduced during complementary feeding [299].

The gut microbial metabolic function shifts from lactate/acetate production to the generation of butyrate and propionate [288, 301]. Butyrate serves as the primary energy source for colonocytes and is a potent regulator of immune homeostasis [173, 302]. Interestingly, the infant gut appears to be primed for this transition. Genes encoding plant‐glycan degradation enzymes begin to be expressed even before solid foods are introduced, suggesting an anticipatory adaptation mechanism encoded within the microbiome [173]. Furthermore, amino acid metabolism (specifically methionine degradation, lysine biosynthesis, and tryptophan metabolism) increases progressively, reaching maternal‐like levels by 12 months of age [299]. The weaning transition is a critical window for immune education. The bacterial metabolites (such as SCFAs) and dietary components (such as retinoic acid) jointly promote the generation of retinoic acid receptor‐related orphan receptor gamma (RORγ)+ Tregs, preventing pathological imprinting. Disruption of this process due to dietary changes or antibiotic use can result in increased susceptibility to immunopathologies later in life [303, 304]. With the introduction of solids, the gut microbiome gradually diversifies and develops a stronger capacity to degrade complex polysaccharides, ultimately shifting toward an adult‐like, Firmicutes‐dominated ecosystem [293, 300].

#### Stabilization and resilience: The establishment of an adult‐like ecosystem (>2 years)

By approximately 2 to 3 years of age, the profound fluctuations characteristic of infancy begin to subside. The gut microbiome stabilizes, converging towards an adult‐like composition [293, 305]. At this stage, the community is dominated by the genera *Bacteroides* and *Faecalibacterium*, along with diverse members of the Lachnospiraceae family [287, 293, 306]. By 3 years of age, infants in distinct developmental stages harbor microbial communities dominated by different bacteria, leading to significantly stratified patterns in global populations [307, 308]. Along with the maturation of the microbiome, functionalities associated with pathways of complex polysaccharide metabolism are significantly upregulated to better adapt to adult‐like lifestyles [302, 308]. However, the gut microbiota has not yet reached adult complexity in 5‐year‐old children [287].

#### Early‐life virome dynamics

The development of the infant gut virome, primarily composed of bacteriophages, follows a distinct successional pattern that mirrors and interacts with bacterial colonization. Unlike the bacterial community, where diversity increases progressively with age, the viral community exhibits a different trend: richness and diversity peak shortly after birth and gradually decline, eventually reaching a stabilized equilibrium around two to three years of age. This developmental arc is fundamentally characterized by a strategic shift from predatory lytic activity to a more stable, lysogenic symbiotic state.

Although the neonatal gut is virtually sterile at birth, it is rapidly colonized by a vast array of phages within the first week of life. During this early infancy stage, which is often referred to as the “lytic phase,” the virome is dominated by virulent phages that thrive through a “kill‐the‐winner” dynamic. These phages propagate by replicating in massive numbers and subsequently lysing their bacterial hosts to release progeny. Consequently, the neonatal virome is characterized by extreme fluctuations and high diversity. It comprises a significant number of phages derived from the environment and induced from early colonizing bacteria. Emerging evidence highlights that the initial viral seeds are primarily derived from the maternal vaginal tract, skin, and fecal microbiota [167]. Furthermore, breast milk acts as a continuous conduit for diverse viral populations, ensuring the early establishment of a virome that is uniquely tailored to the infant's immediate environment [309]. Clinical factors, such as delivery mode, play a pivotal role here. Infants born via cesarean section often experience a delayed or altered viral colonization compared to those born vaginally, potentially disrupting the early‐life immune priming [167, 310, 311].

As infants mature, particularly after the introduction of solid foods, the gut landscape is reshaped, and its taxonomic composition changes substantially. This transition is marked by a decline in the dominance of Caudovirales (tailed phages) and a concomitant rise in Microviridae [312, 313]. This period represents a critical ecological shift from a highly volatile, stochastic community toward a more structured and resilient viral–bacterial network.

By the age of two to three years, the virome converges toward a state of equilibrium reminiscent of the adult gut. As the absolute densities of both bacteria and phages surpass a critical ecological threshold, the lifestyle of the viral community transitions from lytic predation to lysogenic persistence. In this state, temperate phages preferentially integrate their genetic material into the host genome as prophages rather than initiating cellular destruction. This shift toward lysogeny results in a marked decrease in free viral diversity and the establishment of a stable, temperate‐phage‐dominated ecosystem [312]. Ultimately, this inherited and evolved virome serves as a critical reservoir for mobile genetic elements, supporting gut homeostasis and long‐term human health.

#### Early‐life mycobiome assembly

The human intestinal mycobiome, though representing only approximately 0.1% of the adult gut microbiota, plays a critical role in early‐life health and development. Fungal colonization commences at birth and follows a distinct successional trajectory. *Candida* is the most abundant genus at birth, typically replaced by *Malassezia* and *Cystofilobasidium* at 6 months, and *Trichosporon* by 18 months of age [165]. Evidence supports the vertical transfer of specific fungal taxa, such as *Candida albicans* and *Candida parapsilosis*, from the maternal oral cavity, breast milk, or gut to the infant [314]. While overlapping species are observed in mother–infant pairs [164], the full diversity of an infant's oral, gut, and skin mycobiome is not always congruent with the mother's. This suggests that external environmental sources also contribute to early‐life colonization [315].

Key modulators of this fungal development include the mode of delivery, antibiotic exposure, and feeding practices. Infants born vaginally exhibit a mycobiome dominated by *Trichosporon*, whereas those born via Cesarean section are dominated by *Saccharomyces* [165]. The genera *Saccharomyces*, *Trichosporon*, *Pezoloma*, *Cystofilobasidium*, *Rigidoporus*, and *Fomitopsis* are strongly associated with antibiotic exposure at birth [165, 316]. Furthermore, although *Candida* species have been detected in breast milk, and in vitro experiments have shown that the pathogenicity of fungi can be influenced by HMOs, it is hypothesized that breast milk also modulates the infant gastrointestinal mycobiota. This is a relationship that remains to be fully elucidated [317, 318].

Imbalances in the early‐life mycobiome are associated with various clinical outcomes. In premature neonates, initial characteristics of the intestinal mycobiome are closely linked to the subsequent development of BPD [319]. Compared to adults, infants are more susceptible to infections resulting from fungal overgrowth. This overgrowth can lead to significant morbidity and mortality in high‐risk groups such as preterm infants [320]. To address these dysbioses, certain species, such as *Saccharomyces boulardii*, have been shown to be effective in preventing and treating human gastrointestinal diseases, including diarrhea, inflammatory bowel disease, and irritable bowel syndrome [321, 322].

The transition from infancy to adulthood is a coordinated multikingdom assembly, where the synchronized succession of bacteria, viruses, and fungi forms a functional interactome that programs long‐term immune and metabolic homeostasis.

### Multi‐dimensional drivers of microbiome composition and function

#### Diet

Diet represents the most potent modulator of the gut microbiome. Breast milk serves a dual function as a microbial seed and a selective fertilizer. It acts as a continuous inoculum, introducing beneficial bacteria, including *Staphylococcus*, *Streptococcus*, *Lactobacillus*, and *Bifidobacterium*, directly into the infant gut [171]. Meanwhile, HMOs from breast milk feed *Bifidobacterium* species as fertilizer [295, 297]. Maternal fucosyltransferase 2 (FUT2) produces specific fucosylated HMOs (such as 2’‐fucosyllactose) that act as premium fuel for specific *Bifidobacterium* strains. Infants of non‐secretor mothers often show delayed colonization of these beneficial taxa and lower α‐diversity [323]. In many cesarean‐delivered infants, breastfeeding also counteracts imbalances in the gut microbiota by replenishing levels of *Bifidobacterium* species and promoting a microbial composition more similar to that of vaginally delivered infants [324].

Crucially, breast milk provides essential bioactive factors that establish immune homeostasis. IgA, the most abundant antibody in milk, promotes barrier function by limiting bacterial adhesion to the epithelium [325] and regulating virulence gene expression. For instance, it inhibits *Erysipelatoclostridium ramosum*–induced Th17 inflammation to prevent asthma [326, 327]. Maternal IgG in breast milk binds to commensal bacteria in the lumen, signaling the neonatal immune system to tolerate these beneficial microbes, while simultaneously coating pathogens to promote phagocytosis by neutrophils [328, 329]. This mechanism enables the infant to distinguish “friend” from “foe” during the critical window of tolerance induction.

Unlike breast‐milk‐fed infants, formula‐fed infants often exhibit lower levels of *Bifidobacterium* and a more diverse, mature microbial pattern compared with exclusively breastfed infants [330]. Formula feeding tends to accelerate microbial maturation, inducing a premature adult‐like state [173, 293, 299]. Nondigestible oligosaccharides added to formula have shown results similar to those of breastfeeding in reducing the colonic pH and increasing the production of SCFAs, effects that are probably associated with a selective stimulation of *Bifidobacterium* and *Lactobacillus* spp. [331].

The composition of solid foods, particularly micronutrient fortification, and the timing of solid food introduction play important roles in shaping the early‐life gut microbiome. Early complementary feeding (≤3 months) increases diversity and butyrate‐producing taxa such as Lachnospiraceae [332]. Breastfed infants introduced to solids before 5 months had increased microbiome diversity, including higher levels of *Bifidobacterium infantis* [333]. For children with pre‐existing dysbiosis, iron supplementation may worsen microbial imbalances [334]. Thus, nutritional interventions necessitate a careful assessment of the host's baseline ecosystem to avoid adverse shifts.

#### Antibiotics and infectious perturbations

The infant gut is highly susceptible to iatrogenic and pathological disturbances. Antibiotics represent the most profound perturbation to the developing microbiome [185, 244]. Early‐life exposure leads to a marked decrease in *Bifidobacterium* species (e.g., *B. bifidum*, *B. pseudocatenulatum*, *B. adolescentis*) and a bloom of Enterobacteriaceae [335, 336]. The impact manifests not only as temporary taxonomic shifts but also as profound functional impairments. Antibiotics cause a regression in the developmental trajectory, significantly reducing microbial diversity and stability [204]. These disruptions have profound immune consequences by reducing specific immune‐modulating microbes, such as segmented filamentous bacteria (SFB) and *Lactobacillus*, thus resulting in a blunted induction of intestinal Th17 cells [337, 338]. This immune defect is mechanistically linked to an increased risk of metabolic disorders and atopic diseases [337, 339, 340, 341].

Furthermore, infection acts as a potent disruptor of the gut microbiome. Acute gastroenteritis (e.g., rotavirus) causes osmotic diarrhea. This leads to a massive loss of microbial biomass and the physical erosion of the mucosal biofilm, thereby creating niches for opportunistic invasion [342]. Furthermore, host inflammation during infection selectively favors the growth of facultative anaerobes (e.g., Enterobacteriaceae) over strict anaerobes [343]. Some clinical evidence also suggests that it is the infant infection itself, rather than antibiotic use, that correlates most strongly with childhood obesity risk [344]. This indicates that the inflammatory state may imprint long‐term metabolic dysregulation.

#### Environmental exposures

The infant microbial assembly is continuously replenished by the external environment, where geographic location exerts a profound influence on the microbiome [345, 346]. A striking example of this is seen in the disparity of autoimmune diabetes rates between neighboring regions. Infants in Finland and Estonia, regions with a high autoimmunity prevalence, harbor *Bacteroides* species (e.g., *Bacteroides dorei*) that produce a structurally unique LPS. These specific LPS variants are tetra‐acylated or penta‐acylated, failing to adequately activate the immune system via TLR4 to establish endotoxin tolerance. In contrast, Russian infants harbor *Escherichia coli*‐derived LPS that is hexa‐acylated and immunologically active [346]. These geography‐related microbiome findings illustrate that immune modulation is driven by the structural immunogenicity of the microbiota, not just its density.

Beyond geographic location, specific environmental pollutants alter the microbial landscape through distinct xenobiotic exposures. Maternal arsenic exposure reduces infant microbial α‐diversity and depletes *Bifidobacterium* [347]. Conversely, childhood arsenic exposure promotes Proteobacteria and enriches the microbiome with virulence and multidrug resistance genes [348]. Children in e‐waste hubs are frequently exposed to dust‐borne contaminants, including 6PPD‐quinone (6PPDQ), polybrominated diphenyl ethers (PBDEs), polychlorinated biphenyls (PCBs), and metals like nickel (Ni) and copper (Cu). These exposures disrupt microbial metabolism, fueling neurotoxic 2,4‐diaminobutyric acid accumulation and protective lipid loss [349]. Furthermore, exposure to consumer chemicals like bisphenols and parabens impairs microbial methionine metabolism [350]. Emerging pollutants, such as microplastics, can also impair brain function by inhibiting key microbial metabolites such as trehalose [351]. Collectively, the early‐life microbiome acts as a pivotal mediator between chemical stressors and host developmental health.

Within the household, pets act as microbial conduits. Exposure to dogs during the first year is associated with reduced risks of type 1 diabetes (T1D) and allergic sensitization [352, 353]. This protective effect may be driven by the fact that house dust from dog‐owning homes is enriched with bacteria that foster *Lactobacillus johnsonii* abundance, thereby enhancing airway immune defense [354]. Additionally, parenting behaviors directly modulate this microbial transfer. For example, parental sucking of pacifiers not only alters the infant's oral microbiota composition but is also associated with a lower incidence of allergies. This underscores the protective value of shared family microbes [355].

Ricci et al. used a strain‐resolved longitudinal design (“microTOUCH‐baby”) to quantify how peer contact reshapes infant gut microbiome assembly during the first year of nursery [356]. By tracking strain‐level microbial exchange among infants and their social networks (parents, co‐living siblings, and nursery educators) via shotgun metagenomics, they revealed that nursery entry drives rapid, extensive horizontal transmission. Infant microbiomes progressively converge within classes and accumulate “nursery‐acquired” strains, indicating that social mixing outside the household becomes a major source of new colonizers during late infancy. Notably, pre‐existing siblings reduce nursery‐acquired strain accumulation, while antibiotic exposure significantly accelerates it [356]. Overall, these data position early‐life social interactions as a key ecological driver of microbiome maturation, with implications for how childcare environments, family structure, and antibiotic perturbations jointly shape microbial trajectories in infancy. Similarly, the presence of older siblings creates a sibling effect, associated with higher diversity and reduced atopic dermatitis risk [357]. These findings collectively suggest that a diverse, microbially rich environment acts as a protective factor, buffering the infant against the dysbiosis associated with modern, sanitized living conditions.

The relative impact of determinants is highly stage‐specific. During Phase 2 and Phase 3, the infant gut is characterized by an unstable ecological structure and low diversity. This creates an open niche where external microbial inputs from diet or household (siblings, pets, and nursery settings) can exert strong priority effects. These inputs effectively seed and train the developing microbiome. As the successional trajectory progresses into Phase 4, the microbiota reaches a relatively stable, adult‐like configuration. While the core network is more established than in earlier phases, these broader social and geographical exposures continue to actively shape and refine the microbiota. This ongoing influence reinforces its diversity and functional resilience as it integrates into a lifelong stable state.

### Future perspectives

As we move beyond mapping the taxonomic trajectory of infant gut microbiota, the next frontier lies in deciphering the causal mechanisms governing host–microbe co‐adaptation. While the sequential transition from aerobic pioneers to anaerobic guilds is well‐documented, future research must pivot toward multi‐omics integration to define the functional output of these communities. Specifically, elucidating how transient metabolic signals, such as the specific kinetics of HMO degradation and menaquinone biosynthesis, program the naïve immune system and neurodevelopmental setpoints during critical windows is paramount. We need to move from identifying “dysbiosis” to pinpointing specific loss of function in metabolic pathways (e.g., tryptophan or SCFA metabolism) that predisposes infants to atopic and metabolic diseases.

Therapeutically, the field is poised to shift from generic probiotic supplementation to precision microbiome engineering. Given the profound impact of early‐life perturbations such as antibiotics and cesarean sections, there is an urgent need for “ecological rescue” strategies. This involves developing next‐generation synbiotics or defined microbial consortia (live biotherapeutic products) that mimic the functional redundancy of the native ecosystem, specifically designed to restore colonization resistance and immune training in compromised infants. Furthermore, understanding the “social microbiome,” that is, how horizontal transmission from siblings and nursery environments shapes diversity, will inform environmental design and public health guidelines to combat the “missing microbes” hypothesis associated with modern sanitation.

Ultimately, viewing the infant microbiome as a plastic, developing organ offers a transformative opportunity. By establishing robust predictive models that integrate diet, environmental exposure, and host genetics, we can aspire to personalized early‐life interventions. These strategies will aim not merely to treat disease, but to optimize the initial microbial assembly, thereby securing long‐term resilience and health across the lifespan.

Once the successional assembly reaches a state of stability at the end of childhood, the microbiota transitions from a structural assembly phase into a lifelong functional regulatory phase. The microbial consortium established during these critical early windows does not exist as a passive collection of taxa; instead, it acts as a persistent physiological imprinting force that recalibrates the host's systemic environment. In adulthood, the research focus shifts from the factors shaping the microbiota to the mechanisms by which this stabilized community functions as a central hub, actively governing the homeostasis of the host's immune, neural, and metabolic axes. This narrative shift from ecological construction to systemic governance integrates disparate developmental stages into a unified model of lifelong host–microbial interaction.

### Microbiota and neurodevelopment

**FIGURE 6:**
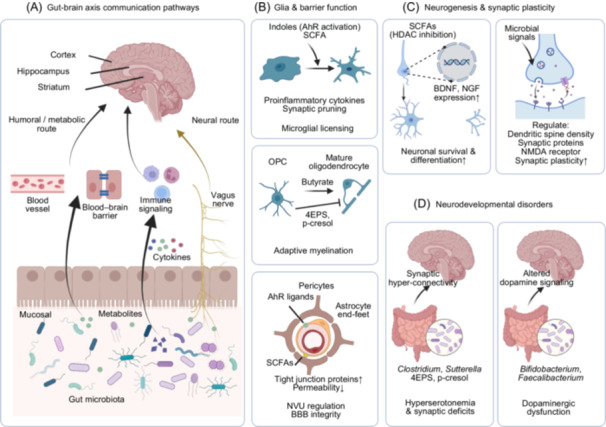
The gut–brain axis: microbial regulation of neurodevelopment and pathogenesis of neurodevelopmental disorders. (A) Gut–brain axis communication pathways. The gut microbiota communicates with the central nervous system (CNS) through three primary interdependent channels: the humoral/metabolic route (microbial metabolites traversing the bloodstream to cross the blood–brain barrier), immune signaling (modulation of systemic cytokines), and the neural route (direct physical signal transmission via the vagus nerve). These pathways converge to modulate structural and functional development in key brain regions, including the cortex, hippocampus, and striatum. (B) Glia and barrier function. Microbial metabolites act as critical environmental cues for glial licensing and barrier integrity. Indoles (via aryl hydrocarbon receptor (AhR) activation) and short‐chain fatty acids (SCFAs) drive microglial maturation, suppressing pro‐inflammatory cytokine production and enabling physiological synaptic pruning. During adaptive myelination, the SCFA butyrate promotes the differentiation of oligodendrocyte precursor cells (OPCs) into mature oligodendrocytes, whereas dysbiosis‐associated metabolites like 4‐ethylphenyl sulfate (4EPS) and p‐cresol inhibit this maturation. Concurrently, SCFAs and AhR ligands fortify the neurovascular unit (NVU) and blood–brain barrier (BBB) by upregulating tight junction proteins across brain endothelial cells, pericytes, and astrocyte end‐feet, thereby decreasing barrier permeability. (C) Neurogenesis and synaptic plasticity. Microbial SCFAs act as endogenous histone deacetylase (HDAC) inhibitors in neuronal cells, maintaining an open chromatin state that drives the transcription of brain‐derived neurotrophic factor (BDNF) and nerve growth factor (NGF). This epigenetic licensing promotes neuronal survival and differentiation. Furthermore, continuous microbial signals are required to regulate synaptic plasticity by optimizing dendritic spine density, NMDA receptor expression, and overall synaptic protein machinery. (D) Neurodevelopmental disorders. Early‐life dysbiosis can derail these regulatory processes, precipitating distinct clinical phenotypes. In Autism Spectrum Disorder (ASD), an overgrowth of specific taxa (e.g., *Clostridium*, *Sutterella*) and the accumulation of neurotoxins (4EPS, p‐cresol) trigger hyperserotonemia and synaptic hyper‐connectivity (driven by defective microglial pruning). In attention‐deficit/hyperactivity disorder (ADHD), functional dysbiosis, characterized by developmental fluctuations in *Bifidobacterium* and the depletion of protective butyrate‐producers like *Faecalibacterium*, is mechanistically linked to dopaminergic dysfunction and altered dopamine signaling circuits.

While the CNS blueprint is genomically encoded, its structural realization is functionally calibrated by the gut microbiome, transforming the developing brain into an adaptively open system. This “hologenomic” integration ensures that critical milestones, from neurogenesis to synaptic pruning, are metabolically attuned to environmental signals. Here, we dissect the specific molecular interplay between commensal microbes and four fundamental CNS cellular compartments, transitioning from correlational phenomenology to causal mechanisms. Furthermore, we delineate how these interactions underpin the pathogenesis of neurodevelopmental disorders (Figure 6).

### Microbial regulation of neurodevelopment

#### Neurogenesis and synaptic plasticity

The orchestration of neurogenesis and synaptic refinement constitutes the fundamental architecture of the developing brain. Emerging evidence identifies the gut microbiota as a critical extrinsic calibrator of these processes. The impact begins at the earliest stages of neural fate determination. Colonizing GF mice with microbiota from low‐growth preterm infants suppresses mature neuronal markers in the offspring's cerebral cortex, indicating impaired early‐life neurogenesis [358]. Even in the adult brain, where neurogenesis persists in the subgranular zone of the dentate gyrus, the microbiome remains a key regulator. Treatment of adult mice with broad‐spectrum antibiotics for 7 weeks arrests hippocampal neurogenesis [359]. This arrest is reversible; reconstitution with specific probiotic strains can reactivate the neurogenic niche [359]. This reactivation suggests a dynamic signaling axis.

Beyond the birth of neurons, the microbiota regulates synaptic plasticity. The striatum of GF models exhibits altered expression of critical synaptic machinery, including synaptophysin and postsynaptic density protein 95 (PSD95) [360]. Moreover, GF mice display reduced expression of the N‐methyl‐d‐aspartate (NMDA) receptor subunit 2B (NR2B), which is essential for long‐term potentiation [361]. Dendritic spine morphology is also highly sensitive to microbial status [362]. This sensitivity indicates that microbial signals are necessary for synapse stabilization and pruning.

A unifying mechanism underlying these defects is the regulation of neurotrophic factors. Microbial absence leads to a notable downregulation of brain‐derived neurotrophic factor (BDNF) in the hippocampus and cortex [360]. This absence creates a “trophic deficit.” Microbial metabolites, particularly SCFAs (butyrate, propionate, and acetate), cross the blood–brain barrier (BBB) and function as endogenous histone deacetylase (HDAC) inhibitors [363]. By preventing histone deacetylation at neurotrophic gene promoters, SCFAs maintain an open chromatin state, promoting BDNF and nerve growth factor (NGF) transcription during critical developmental windows [363]. Thus, microbial metabolites serve as essential epigenetic licenses for neuronal survival and plasticity.

Beyond SCFAs, the repertoire of neuroactive metabolites is vast. Microbiota‐transformed secondary BAs (e.g., tauroursodeoxycholic acid) and microbially synthesized vitamins (e.g., folic acid) regulate mitochondrial function and Notch signaling, both of which are energetic and signaling prerequisites for neural stem cell proliferation [364]. Moreover, the tryptophan–5‐HT axis represents a direct neurochemical link. 5‐HT acts as a trophic factor during early brain development, guiding neurogenesis and circuitry formation [365]. The gut microbiota regulates systemic tryptophan availability and stimulates 5‐HT synthesis. Consequently, dysbiosis can lead to fluctuating central 5‐HT levels. This fluctuation impacts the serotonergic shaping of neuronal circuits [366, 367]. Finally, this communication is not solely humoral. The vagus nerve acts as a physical conduit, transmitting microbial signals to the nucleus tractus solitarius to modulate neuroplasticity and behavior [368]. Together, these findings establish the microbiota as a master regulator of the brain's structural and functional reserve.

#### Microglial ontogeny and functional licensing

Microglia are indispensable architects of neurogenesis and synaptic refinement. Their transition from a primitive embryonic state to a mature surveillance phenotype is continuously calibrated by signals from the gut microbiota [369]. In GF mice, microglia exhibit “maturation arrest” [370]. Morphologically, they fail to achieve the typical ramified state, instead retaining a hyper‐ramified appearance with longer and more complex processes [370]. At the transcriptomic level, deep sequencing reveals the profound downregulation of immune signaling genes. Furthermore, it highlights a failure to downregulate developmental markers (e.g., *Csf1r*) [370]. This immature profile renders the CNS innate immune system hypo‐responsive to acute challenges [370].

The mechanistic bridge linking gut microbiota to microglial maturation is established by microbial metabolites. Mirroring the epigenetic regulation of neurotrophins, SCFAs regulate microglial homeostasis as HDAC inhibitors, fostering an anti‐inflammatory transcriptomic profile [371]. However, their influence extends beyond epigenetics to cellular bioenergetics. Acetate readily traverses the BBB to fuel microglial mitochondrial function [372]. Microglia in GF mice display severe bioenergetic dysfunction, characterized by defective mitochondrial Complex II activity and disrupted membrane potential. These defects are rescued by systemic acetate administration alone [372]. Therefore, the functional fitness of the brain's immune cells is chemically coupled to gut fermentation activity.

Beyond SCFAs, microbial tryptophan metabolites (indole derivatives) serve as ligands for the cytosolic AhR. AhR activation in microglia suppresses NF‐κB‐mediated transcription of neurotoxic cytokines (TNF‐α, IL‐6, IL‐1β) [373, 374, 375]. In gut dysbiosis, the depletion of indole‐producing bacteria precipitates aberrant microglial activation. This disruption impairs synaptic plasticity and compromises neuronal health [373, 374, 375]. Conversely, dysbiosis can lead to the accumulation of neurotoxic *p*‐cresol, a bacterial product of tyrosine metabolism linked to a pro‐inflammatory state [376].

The most functionally significant role of microbiome‐licensed microglia is synaptic pruning via the complement cascade (C1q, C3) [377]. However, the directionality is context‐dependent. In GF animals, maturation arrest leads to insufficient pruning and hyper‐connectivity [370]. In contrast, stress‐induced dysbiosis triggers pathological over‐activation of complement C3, causing aberrant pruning via the C3–complement receptor 3 (CR3) axis [378]. Thus, dysbiosis can drive neurobehavioral pathology through either unchecked phagocytosis or functional dormancy. This highlights the microbiome's role as a critical calibrator of synaptic density. Finally, the influence of microbiota on microglia is highly dependent on sex and timing. Male microglia are more affected by the maternal microbiome prenatally, while female microglia show greater sensitivity to microbial absence in adulthood [379]. This sexual dimorphism provides a biological basis for the higher prevalence of male‐biased neurodevelopmental disorders, such as autism spectrum disorder (ASD) and attention‐deficit/hyperactivity disorder (ADHD) [380].

#### Oligodendrogenesis and adaptive myelination

Myelination is a dynamic form of neural plasticity that is highly sensitive to environmental cues. This process is remarkably protracted, extending from late gestation into the fourth decade of life [381, 382]. This extended timeline renders oligodendrocyte lineage cells uniquely vulnerable to microbial signals. Foundational insights come from GF models. Contrary to the hypoplastic phenotypes often observed in other domains, Hoban et al. reported hypermyelination in the prefrontal cortex (PFC) of GF mice, accompanied by the upregulation of myelin‐related genes (*Mog*, *Mag*, and *Plp1*) [383]. While transcriptional changes normalized after post‐weaning colonization, hypermyelination at the protein level persisted into adulthood [383]. This defines a “critical window” where microbial signals act as a necessary “brake” on structural solidification. This regulatory logic appears evolutionarily conserved, as GF zebrafish models parallel these findings [384]. Corroborating these results, neonatal antibiotic exposure recapitulates this dysregulation [385]. However, this effect is regionally heterogeneous: while the PFC exhibits hypermyelination [383], the hippocampus and internal capsule show reduced myelin density in GF models [386]. This contrast suggests the presence of distinct local signaling milieus.

The functional consequences of this microbe–myelin crosstalk are metabolite‐specific. In stress‐susceptibility models, dysbiotic taxa (e.g., Clostridiales) were associated with elevated *p*‐cresol. This metabolite directly inhibits oligodendrocyte differentiation in vitro [387]. Similarly, 4‐ethylphenyl sulfate (4EPS), derived from microbial tyrosine fermentation and host sulfation, arrests oligodendrocyte maturation and reduces oligodendrocyte–neuron interactions [388]. Systemic 4EPS exposure induces anxiety‐like behaviors in mice [388]. This provides a mechanistic link between gut‐derived toxicity and white matter abnormalities in neurodevelopmental disorders. In stark contrast, SCFAs (particularly butyrate) act as positive regulators. Butyrate acts as an HDAC inhibitor to remodel the chromatin landscape of oligodendrocytes, promoting their maturation. Strikingly, neonatal antibiotic‐induced myelin defects can be rescued by butyrate supplementation [385].

Finally, the microbiota influences myelination indirectly through the neuroimmune environment. Microglia produce pro‐myelinating factors such as insulin‐like growth factor 1 (IGF‐1) [389]. In dysbiotic states, the loss of homeostatic microglial support impairs myelination. In vivo imaging reveals that the microbiota orchestrates microglial recruitment to white matter tracts; without microbial cues, microglia show reduced physical contact with oligodendrocytes [384]. Additionally, SCFAs modulate astrocyte–oligodendrocyte crosstalk by suppressing astrocytic pro‐inflammatory activation via the serum/glucocorticoid‐regulated kinase 1 (SGK1)–IL‐6 pathway, protecting oligodendrocyte precursors [390].

#### Neurovascular unit and blood–brain barrier integrity

The establishment of the BBB represents a critical developmental milestone. This barrier is not a static endothelial wall but a dynamic interface maintained by the neurovascular unit (NVU), which comprises brain endothelial cells, pericytes, and astrocytic end‐feet [391]. The gut microbiota is an essential “architect” of this barrier, dictating its permeability from gestation through adulthood [392]. GF mice exhibit extensive BBB permeability stemming from reduced expression of the tight junction proteins claudin‐5 and occludin [392]. Crucially, this defect is reversible; monocolonization of GF adult mice with SCFA‐producing strains (e.g., *Clostridium tyrobutyricum*) restores BBB integrity [392], highlighting the remarkable plasticity of the NVU in response to microbial cues.

At the molecular level, this gut‐to‐brain enforcement is mediated largely by microbial metabolites acting as epigenetic regulators. Butyrate acts on brain endothelium as an HDAC inhibitor. Braniste et al. showed that butyrate treatment in GF mice restored BBB tightness, linked to increased histone acetylation in brain tissue [392]. Furthermore, physiological levels of TMAO, a metabolite derived from dietary choline and carnitine by gut bacteria, reinforce BBB integrity through annexin A1 signaling [368, 393]. However, elevated TMAO paradoxically compromises barrier integrity [393]. This indicates that homeostatic metabolite levels are required for optimal barrier maintenance.

This microbial influence extends beyond the endothelium to astrocytes and microglia within the NVU. Gut *Lactobacillus* species metabolize tryptophan into indoles and other AhR agonists [394]. Circulating indoles activate AhR signaling in astrocytes and microglia, upregulating neuroprotective factors such as suppressor of cytokine signaling 2 (SOCS2) and suppressing pathogenic pro‐inflammatory programs (e.g., NF‐κB) [395, 396]. By limiting inflammation within the NVU, this pathway prevents secondary BBB breakdown. Thus, the microbiota orchestrates a multi‐layered defense: it physically cements endothelial junctions via SCFAs while simultaneously pacifying the glial environment via AhR agonists.

### Landscape of neurodevelopmental and neuropsychiatric disorders

#### Early‐life metabolic programming of neurodevelopment

Before distinct clinical phenotypes such as ASD or ADHD crystallize, the gut microbiota exerts a foundational influence on cognitive ontogeny. This early‐life window represents a period of “metabolic programming” where microbial failure manifests as global shifts in developmental trajectories. The developing brain sequesters ~50%–60% of resting metabolic energy at birth, rising to ~66% in early childhood [397]. This extraordinary demand necessitates an obligate partnership with the gut microbiota, which provides SCFAs, amino acid derivatives, and vitamins to fuel neuronal growth [368]. Disruption of this supply chain imposes a metabolic penalty on the developing CNS.

Early‐life cohort studies identified a paradoxical relationship between microbial diversity and cognitive performance. While high alpha‐diversity is healthy in adults, higher diversity at 1 year correlates with lower cognitive scores at age two [398]. This suggests that functional specialization is prioritized over ecological complexity during infancy. Indeed, a *Bacteroides*‐dominant microbiome in late infancy associates with superior cognitive outcomes, particularly in males [398, 399]. This signature may facilitate enriched sphingolipid metabolism critical for myelination and efficient breakdown of HMOs into brain‐accessible energy substrates [400]. Crucially, the origins of these metabolic drivers extend back to the maternal environment. Recent birth‐cohort frameworks and multi‐omics studies have begun to connect maternal metabolic states with offspring microbiome and neurodevelopmental phenotypes [401, 402, 403]. For example, maternal gestational diabetes mellitus has been associated with sex‐specific neonatal gut dysbiosis and head circumference abnormalities [402]. Expanding the exposome framework, maternal prenatal co‐exposure to air pollution and psychological distress alters the meconium microbiota, with depletion of *Ruminococcus* mediating adverse neurodevelopmental effects [404]. Complementing this, maternal quinolinate, a tryptophan metabolite critically regulated by host–microbe interactions, has been identified as a key mediator of childhood neurodevelopmental risk [403].

Parallel to these maternal metabolic imprints, longitudinal profiling of the infant microbiome has established independent predictive power. The All Babies in Southeast Sweden (ABIS) study, involving over 16,000 children followed for two decades, identified that distinct microbial and metabolic “scars” in the infant gut precede the clinical diagnosis of neurodevelopmental disorders by years. Compounded by early‐life stressors, infections, and antibiotics, these scars precede conditions ranging from intellectual disability and speech disorders to ADHD and ASD [405]. Specifically, perturbations in the cord serum metabolome and infant lipidome, coupled with the depletion of *Bifidobacterium* and the overgrowth of pro‐inflammatory taxa, serve as sentinel biomarkers for later developmental impairment [405]. These findings imply that what is often clinically categorized as “global developmental delay” may fundamentally represent the initial, non‐specific manifestation of metabolic programming failure. Depending on genetic susceptibility and specific triggers, these systemic deficits eventually resolve into distinct clinical syndromes. In the following sections, we examine how these systemic failures crystallize into the specific pathologies of ASD and ADHD.

#### Autism spectrum disorder

ASD is characterized by deficits in social communication, repetitive behaviors, and restricted interests [406, 407]. While genetic heritability is substantial, incomplete monozygotic concordance underscores the role of environmental modifiers. Recent evidence positions the gut microbiota as a critical interface that interacts with genetic susceptibility to modulate neurodevelopmental trajectories [408, 409, 410]. This is supported by the high prevalence of gastrointestinal (GI) comorbidities in ASD [409, 411, 412, 413]. Studies consistently report distinct dysbiotic signatures in ASD, typically marked by a reduced abundance of beneficial fermenters (*Prevotella*, *Coprococcus*, *Bifidobacterium*) and an overgrowth of potential pathogens (*Clostridium*, *Sutterella*) [414, 415]. However, taxonomic shifts alone may overlook deeper maladaptation. Recent multi‐omics analyses reveal that ASD‐associated dysbiosis also encompasses extensive microbial genomic variation. Chen et al. identified significant single‐nucleotide variants even in species with stable abundance, implying that strain‐level genetic diversity represents a “hidden” dimension of the gut–brain axis [416].

This multi‐layered dysbiosis creates a “double‐hit” metabolic imbalance. First, SCFA deficits compromise BDNF regulation and BBB integrity. In the BTBR mouse model of autism, butyrate administration improved social behavior and restored excitation–inhibition balance [417]. The second “hit” involves the accumulation of neurotoxic byproducts. Elevated p‐cresol is detected in the urine of ASD children and correlates with symptom severity [418]. Similarly, 4EPS, noted for its detrimental impact on oligodendrocytes, shows a striking association with ASD. It is elevated 46‐fold in the maternal immune activation mouse model of autism and 6.9‐fold in ASD children, with systemic 4EPS administration sufficient to induce ASD‐related behavior in mice [419, 420]. Expanding this inventory, Park et al. demonstrated that an elevated glutamate–GABA ratio and 3‐hydroxyglutaric acid promote neuroinflammation by modulating brain‐resident CD4 + T cells [421]. This identifies a cellular mechanism by which gut‐derived metabolic cues drive CNS immune activation.

Structurally, these metabolic and inflammatory insults converge to derail microglial programming. While acute stress triggers aberrant pruning [378], chronic metabolic dysbiosis in ASD induces a state reminiscent of “maturation arrest” manifesting as hyper‐connectivity resulting from pruning deficits. The microbiota guides microglial functional maturation [370]. Consequently, this dysbiosis leads to functional derailment, wherein inflammatory signaling interferes with physiological pruning. This underpins structural abnormalities in post‐mortem ASD brains: excess neurons in the prefrontal cortex and increased dendritic spine densities in the temporal lobe [422, 423]. Thus, the “under‐pruning” hypothesis provides a mechanistic link between the immune‐dysregulating dysbiotic gut and circuit‐level defects in autism.

Beyond metabolic and immune mediators, the microbiota shapes the neurochemical landscape. Spore‐forming bacteria (predominantly Clostridiales) stimulate colonic enterochromaffin cells to produce 5‐HT by upregulating tryptophan hydroxylase 1 (*Tph1*) expression [366]. Given that hyperserotonemia is a robust ASD biomarker and 5‐HT modulates both intestinal motility and socio‐affective behaviors [424], this serotonergic pathway links gut dysbiosis to both GI and behavioral comorbidities [424, 425]. Furthermore, the microbiome regulates social behaviors via the stress response circuitry. Sudo et al. showed that GF mice exhibit an exaggerated stress response reversible only by early‐life colonization [426]. Building on this, Wu et al. revealed that *Enterococcus faecalis* promotes social activity and reduces corticosterone by suppressing corticotrophin‐releasing hormone (CRH) neurons in the paraventricular nucleus [427]. This suggests that specific commensals can act as social rheostats.

Functional causality was demonstrated by fecal microbiota transplantation (FMT) from human ASD donors to GF mice, which transferred autistic behaviors [428]. However, the field is currently grappling with the “diet‐as‐confounder” hypothesis, which suggests that microbiome differences are secondary consequences of restrictive dietary preferences in ASD [429]. Nonetheless, intervention studies support a causal component. In ASD children, microbiota transfer therapy (MTT) yielded durable improvements in both GI and behavioral symptoms, linked to the successful engraftment of *Prevotella* and *Bifidobacterium* [430, 431]. Moving toward precision medicine, specific strains targeting defined neural deficits show promise. *Lactobacillus reuteri* rescues social deficits by upregulating oxytocin via the vagus nerve [432]. Additionally, *Bifidobacterium longum* attenuates neuroinflammation via the kynurenine pathway in autistic rats [433]. Collectively, these findings underscore that the targeted restoration of the gut ecosystem represents a viable strategy for symptom management.

#### Attention‐deficit/hyperactivity disorder

ADHD is defined by inattention, impulsivity, and hyperactivity, often persisting into adulthood [434]. Biologically, the disorder is anchored in the dysfunction of catecholaminergic signaling, specifically dopaminergic dysregulation in the striatum and cortex [435]. Emerging evidence points to a disruption in gut–brain modulation of this system.

The mechanistic basis for a gut–brain–dopamine axis is established in GF models. Diaz Heijtz et al. demonstrated that the gut microbiome functions as a developmental brake on striatal dopaminergic signaling. GF mice have increased striatal dopamine turnover and hyperactivity, mirroring core ADHD symptoms [360]. Tengeler et al. further showed that FMT from human ADHD donors to GF mice compromises brain structural integrity and induces anxiety‐like behaviors [436]. Although this model primarily recapitulated emotional and structural deficits rather than hyperactive‐impulsive behaviors, it provides proof‐of‐concept that the dysbiotic ecosystem alone carries the biological information sufficient to drive ADHD‐relevant neurodevelopmental alterations [436].

In human cohorts, the microbial signature of ADHD points to functional dysbiosis rather than simple ecological collapse. While early studies reported reduced α‐diversity [437], later human studies reported inconsistent or non‐uniform diversity changes and taxa‐level alterations [438, 439]. Instead, specific metabolic pathways appear compromised. A consistent finding is the reduced abundance of *Faecalibacterium prausnitzii*, a key butyrate producer essential for immune homeostasis [440, 441]. Refining this metabolic perspective, the depletion of *Lactobacillus sanfranciscensis* drives core symptoms via imidazoleacetic acid deficiency [442]. Furthermore, *Bacteroides* species may be protective; *B. cellulosilyticus* correlates negatively with impulsivity and inattention, while *B. ovatus* links to attention deficits [438]. This functional dysbiosis likely extends to host metabolic status, as ADHD is frequently comorbid with obesity and micronutrient deficiencies (zinc, magnesium) [443, 444]. This suggests a failure in microbially mediated nutrient absorption.

An intriguing paradox in the ADHD microbiome involves *Bifidobacterium*. This taxon is involved in phenylalanine synthesis, a dopamine precursor. While some studies report elevated *Bifidobacterium* in ADHD patients [440, 441], longitudinal data offer a developmental counterpoint. Early‐life supplementation with *Lactobacillus rhamnosus* GG reduced later ADHD risk. Furthermore, those who developed neuropsychiatric symptoms had reduced *Bifidobacterium* abundance during infancy [445]. This suggests a dynamic temporal trajectory: early‐life *Bifidobacterium* deficits may predispose the developing brain to the disorder, whereas elevated levels observed later might represent a compensatory metabolic response to established dopaminergic dysfunction.

Finally, therapeutic interventions reveal age‐dependent mechanisms. A randomized controlled trial using Synbiotic 2000 showed that in children, the treatment increased propionic acid (an SCFA) and reduced pro‐inflammatory markers [446]. Conversely, in adults, while SCFA levels remained stable, the intervention improved emotion regulation, particularly in those with elevated baseline vascular inflammation [447]. This dichotomy highlights that the gut–brain axis in ADHD is not static; it evolves from a metabolic driver of development in childhood to an immunoregulatory modulator of affect in adulthood.

### Limitations and future perspectives

In summary, the gut microbiota is a multi‐layered calibrator of brain development, operating through four interconnected levels: (1) providing essential metabolites that epigenetically license neurogenesis and myelination; (2) programming microglial maturation and synaptic pruning; (3) maintaining BBB integrity; and (4) establishing the metabolic foundation for cognitive ontogeny. Dysbiosis disrupts these processes, which is strongly associated with distinct neurodevelopmental disorders such as ASD and ADHD. Specifically, ASD is characterized by microglial maturation arrest and synaptic under‐pruning, whereas ADHD is linked to dopaminergic dysregulation. This suggests a shared vulnerability rooted in early‐life microbial programming.

Despite mechanistic clarity from gnotobiotic models, a translational chasm persists between murine causality and human correlation. Most human associations remain observational and confounded by reverse causality, particularly dietary patterns. In ASD research, restrictive eating may drive dysbiosis rather than result from it [429]. Equally critical is the frequent neglect of sexual dimorphism. Despite evidence that microglia and metabolic programming exhibit sex‐specific trajectories [379, 398, 399, 401, 402], many clinical cohorts lack the power to stratify by sex. This limitation potentially masks distinct microbial drivers of male‐biased disorders such as ASD and ADHD. Moreover, reliance on 16S rRNA profiling is insufficient; hidden genomic structural variants in ASD‐associated microbes [416] suggest that strain‐level functional differences matter. Thus, dysbiosis must be redefined at the level of microbial gene expression and metabolite production rather than simple abundance. Finally, while broad metabolic pathways (e.g., SCFAs, tryptophan) are mapped, the pharmacokinetics of how metabolites traverse specific BBB regions to target discrete neural circuits in the human brain remains unquantified. These limitations provide new directions for future research.

The future lies in shifting from descriptive ecology to mechanism‐based precision interventions. We must leverage integrative multi‐omics to identify microbial biosynthetic gene clusters responsible for neuroactive metabolites, enabling next‐generation live biotherapeutic products with defined pharmacological targets rather than generic probiotics. Additionally, we must expand the therapeutic horizon beyond early childhood. Given that myelination persists into adulthood [448] and synaptic plasticity remains dynamic in the adult brain [449], we need to explore whether microbial interventions can reopen “critical periods” in the adult brain to reverse established deficits. Ultimately, the preventative strategy requires longitudinal birth cohorts that layer deep neural phenotyping with the “exposome” (e.g., diet, stress, and pollution). This approach enables us to move from managing symptoms to engineering a resilient gut–brain ecosystem that prevents neurodevelopmental disease at its biological origin. This naturally directs our attention to the foundational role of early microbial colonization in educating the host's systemic immunity, a critical developmental prerequisite systematically explored in the following section on Microbiota and immune development.

### Microbiota and immune development

**FIGURE 7:**
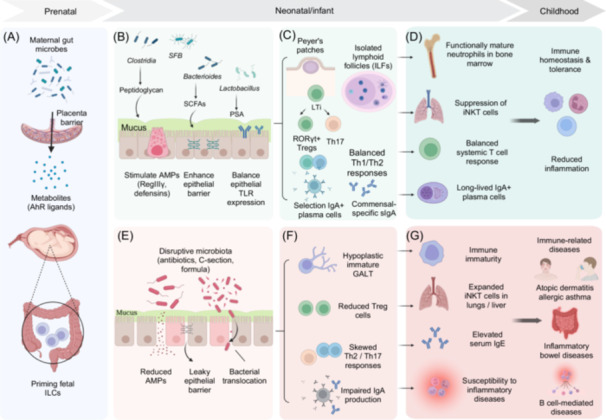
Microbial orchestration of early‐life immune development and the long‐term consequences of developmental dysbiosis. (A) Maternal gut microbes produce metabolites, such as aryl hydrocarbon receptor (AhR) ligands, which cross the placental barrier to prime fetal innate lymphoid cells (ILCs), preparing the neonatal immune system for postnatal challenges. (B) Commensal taxa such as *Clostridia*, *Bacteroides*, and *Lactobacillus* produce short‐chain fatty acids (SCFAs) and polysaccharide A (PSA) that stimulate the production of antimicrobial peptides (AMPs, e.g., RegIIIγ, defensins), enhance epithelial barrier integrity, and balance Toll‐like receptor (TLR) expression. (C) Direct microbial engagement, specifically the sensing of bacterial peptidoglycan, drives the postnatal formation of isolated lymphoid follicles (ILFs) and regulates the differentiation of retinoic acid receptor‐related orphan receptor gamma (RORγ)+ regulatory T cells (Tregs) and Th17 cells, fostering balanced systemic responses. (D) Synchronized microbial education promotes the development of mature bone marrow neutrophils, suppresses pro‐inflammatory invariant natural killer T (iNKT) cells in pulmonary tissues, maintains balanced Th1/Th2 responses, and establishes a reservoir of long‐lived IgA+ plasma cells, collectively ensuring systemic immune tolerance. (E) External pressures such as perinatal antibiotics, C‐section delivery, and formula feeding deplete beneficial commensals and reduce AMP secretion, leading to a porous, “leaky” epithelial barrier and pathological bacterial translocation. (F) This dysbiosis results in hypoplastic/immature gut‐associated lymphoid tissue (GALT), a reduction in protective Treg cells, skewed Th2/Th17 inflammatory responses, and impaired mucosal IgA production. (G) Developmental immune immaturity and failure to establish tolerance predispose the host to a spectrum of immune‐related pathologies, including atopic dermatitis, allergic asthma, inflammatory bowel diseases (IBD), and various B cell‐mediated autoimmune conditions.

The establishment of the early‐life microbiota constitutes a decisive biological event that directs the postnatal development and functional calibration of the host immune system [146, 450, 451, 452]. Early‐life immunity is characterized not as an incomplete version of the adult system, but as an alternatively programmed state marked by generally suppressed inflammatory responses [453, 454]. While this adaptation may increase susceptibility to infection and limit robust responses to novel antigenic challenges, it simultaneously underscores the essential need for incorporating precise environmental instruction [453, 455, 456, 457]. Disruption of this foundational programming directly compromises immune physiology and underlies the pathogenesis of a spectrum of inflammatory, allergic, and autoimmune diseases [304, 450, 458, 459] (Figure 7).

### Impacts of the microbiota on lymphoid tissue maturation

Microbial signals are integral to the structural morphogenesis of the intestinal mucosa. While the initial formation of Peyer's patches is driven by autonomous embryonic programs, their postnatal architecture relies heavily on microbial presence [460, 461]. Conversely, isolated lymphoid follicles (ILFs) arise entirely de novo during the postnatal period. This process is strictly microbiota‐dependent and in line with their preferential distribution within the bacteria‐rich distal gut [462]. In GF conditions, this process is arrested at the immature cryptopatch stage. The transition to a functional ILF requires direct microbial engagement, epitomized by the sensing of bacterial peptidoglycan by epithelial nucleotide‐binding oligomerization domain‐containing protein 1 (NOD1) receptors. This signal initiates a chemokine cascade that recruits lymphoid tissue inducer (LTi) cells, a specialized subset of ILC3s [463]. These stromal organizers, via the secretion of lymphotoxin‐α1β2, direct the clustering of B cells and the formation of the follicular architecture, thereby converting genetic potential into anatomical reality [464]. Consequently, the gut‐associated lymphoid tissue (GALT) of GF animals remains profoundly hypoplastic and functionally immature, underscoring a non‐redundant role for commensals [465].

The impact of the early‐life microbiota on systemic lymphoid organs manifests primarily through functional calibration rather than structural genesis [146]. Organs such as the spleen and peripheral lymph nodes develop their basic architecture in GF mice, yet they exhibit a state of functional and microarchitectural underdevelopment [466, 467]. This is characterized by poorly defined marginal zones in the spleen and diminished cellularity in T cell zones [468]. The systemic immune system thus exists in a hypo‐stimulated, “untuned” state, reflecting the absence of continuous, low‐level microbial signaling required to train innate immune responses and prime adaptive repertoires [469, 470].

### Innate immune system

The fundamental interplay between host genetic potential and microbial instruction extends beyond lymphoid organ formation to encompass the functional maturation of the intestinal barrier and the systemic calibration of innate immunity [146]. Guided by a “layered immunity” principle [471], this time‐sensitive process progresses within a series of critical “windows of opportunity” [304], wherein host–microbe interactions sequentially orchestrate distinct developmental phases from gestation to weaning to establish lifelong homeostasis.

The microbiota‐mediated immune education begins *in utero*, mainly through the transfer of microbiota‐derived metabolites across the placenta [472, 473]. Maternal gut commensals produce metabolites, such as AhR ligands, which reach the fetus and directly shape the development of its innate immune system. These ligands stimulate the proliferation of fetal ILC3s, priming them for postnatal function in barrier defense and IL‐22 production [472]. This gestational programming demonstrates that the maternal microbiota serves as a remote regulator, preparing the neonatal immune system for its future microbial coexistence even before birth [473].

Upon birth, this gestational priming pattern is rapidly superseded as the mode of immune instruction transitions from remote microbial signaling to direct, continuous physical engagement with the colonizing microbiota [288]. The neonatal epithelium acts as an active selector of this developing community rather than a passive barrier. For example, the expression of Toll‐like receptor 5 (TLR5) on neonatal enterocytes is 200‐fold higher than in adults, an age‐dependent and microbial‐driven feature that shapes the community composition by selecting against flagellated bacteria, with effects lasting into adulthood [474]. To enforce this selection and maintain spatial segregation, microbial signals drive the maturation of the epithelial antimicrobial defense. Specifically, commensal sensing triggers ILC3s to secrete IL‐22, which induces Paneth cells to produce high concentrations of antimicrobial peptides (AMPs), particularly C‐type lectins, such as regenerating islet‐derived protein 3 gamma (RegIIIγ) and α‐defensins [475, 476]. Notably, Paneth cells can also directly sense microbial signals via MyD88‐dependent Toll‐like receptor activation [477]. These AMPs are essential for maintaining the demilitarized zone at the mucosal surface [478]. Concurrently, microbial signals direct goblet cell maturation, ensuring secretion of a structured inner mucus layer dominated by MUC2 [479]. This mucus layer acts as a physical sieve, segregating commensals from the epithelium [479, 480]. Microbial metabolites, particularly SCFAs, fortify barrier integrity by serving as the primary energy source for colonocytes and upregulating tight junction protein expression [481].

Furthermore, early‐life microbial colonization drives a gain in DNA methylation in intestinal stem cells, particularly affecting genes involved in glycosylation and barrier function. This microbiota‐dependent epigenetic remodeling, most active from birth until weaning, is essential for establishing a resilient barrier and determines lifelong susceptibility to inflammatory challenges [303, 482, 483].

Systemic effects of gut microbial signals include the regulation of steady‐state granulopoiesis in the bone marrow. GF or antibiotic‐treated neonates exhibit reduced circulating and bone marrow neutrophils, as well as defects in granulocyte progenitor cells, rendering the host vulnerable to systemic sepsis [484]. Furthermore, microbial signals are necessary for the functional aging of neutrophils, optimizing their chemotactic response via C‐X‐C motif chemokine receptor 2 (CXCR2) and their oxidative burst capacity [485].

### Adaptive immune system

While the innate immune system establishes the foundational architecture of host defense, the adaptive immune system requires antigenic exposure to achieve functional maturity [146, 450, 486]. The gut microbiota serves as the principal source of such antigens, driving the postnatal maturation of the adaptive immune landscape [487]. This microbial education is critical for shifting the system from a default, neonatal state toward a balanced and diverse network capable of nuanced responses. The profound immunodeficiency observed in GF animals, as marked by underdeveloped GALT and impaired antibody responses, exemplifies this essential microbial role [488]. More than merely promoting structural development, however, the microbiota precisely calibrates the fundamental balance between immune tolerance and active defense, often within critical early‐life windows that determine long‐term immunological trajectories [489].

The most fundamental decision the intestinal immune system must make is to distinguish between commensal partners and pathogenic threats [489]. To maintain mucosal homeostasis, the host develops two functionally opposed yet microbiota‐dependent T cell compartments: pro‐inflammatory Th17 cells essential for barrier defense, and Tregs that enforce tolerance [489]. The regulation of T cell differentiation by the gut microbiota is highly bacterial species‐specific. On one hand, specific commensals, such as Clostridia clusters IV and XIVa, and their metabolite SCFAs, drive the differentiation of symbiotic‐tolerant RORγ+ Tregs, which are functionally distinct from GATA3+ Tregs that respond to tissue damage [490, 491, 492]. In contrast, the Th17 cells essential for barrier defense are specifically induced by entirely different microbes, such as SFB [338], through a separate signaling pathway involving epithelial‐derived serum amyloid A [493].

Within the GALT, the microbiota further fine‐tunes the delicate balance between Th1 and Th2 responses. While the GF intestine is naturally skewed toward Th2, specific microbes are required to induce a counterbalancing Th1 response. For example, ectopic colonization by *Klebsiella* strains can induce Th1 cell proliferation via BATF3‐dependent dendritic cells and IL‐18 signaling [494, 495]. Conversely, the microbiota also restrains excessive Th2 activity; members of the Lachnospiraceae family induce dendritic cells to secrete transforming growth factor beta (TGF‐β), which specifically inhibits Th2 differentiation, thereby preventing allergic‐type inflammation in the local gut environment [496].

This microbial education extends beyond conventional T cells to calibrate innate‐like lymphocytes, including ILCs and mucosa‐associated invariant T (MAIT) cells [497, 498, 499]. MAIT cells rely on specific microbial metabolites such as riboflavin derivatives for their development, a process that is strictly time‐dependent and requires exposure within a specific neonatal window of opportunity [499]. Beyond such direct metabolic dependencies, early microbial exposure exerts a profound influence on the development of the entire immune system. Cesarean section delivery, for instance, bypasses vertical transmission of maternal gut microbes, leading to colonization by skin and hospital‐environment bacteria (e.g., *Staphylococcus*, *Klebsiella*) [162, 163]. This altered pioneer community is associated with an expansion of colonic invariant natural killer T cells, creating a persistent pro‐inflammatory tone that heightens susceptibility to allergic and inflammatory diseases in both mice and humans [500]. Crucially, this accumulation can only be inhibited by colonization during the neonatal period, not in adulthood [500].

Aside from effects within the intestine, the gut microbiota also plays a broader educational role in systemically correcting the skewed T cell immune characteristics of the neonatal period in various organs. For example, Polysaccharide A from *Bacteroides fragilis* actively counters Th2 skewing by promoting Th1 cytokines in the spleen [501]. This systemic calibration underpins the gut–lung axis. Appropriate early‐life microbial colonization epigenetically silences the expression of the chemokine CXCL16 in pulmonary tissue. This suppression is crucial to prevent the pathological accumulation of pro‐inflammatory iNKT cells in the lungs [500]. Disruption of this process, due to dysbiosis from antibiotics or cesarean section delivery, leaves the pulmonary immune system in a hyper‐reactive state, directly linking early gut microbiota composition to the risk of childhood asthma [502, 503].

Parallel to its role in shaping T cell immunity, the microbiota is a central regulator of the B cell arm of adaptive immunity [504, 505, 506]. In the earliest stages of life, however, the infant is functionally immunodeficient and relies almost exclusively on maternal IgA provided via breast milk for mucosal defense [507]. This maternal shield offers passive “immune exclusion” while the infant's own system prepares for independence [508]. Crucially, this protection is not merely a physical barrier. Maternal IgG and IgA antibodies actively dampen mucosal T helper cell responses, thereby preventing excessive immune activation against the colonizing microbiota [509].

As colonization takes place, microbial signals trigger the switch from passive reliance to active production, influencing early B‐lineage development directly within the gut lamina propria and driving the positive selection of the B cell repertoire within the GALT [510]. Microbial symbionts shape the primary immunoglobulin repertoire by selecting for B cells with polyreactive or commensal‐specific V(D)J rearrangements [511]. Functionally, the resulting mucosal IgA performs a critical discriminatory role, preferentially targeting specific bacterial taxa, particularly those with high immunogenic or colitogenic potential [512]. This targeted IgA coating spatially restricts these bacteria, limits their motility, proliferation and epithelial adhesion, and thereby regulates their pathogenicity to maintain homeostasis [513, 514, 515]. Advanced experimental models employing reversible microbial colonization have demonstrated that mucosal IgA production is not merely a transient reaction but a persistent adaptation that endures long after the initial microbial exposure has ceased [516]. These findings align with observations that while the shaping process is highly individualized, the interplay between the specific microbiota composition and T cell help drives the diversification of the IgA repertoire as the host ages [517].

This early‐life developmental process helps establish a self‐sustaining layer of IgA^+^ B cells that persists into adulthood. Unlike the transient antibody responses typical of later exposure, these early‐imprinted B cells integrate into the gut tissue and continuously drive steady‐state sIgA responses [518]. The absence of this early microbial education leads to humoral immune dysregulation not only locally in the gut but also in the systemic immune compartment. GF mice exhibit supraphysiological levels of serum IgE, accompanied by an expansion of basophils and mast cells, which predisposes the host to systemic anaphylaxis [519]. This highlights the strictly different temporal rules. While IgA levels can be boosted by colonization at any age, the suppression of this hyper‐IgE state is restricted to a critical neonatal window [516, 519].

### Clinical consequences of developmental dysbiosis

The so‐called “atopic march,” which describes the progression from atopic dermatitis (eczema) in early infancy to allergic asthma and allergic rhinitis in childhood, provides a paradigmatic example of this escalating failure. This phenomenon traces a path from disrupted skin and gut barriers to systemic allergic sensitization [520, 521]. Infants who later develop allergic disease exhibit early‐life gut microbiota disruption, characterized by delayed maturation and reductions in butyrate‐producing Clostridia and *Bifidobacterium* species [522, 523, 524]. These microbial alterations are linked to impaired immune programming and barrier function, facilitating sensitization to allergens via multiple routes [525, 526]. This early dysfunction contributes to the breakdown of oral tolerance and pulmonary immune homeostasis, consistent with microbiota profiles characteristic of atopic dermatitis, food allergy, asthma, and allergic rhinitis [527, 528].

#### Type 1 diabetes

The loss of microbial guidance predisposes the host to diverse immune‐mediated diseases, from autoimmunity to chronic inflammation [529]. In T1D, the commensal microbiota counteracts dysregulation through both innate signaling and adaptive immune modulation [530, 531]. The protective effect relies on the engagement of innate immune sensors. Furthermore, gut microbial metabolites such as acetate and butyrate limit autoreactive T cell frequency and prevent pancreatic β‐cell destruction [530, 531]. The parallel rise in autoimmune incidence suggests that modern environmental conditions fail to provide the specific microbial education required to establish immune checkpoints [532].

#### Inflammatory bowel diseases

The breakdown of immune tolerance is most direct in inflammatory bowel disease (IBD), including Crohn's disease (CD) and ulcerative colitis (UC), which represents a catastrophic loss of tolerance to the commensal microbiota itself [533]. For example, CD and UC are defined by a characteristic dysbiosis, specifically the depletion of anti‐inflammatory keystone species such as *Faecalibacterium prausnitzii* [534, 535]. Early‐life microbial perturbations, such as repeated antibiotic courses or a lack of breastfeeding, may permanently alter the “set‐point” of the gut mucosal immune system. This misprogramming can make it hyperreactive to even minor fluctuations in the gut microbial composition later in life [536]. This manifests as persistently impaired intestinal barrier function and an overactivation of effector T cells (e.g., Th1, Th17) directed against commensal bacteria, coupled with the relative insufficiency of Tregs [450, 489].

#### B cell‐mediated diseases and vaccination

In the context of B cell differentiation and functional activation, microbes play a nuanced role in both facilitating immune responses and maintaining immune tolerance. A healthy microbiota is requisite for the differentiation of regulatory B cells (Bregs), which maintain homeostasis by producing IL‐10 [506, 537]. Conversely, dysbiosis can unlock pathogenic B cell potential. The translocation of specific pathobionts, such as *Enterococcus gallinarum*, to the liver and systemic tissues has been identified as a driver of systemic lupus erythematosus. This translocation promotes the production of autoantibodies and systemic inflammation, partly through molecular mimicry [538, 539]. Furthermore, this microbial fluctuation is equally critical for intentional immune interventions such as vaccination through dual signaling and metabolic pathways. First, the microbiota can act as an endogenous adjuvant, providing tonic stimulation such as flagellin sensing via TLR5 to prime plasma cell differentiation [540]. Second, gut‐derived SCFAs control gene expression necessary for plasma B cell differentiation [541]. When this signal is disrupted by antibiotics, as observed in human influenza‐vaccination studies, there is impaired H1N1‐specific IgG1/IgA response and enhanced inflammatory signaling [542].

### Perspective and limitations

While mounting evidence demonstrates that neonatal microbiota–immune crosstalk impacts lifelong allergic and autoimmune disease risk, a critical knowledge gap remains. Specifically, there is a lack of long‐term follow‐up data to support whether interventions such as microbiota transplantation in cesarean‐delivered newborns can sustainably reduce the long‐term risk of diseases such as asthma and obesity. Most current investigations of intestinal immune disorders and dysbiosis remain confined to adult cohorts, and longitudinal data tracking the same niche from childhood to adulthood are still scarce. Consequently, we lack quantitative evidence for how an early‐life immune niche insult imprints lifelong disease susceptibility. Existing animal models largely rely on acute models (e.g., dextran sulfate sodium (DSS)‐induced colitis or mono‐association with single bacterial strains), failing to recapitulate lifespan‐scale crosstalk among immune cells and the microbiome [543, 544, 545, 546]. Although single‐cell and spatial multi‐omics can map cellular states, ethical and sampling constraints prohibit repeated biopsies of the same crypt over time [547, 548]. Future efforts must integrate non‐invasive longitudinal sampling with human‐derived organoid–microbiota co‐cultures that can be serially sampled for years. Coupled with artificial intelligence (AI)‐guided multi‐scale modeling, such platforms will clarify how “childhood inflammatory memory” is encoded in stem‐cell epigenomes, mesenchymal scaffolds, and microbial metabolites. Ultimately, this will enable time‐window interventions that target the niche itself rather than simply suppressing immunity.

### Microbiota and metabolic development

**FIGURE 8:**
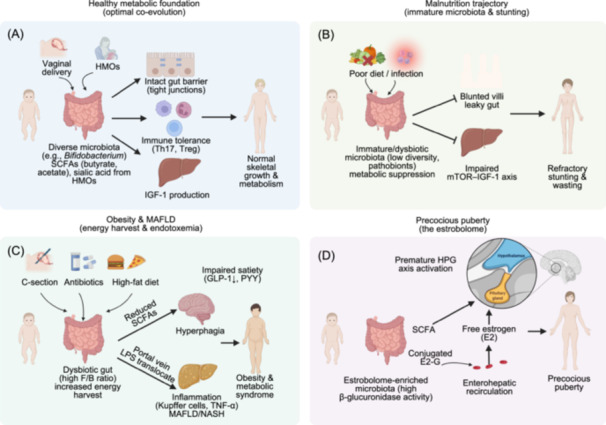
Microbiota‐mediated metabolic programming and the developmental origins of health and disease. (A) Healthy metabolic foundation. In optimal co‐evolutionary settings, vaginal delivery and breastfeeding (providing HMOs) establish a diverse, *Bifidobacterium*‐dominated microbiota. This community produces essential metabolites, including short‐chain fatty acids (SCFAs) like butyrate and acetate, and facilitates the synthesis of sialic acid. These signals promote intestinal barrier integrity, induce immune tolerance (via Th17 and Treg cells), and stimulate hepatic insulin‐like growth factor 1 (IGF‐1) production, collectively supporting normal skeletal growth and metabolic homeostasis. (B) Malnutrition trajectory. Poor diet and early‐life infections drive an immature, dysbiotic microbiota characterized by low diversity and a bloom of pathobionts. This state is marked by blunted intestinal villi, increased gut permeability (leaky gut), and suppressed metabolic signaling (impaired mammalian target of rapamycin (mTOR)/insulin‐like growth factor 1 (IGF‐1) axis), leading to refractory stunting and wasting. (C) Obesity and metabolic dysfunction‐associated fatty liver disease (MAFLD). Perinatal pressures (C‐section, antibiotics, and high‐fat diet) disrupt early succession, resulting in a dysbiotic gut with a high Firmicutes‐to‐Bacteroidetes (F/B) ratio. This configuration enhances energy harvest and increases portal vein lipopolysaccharide (LPS) translocation. Systemic inflammation (via Kupffer cells and TNF‐α) and impaired satiety signaling (reduced glucagon‐like peptide‐1 (GLP‐1)/peptide YY (PYY)) contribute to hyperphagia and the development of MAFLD. (D) Precocious puberty (the estrobolome). An estrobolome‐enriched microbiota, characterized by high bacterial β‐glucuronidase activity, promotes the deconjugation of biliary estrogens (E2‐G) into free estrogen (E2). This increases enterohepatic recirculation, elevates systemic estrogen levels, and may trigger premature hypothalamic–pituitary–gonadal (HPG) axis activation, ultimately predisposing children to precocious puberty.

During early life, the microbiota develops in tandem with host metabolism through molecular signaling; this early crosstalk can shape lifelong metabolic trajectories (metabolic imprinting). Disruptions from delivery mode, antibiotics, and nutrition may impair this process and raise metabolic disease risk from childhood into adulthood. This section summarizes key mechanisms and clinical implications, focusing on early colonization and outcomes including malnutrition, obesity and appetite regulation, metabolic dysfunction‐associated fatty liver disease (MAFLD), physical growth, and precocious puberty (Figure 8).

### Microbiota‐set metabolic development

Cesarean section delays *Bacteroides* colonization and is associated with a 20%–46% increased risk of childhood overweight [549, 550]. Early “microbial imprinting” may alter metabolic gene expression via epigenetic mechanisms; for example, microbiota‐derived SCFAs act as HDAC inhibitors, modifying chromatin at metabolic genes in the gut and liver and setting energy metabolism baselines [551]. Antibiotic exposure early in life disrupts this fragile ecology; antibiotic use within the first 6 months correlates with higher later BMI [552].

During the first 6 months, breast milk provides nutrients and HMOs, a key selective force shaping the infant microbiota. HMOs (indigestible to the infant) enrich *Bifidobacterium* (especially *B. longum* subsp. *infantis*) [553], supporting pathogen exclusion and metabolic cooperation. Charbonneau et al. showed that sialic acid produced from microbiota utilization of SMOs promotes skeletal growth and metabolic maturation in mice [298]. Disruption of the mother–milk–microbiota–host axis (e.g., formula feeding or poor HMO utilization) can blunt villus development, delay barrier maturation, suppress hepatic IGF‐1 axis activity. Furthermore, it can increase systemic inflammation, impair muscle growth and adipose energy storage, and predispose the infant to metabolic syndrome [171, 554].

The early microbiota also supports immune “tolerance training.” Certain microbes (e.g., SFB) induce Th17 differentiation and strengthen mucosal barriers [338]. Without such training, gut permeability increases, enabling LPS translocation and metabolic endotoxemia. LPS activates adipose inflammation via TLR4, promoting adipocyte hyperplasia and early insulin resistance programming [337, 555, 556, 557].

### Gut microbiota and childhood malnutrition

Malnutrition is not only caloric and micronutrient deficiency: many children show “refractory” malnutrition with poor catch‐up growth despite ready‐to‐use therapeutic foods (RUTF). Seminal studies have established microbiota immaturity as a core driver and developed microbiome‐targeted therapies. In a Bangladesh birth cohort, Subramanian et al. modeled microbiota development using age‐discriminatory taxa to compute “microbiota age.” Children with severe acute malnutrition showed marked microbiota immaturity, characterized by depleted adult‐type genera (e.g., Ruminococcaceae, Lachnospiraceae) and enriched potential pathogens (e.g., Vibrionaceae) [558]. RUTF transiently improved weight but did not repair immaturity; upon cessation of treatment, the microbiota often regressed to a developmentally arrested state. The persistent low microbiota‐for‐age Z‐score (MAZ) strongly correlates with long‐term stunting [558].

Causality was supported by FMT studies in GF mice. Using discordant twins (kwashiorkor vs. healthy), Smith et al. transplanted donor microbiota into GF mice: mice receiving the malnourished microbiota gained less weight and lean mass on identical diets [559]. Metabolomics showed host “metabolic suppression.” This was characterized by reduced tricarboxylic acid (TCA) intermediates, impaired gluconeogenesis, and decreased essential amino acid bioavailability (e.g., valine and leucine) [559, 560, 561].

Mechanistically, growth regulation involves specific pathways. Schwarzer et al. found strains such as *Lactobacillus plantarum* stimulate the growth hormone (GH) axis by inducing hepatic and systemic IGF‐1, and the absence of key microbes causes “GH resistance” (normal GH but low IGF‐1) [562]. Additionally, Yan et al. found that gut microbiota‐derived SCFAs promote bone formation [563]. Central to growth is the mammalian target of rapamycin (mTOR) amino acid‐sensing pathway: malnutrition‐associated dysbiosis inhibits ribosomal protein S6 kinase beta‐1 (S6K1) phosphorylation (a downstream effector of mTOR), impairs Paneth cells, and weakens the barrier, reinforcing a “dysbiosis–malabsorption–inflammation–stunting” cycle [562, 564, 565, 566].

These insights explain why calorie‐focused RUTF may fail to restore microbiota function. Gehrig et al. identified glycans (from chickpea, soy, and banana) that selectively promote “growth‐associated taxa” (e.g., *Prevotella copri*, *Faecalibacterium prausnitzii*) [561]. The resulting microbiota‐directed complementary food‐2 (MDCF‐2) improved MAZ in clinical trials and increased growth‐associated plasma proteins including IGF‐1, enabling “precision ecological intervention” for malnutrition [561, 567].

### Childhood metabolic disorders

#### Childhood obesity and appetite regulation

Obesity involves microbiota‐driven regulation via the gut–brain–microbiota axis. Obese children often show a higher Firmicutes‐to‐Bacteroidetes ratio [568], consistent with enhanced dietary energy harvest from otherwise indigestible polysaccharides [569]. Ridaura et al. demonstrated transmissibility: microbiota from obese twins increased adiposity when transplanted into GF mice [570]. Enrichment of *Methanobrevibacter smithii* may further increase fermentation efficiency by consuming hydrogen [571].

Microbial SCFAs (notably propionate and butyrate) signal appetite regulation by activating GPR41 and GPR43 on enteroendocrine l‐cells, inducing glucagon‐like peptide‐1 (GLP‐1) and peptide YY (PYY) secretion [572]. GLP‐1 delays gastric emptying and acts on hypothalamic circuits to reduce appetite. Pediatric studies suggest obese children may have fewer SCFA‐producing bacteria (e.g., *Bifidobacterium*). This potentially blunts postprandial satiety signaling and promotes weight gain [573].

Microbiota also communicates with the brain via the vagus nerve. Propionate can trigger intestinal–brain glucose production through peri‐portal neural mechanisms and suppress feeding centers [574]. Microbes regulate tryptophan metabolism and gut‐derived 5‐HT (≈90% of total). For instance, spore‐formers induce enterochromaffin 5‐HT synthesis [366]. Dysbiosis that diverts tryptophan toward kynurenine may reduce central 5‐HT signaling and contribute to emotional overeating [575]. Microbiota can also modulate ghrelin; *Helicobacter pylori* eradication often increases ghrelin and weight, suggesting gastric microbes may restrain appetite initiation [576].

#### Pediatric metabolic dysfunction‐associated fatty liver disease

The liver is exposed to gut‐derived products via the portal vein, forming the gut–liver axis. The term MAFLD was proposed based on international consensus criteria published in recent years [577]. The renaming from non‐alcoholic fatty liver disease (NAFLD) to MAFLD aims to highlight the association of the disease with metabolic abnormalities, such as obesity, insulin resistance and dyslipidemia, instead of the previous exclusive definition centered on the “non‐alcoholic” attribute. Children with NAFLD/MAFLD may exhibit increased intestinal permeability and higher circulating LPS, which correlate with liver disease severity [578]. This facilitates LPS influx. Kupffer cells sense LPS via TLR4 and release cytokines such as TNF‐α [579], promoting progression from steatosis to non‐alcoholic steatohepatitis (NASH) and fibrosis [580]. Yuan et al. identified high‐alcohol‐producing *Klebsiella pneumoniae* (HiAlc) enriched in some NASH patients; in carbohydrate‐rich contexts, it generates ethanol, increasing endogenous blood ethanol and inducing oxidative and mitochondrial liver damage reminiscent of alcoholic liver disease [581]. In experimental ethanol‐induced liver disease models, engineered bacteria producing AhR agonists have shown hepatoprotective effects, suggesting a broader therapeutic potential for microbiota‐based metabolic modulation [582].

Microbiota also shapes BA metabolism by converting primary to secondary BAs, influencing hepatic synthesis and intestinal signaling [583]. MAFLD is associated with altered BA profiles (e.g., taurine‐conjugated ratios) that may antagonize intestinal FXR signaling, increasing hepatic lipogenesis and reducing fatty acid oxidation [584, 585]. FXR agonists and microbiota modulation to restore bile acid balance are emerging strategies [586]. Another mechanism involves choline: microbial conversion of choline to trimethylamine (TMA) lowers host choline availability; choline deficiency impairs very low‐density lipoprotein export and worsens hepatic triglyceride accumulation [587].

#### Pediatric growth and precocious puberty

With the rising prevalence of precocious puberty, notably idiopathic central precocious puberty (ICPP), the gut microbiota is increasingly viewed as an “invisible endocrine organ.” Cross‐sectional studies report higher α‐diversity in girls with ICPP and enrichment of obesity‐related taxa (e.g., *Ruminococcus*, *Gemmiger*) [588], suggesting links among obesity, microbiota, and pubertal timing. The “estrobolome” comprises gut bacterial genes encoding β‐glucuronidase [589]. Conjugated estrogens excreted into the gut can be deconjugated by β‐glucuronidase, enabling reabsorption via enterohepatic circulation [590]. Dysbiosis may increase this activity, elevating circulating estrogens and potentially promoting early sexual development [591].

Microbial metabolites may also influence the hypothalamic–pituitary–gonadal (HPG) axis. SCFAs can affect neurotransmitter biosynthesis and signaling (e.g., dopamine, 5‐HT) via the gut–brain axis, influencing gonadotropin‐releasing hormone (GnRH) neuron activity [592]. Diet‐induced dysbiosis may alter hypothalamic *Kiss1* expression, increasing GnRH secretion and advancing pubertal onset [593]. Microbiota also modulates IGF‐1, which supports somatic growth and acts as a metabolic gating signal for puberty [594, 595]. This helps explain why overnutrition and dietary patterns that reshape the microbiota are risk factors for precocious puberty.

### Limitations and future perspectives

While significant breakthroughs have been achieved in understanding microbial regulation of pediatric metabolic development, most notably the clinical implementation of MDCF‐2, several critical challenges remain. A primary limitation is that current mechanistic insights are largely derived from animal models or correlational studies. The extent to which inter‐individual heterogeneity and environmental stressors, such as dietary patterns and antibiotic exposure, compromise the long‐term stability of the human microbiota warrants rigorous investigation. Furthermore, the molecular choreography within microbiota–organ axes, particularly the spatiotemporal targeting of key metabolites like SCFAs and BAs during critical developmental windows, remains to be fully elucidated.

Looking forward, the research paradigm must shift from static taxonomic profiling toward dynamic, function‐driven analyses integrated through multi‐omics frameworks. This evolution will likely drive a transition in clinical practice from generic probiotic supplementation to “individualized precision prescriptions” that harmonize host genomics with microbial metabolic phenotypes. Moreover, advancing intervention windows to the prenatal or early postnatal periods represents a strategic frontier for preempting the developmental origins of adult metabolic diseases. As the field moves from characterizing microbial ecology to actively reshaping human health, interdisciplinary synergy will be indispensable in deciphering the metabolic code of the human holobiont.

Notably, microbiota restoration strategies in cesarean‐delivered infants, such as VMT and FMT, have shown partial success in reconstituting early microbial composition. However, the long‐term effects remain uncertain, research conclusions remain controversial, and evidence from long‐term follow‐up is insufficient. Current evidence is largely limited to short‐term observations, and it is unknown whether such compositional recovery can be stably maintained or translated into functional restoration, including microbial metabolite production and immune training. Importantly, there is still a lack of longitudinal clinical evidence demonstrating that these interventions can durably reduce the risk of diseases such as asthma, obesity, or metabolic disorders later in life. Therefore, caution is warranted in interpreting early microbiota normalization as a proxy for long‐term health benefits.

Importantly, while the current section delineates mechanistic links between early‐life microbiota and metabolic outcomes, these processes are inherently time‐dependent. Integrating these findings with the concept of critical developmental windows will be essential to understand how transient early perturbations are translated into persistent physiological trajectories.

### Critical windows and long‐term effects

**FIGURE 9:**
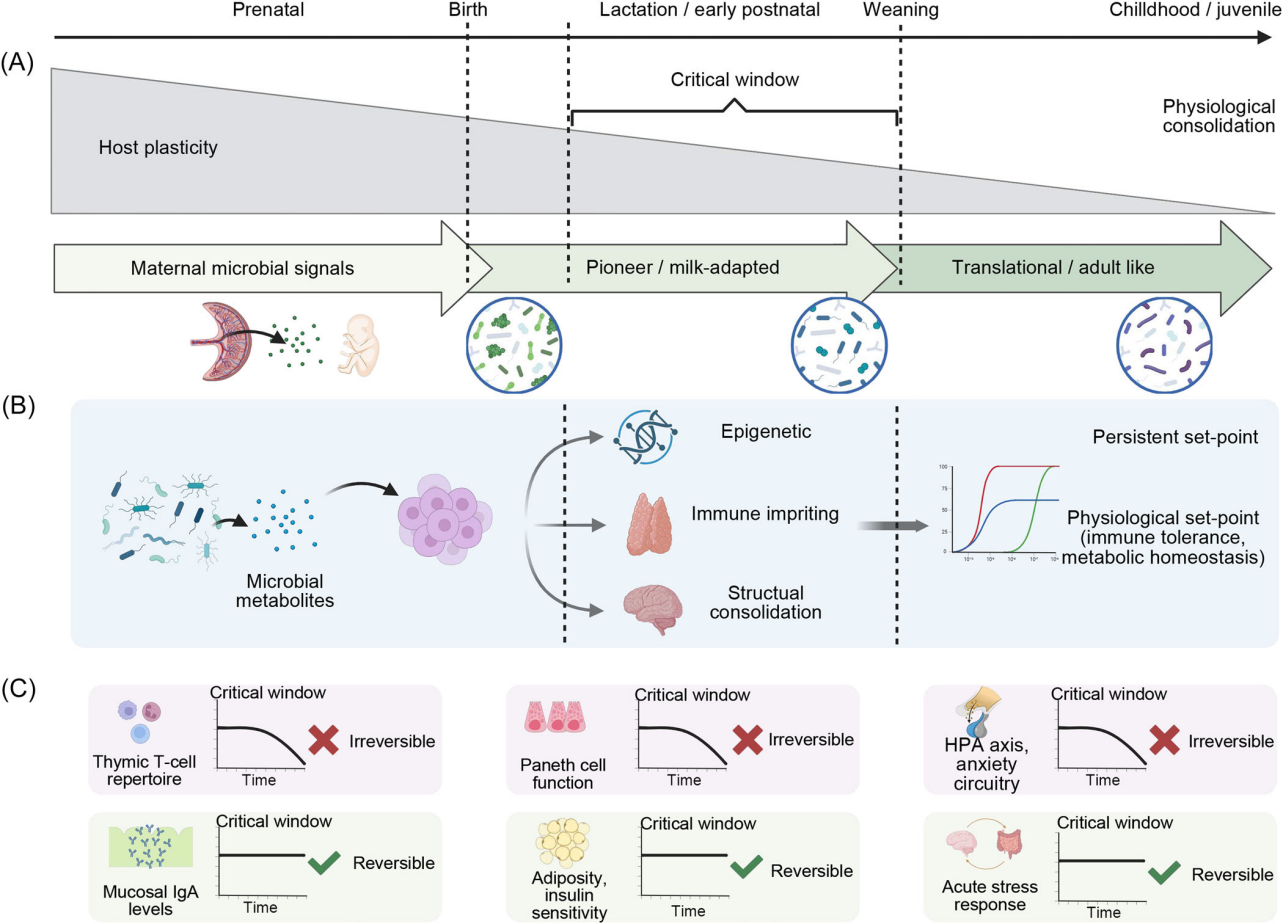
Temporal dynamics of host–microbe critical windows and the transition from plasticity to physiological consolidation. (A) Host plasticity and successional synergy. The host exhibits maximal developmental plasticity during the prenatal and early postnatal phases, which progressively declines as physiological systems consolidate. Host–microbial interactions evolve through three distinct stages: prenatal priming via maternal metabolites, pioneer colonization during lactation (milk‐adaptive), and functional maturation following weaning (translational/adult‐like). This temporal coupling ensures that microbial metabolic outputs are synchronized with host developmental milestones. (B) Biological embedding mechanisms. Microbial metabolites function as molecular signals that induce epigenetic modifications, immune imprinting, and structural consolidation in developing tissues. These interactions establish a “persistent set‐point” for systemic physiological parameters, such as immune tolerance and metabolic homeostasis, which calibrate the host's functional trajectory for the remainder of the lifespan. (C) Critical versus sensitive developmental windows. The reversibility of physiological imprinting is window‐dependent. Programs such as the thymic T‐cell repertoire, Paneth cell function, and hypothalamic–pituitary–adrenal (HPA) axis circuitry are governed by strict critical windows; once this temporal threshold is passed, perturbations become irreversible. Conversely, other systems, including mucosal IgA levels, adiposity/insulin sensitivity, and the acute stress response, retain plasticity throughout life, remaining reversible through targeted metabolic or ecological interventions even after the initial critical window has closed.

The “critical window” or “window of opportunity” in early life postulates that specific, often non‐redundant time frames exist during prenatal and postnatal development. During these windows, environmental exposures, particularly nutrition and microbial colonization, exert a disproportionate and enduring influence on host physiological programming [596, 597]. The validation and importance of this concept are supported by epidemiological and experimental evidence. This evidence demonstrates that environmental exposures, including microbiota colonization during particular early‐life timeframes, impact multiple host systems, including immune, metabolic, and neural circuits. Furthermore, they set a trajectory for long‐term health or disease susceptibility. Identification of the underlying mechanisms of these biological windows may reveal new targets for intervention. This implies that the timing of therapeutic measures is as critical as the therapy itself in promoting lifelong health [145, 304, 598] (Figure 9).

### Defining the critical “window of opportunity”

The physiological trajectory of microbial influence begins before birth with prenatal imprinting. During this phase, the fetus is exposed not to live microbes but to maternal microbial metabolites that traverse the placental barrier [472, 599]. These molecules influence offspring development through epigenetic modifications, metabolic receptor engagement, and modulation of cytokine networks, calibrating lymphoid and neural systems [83, 600, 601]. This intricate signaling dialog establishes the baseline for immune tolerance and metabolic homeostasis, embedding durable health trajectories within the maternal environment. Thus, the prenatal period constitutes a silent yet architecturally critical window, during which the immune, metabolic and neurological systems are calibrated through sustained molecular interaction prior to any direct encounter with live microbes [151].

After birth, the lactation period marks the first direct transmission of maternal microbiota through vertical seeding of pioneer taxa. This seeding is accompanied by mobile genetic elements that rapidly endow the infant microbiome with metabolic capacity [148, 602, 603]. Breast milk concurrently supplies oligosaccharides, immunoglobulins, and metabolites that shape the gut microbiota and promote immune education. This process establishes a foundational community whose composition influences tolerance, nutrient harvest, and growth [507, 508]. In preterm infants, the disruption of these maternally derived signals can lead to persistently stunted phenotypes and elevated susceptibility to inflammatory disorders, highlighting the enduring impact of this initial microbial endowment [604].

As the infant transitions to solid foods, the weaning period marks a succession phase characterized by drastic ecological remodeling [605]. The cessation of breastfeeding acts as a potent exogenous driver that fundamentally restructures the gut microbial ecosystem [606]. This triggers a sustained decline in *Bifidobacterium* and the emergence of fiber‐degrading taxa such as *Bacteroides*, *Prevotella*, and Clostridia [607]. This dietary shift simultaneously coincides with the withdrawal of protective maternal factors, including secretory IgA and lactoferrin. This withdrawal forces the infant's immune system to transition from passive protection to active defense, a process heavily reliant on the establishment of oral tolerance [509, 608, 609]. Weaning thus represents a vulnerable bottleneck in which the gut ecosystem must rapidly adapt to diverse food antigens while distinguishing them from pathogenic threats. Consequently, dysbiosis during this shift is strongly linked to later inflammatory and allergic diseases [303, 304].

Finally, the childhood and juvenile period represents an integration phase during which developmental focus shifts toward the refinement of host–microbiota interactions [287, 610]. This stage is characterized by sustained bidirectional communication between the microbial ecosystem and host physiological systems, including immune and endocrine pathways [611]. Unlike the many irreversible outcomes established during prenatal and early postnatal life, many physiological and developmental processes during childhood exhibit greater reversibility and are subject to considerably weaker temporal constraints. Additionally, the juvenile period may retain capacity for functional remodeling, and this plasticity has motivated microbiome‐targeted intervention studies in adolescents, including obesity and anorexia nervosa contexts [612, 613].

### Irreversible programming versus reversible modulation

While often used interchangeably, a distinction must be drawn between “critical” and “sensitive” periods. A critical period represents a strict temporal window during which specific inputs are obligate for proper system organization. If the expected exposure is violated, the resulting phenotype is often irreversible. In contrast, a sensitive period implies a broader timeframe where the system remains highly responsive but retains plasticity for later remediation, albeit with diminishing efficacy [614, 615].

This irreversibility versus reversibility can be illustrated across distinct physiological domains. Here, specific biological programs either “lock in” permanent fates or retain adaptive plasticity. In the immune system, the establishment of the foundational landscape often occurs within strict critical windows [146, 304]. For instance, the regulation of iNKT cells in the colon depends on specific microbial colonization during the early neonatal period. Failure to colonize during this window leads to irreversibly elevated mucosal iNKT levels and lifelong susceptibility to colitis and asthma [500]. Similarly, the development of adaptive immunity is also programmed early. A critical window exists for the thymic development of gut‐microbiota‐specific T cells. Within this window, early colonization is required for the trafficking of microbial antigens to the thymus to select specific T cell clones, a process that cannot be recapitulated in adulthood [616]. Similarly, IgE‐mediated sensitization is programmed during an early critical window that cannot be fully replicated later [519]. In contrast, IgA production represents a sensitive period. While an early‐life‐derived self‐sustaining B cell pool maintains steady‐state IgA, secretion levels remain modulable by new colonizers, diet, or environmental stimuli in adulthood [516, 518].

In the metabolic domain, the gut microbiota functions as an essential developmental component whose maturation must synchronize with host growth windows to prevent irreversible deficits [337]. Research by Jeffrey Gordon and colleagues established the concept of “microbiota maturity.” This research revealed that children with severe acute malnutrition possess an immature gut microbiome that fails to develop in time with their chronological age, a state that persists even after standard nutritional interventions [558]. Therapeutic MDCFs can partially repair this immaturity. Yet, growth stunting often remains partially irreversible because the critical growth window closes, effectively “locking in” the physical deficit [561]. Conversely, metabolic phenotypes such as adiposity operate within a more plastic sensitive window. Unlike the structural permanence of stunting, the efficiency of energy harvest and fat mass remains responsive to diet and microbial modulation throughout life. Interventions such as caloric restriction or exercise in adulthood can effectively reverse hypertrophy and improve insulin sensitivity, demonstrating that metabolic health remains tunable despite early‐life programming [617].

In the neural domain, the gut microbiota exerts a profound influence on brain development through a strict dichotomy of critical and sensitive periods. Research using GF mice has identified an absolute critical developmental window for the programming of the hypothalamic–pituitary–adrenal (HPA) axis and anxiety‐related behaviors [426]. GF mice exhibit an exaggerated HPA stress response and altered expression of key neurotrophic factors and 5‐HT receptors in the hippocampus and amygdala. Crucially, while reconstituting the microbiota with specific commensals at an early postnatal stage can normalize these stress responses and neurochemical deficits, colonization in adulthood fails to reverse the phenotype [426]. This indicates that the neural circuitry governing anxiety and stress resilience is structurally “locked in” during this early window. Furthermore, the absence of microbial signals leads to irreversible behavioral and neuroendocrine consequences. Other aspects of the gut–brain axis, such as stress‐induced dysbiosis, remain dynamic and reversible across the lifespan [618, 619].

### The temporal synchronization of host plasticity and microbial succession

The definition of critical windows rests on the precise temporal coupling between host ontogeny and microbial ecological succession. Critically, the defining criteria are not a binary choice between host physiological maturity and microbial community succession patterns. Rather, the two are obligately intertwined and jointly operationalize the window boundaries [304]. This period represents a transient state of heightened physiological receptivity. During this time, the host possesses a unique capacity to integrate environmental signals into its developing biological architecture. This “open” state facilitates the initial colonization and education of the host physiology [471]. However, this plasticity is finite. As development progresses, physiological consolidation occurs. This is driven by processes such as the strengthening of the physical intestinal mucosal barrier, the completion of neural myelination, and the shift of the immune system from an educational to a defensive posture [304, 620]. These terminal events mark the structural closure of the critical window, after which the capacity for systemic remodeling is significantly attenuated.

Crucially, this developmental window of host plasticity is contingent upon functional synergy with an age‐defined but rapidly changing microbial consortium [204, 621]. Host developmental stages (prenatal priming via maternal metabolites, lactation‐dependent passive immunity requiring milk glycan‐utilizers, weaning‐induced barrier maturation, and post‐weaning immune consolidation) provide the physiological scaffolding that dictates which microbial functions are essential. Concurrently, the succession of microbial communities, from *Bifidobacterium* and *Lactobacillus* during lactation to fiber‐degrading Firmicutes and Bacteroidetes after weaning, supplies the specific metabolic signals (sialylated compounds, acetate, indolelactic acid, SCFAs) necessary for synaptic formation, microglial maturation, and immune maturation [622, 623, 624].

In practice, this means that a window cannot be defined solely by the postnatal day. Instead, it must be anchored to host maturational events, such as gut epithelial closure, thymic involution, or the completion of myelination. This coincides with microbial metabolic transitions, such as the shift from sialylated HMO fermentation to complex fiber degradation. It is the alignment of these two independently progressing but coevolved programs that creates the transient state of heightened receptivity. Equally importantly, it determines its closure when either partner progresses beyond the coordinated phase. A disruption in succession timing, such as the prolonged persistence of an infant‐type microbiota beyond weaning, decouples the metabolic input from the host's receptive state. This decoupling embeds a permanent mismatch into tissue architecture and physiological setpoints [303, 304]. Thus, the early windows of opportunity are not passive intervals of susceptibility, but evolutionarily conserved checkpoints at which host ontogeny and microbial succession are obligately intertwined [625, 626].

### From early exposure to long‐term phenotypes: Causality and mechanisms

The long‐term consequences of exposures during critical developmental windows constitute a central focus of current research [627, 628]. A fundamental question persists regarding how transient early‐life events translate into persistent phenotypes that may manifest decades later. This process, often conceptualized as “biological embedding” or “imprinting,” is not merely a functional adaptation but a structural transition from developmental plasticity to physiological consolidation. This transition is governed by distinct but interconnected epigenetic, cellular, and systemic layers [304, 629].

At the genomic level, the persistence of early‐life signals can be mediated through inheritable epigenetic marks that continue to exert regulatory functions later in life [551, 629]. Microbial metabolites such as butyrate function as HDAC inhibitors, actively modulating chromatin accessibility [630]. The establishment of these epigenetic markings can influence key physiological functions across the lifespan. For instance, in a landmark study by Furusawa et al., microbially derived butyrate was shown to inhibit HDAC. This led to enhanced histone acetylation at the *Foxp3* locus in colonic T cells. This epigenetic modification established a permissive chromatin state that promoted stable *Foxp3* expression and Treg differentiation, a phenotype that persisted throughout the cell lineage [491].

At the cellular level, early exposures define the organism's cellular characteristics by influencing differentiation and establishing immunological imprinting. The concept of early‐life “imprinting” is most evident in studies utilizing GF animal models. GF mice, lacking microbial colonization, exhibit aberrant immune development. For instance, GF mice exhibit a reduced population of Treg cells, coupled with an imbalance in Th1–Th2 responses. This suggests that the absence of microbial cues results in a failure to properly imprint the immune system [472, 609, 631]. When the natural microbial signals necessary for balanced T cell differentiation are absent or altered, it may result in a skewed immune state that predisposes an organism to chronic inflammation or autoimmunity [303]. In some cases, the reconstitution of GF mice with specific microbes reverses these deficiencies. This reinstates a balanced and appropriately imprinted immune landscape [501]. This phenomenon underscores the notion that early microbial exposures define and program cellular characteristics with lasting consequences.

Establishing a definitive causal chain from early exposure to distant clinical outcomes in humans remains challenging but is advancing through sophisticated study designs. Longitudinal birth cohorts that track subjects from pregnancy into adulthood, collecting serial biological samples and clinical data, are indispensable for mapping these trajectories [147, 632]. Sibling‐pair studies further assist in controlling for shared genetic and environmental variables [570, 633]. To transcend associative findings, researchers increasingly employ methods such as Mendelian randomization, using genetic variants as instrumental variables to infer causality between microbiota features and disease states [634, 635]. Furthermore, the identification of mechanistic intermediate phenotypes, such as specific immune cell ratios, metabolite profiles, or specific epigenetic signatures, provides a biologically plausible bridge connecting early‐life events to later disease. This biological plausibility significantly strengthens causal inference.

### Perspective and limitations

Overall, the early‐life period, spanning from preconception to the first few years after birth, constitutes a series of interconnected critical windows [304, 620]. During these windows, nutrition and microbial colonization act as primary environmental drivers that shape the development and long‐term setpoints of the immune, metabolic, and nervous systems. However, the majority of existing studies have predominantly emphasized early life as a homogeneous phase. They have focused on the overarching role of the gut microbiota in host physiology and pathology across the entire window. In doing so, they have largely overlooked the functional heterogeneity among distinct critical windows and their differential contributions under varying conditions. This prevailing “whole‐window” perspective may obscure the unique impact of specific developmental windows, particularly regarding how host–microbe interactions dynamically shift in response to variables such as delivery mode, dietary interventions, antibiotic exposure, or host genetic background. To dissect these window‐specific mechanisms and assess their reversibility, future research should incorporate more refined experimental models, such as reversible colonization strategies, time‐window‐restricted gnotobiotic models, and antibiotic perturbation models [516, 636]. Recognizing these windows is not merely an academic exercise. Rather, it unveils a powerful opportunity for preventive and therapeutic interventions.

### Multi‐system regulatory network of core microbial metabolites

The maternal–infant microbiome constitutes far more than a passive, localized aggregation of microorganisms; it functions as a profoundly dynamic, highly integrated, and multi‐system endocrine organ. At the molecular core of this complex host–microbe crosstalk lies a diverse and potent array of bioactive microbial metabolites. While taxonomic and metagenomic profiling provides a foundational structural blueprint of the early‐life gut ecosystem, it is the functional currency of these microbially derived metabolites, most notably SCFAs, BAs, and tryptophan‐derived indoles that mechanistically bridges the mucosal microbiota with distant host target organs. These pleiotropic molecules are capable of traversing the intestinal barrier, entering the systemic circulation, and actively crossing both the placental interface and the BBB [363, 392, 472].

During the strictly timed critical developmental windows that span from prenatal priming through early childhood integration, these core microbial metabolites operate synergistically to program the host's neuro–immuno–metabolic axes. They function through highly diverse mechanisms, acting variously as direct bioenergetic substrates for host cells, robust epigenetic modifiers, and highly specific receptor ligands [304, 371, 481]. By dynamically calibrating mucosal and systemic immune tolerance, guiding the structural wiring of neural circuits, and establishing lifelong metabolic homeostasis, these metabolites encode the biological memory of early‐life microbial exposures. Understanding the comprehensive multi‐system regulatory network of these core metabolites is a clinical imperative. This understanding is essential for deciphering how transient ecological shifts and dysbiotic states translate into enduring physiological phenotypes and long‐term disease susceptibility.

#### Short‐chain fatty acids: Systemic epigenetic and metabolic regulators

SCFAs represent the primary metabolic end products of anaerobic microbial fermentation, utilizing indigestible dietary fibers and HMOs as their core substrates [295, 301]. The regulatory influence of SCFAs is vast, extending across almost every physiological compartment during early‐life development. At the MFI, SCFAs act as crucial metabolic and vascular amplifiers. As pregnancy progresses into mid‐gestation, maternal gut‐derived metabolites may contribute to maternal–fetal interface adaptation and fetal growth regulation through gut–placental signaling pathways [95]. This receptor–ligand interaction promotes local angiogenesis and robustly upregulates trans‐barrier glucose and lipid transport mechanisms. This ensures that an adequate and continuous nutrient flux is maintained to support the rapidly expanding fetus.

In the developing postnatal infant gut, SCFAs are absolutely foundational for the dual establishment of physical barrier integrity and mucosal immune education. Butyrate, in particular, serves as the primary oxidative bioenergetic fuel for colonocytes; it stimulates epithelial cell proliferation and directly upregulates the expression of critical tight junction proteins to fortify the intestinal physical barrier against opportunistic pathogen invasion [302, 481]. Immunologically, SCFAs act as extraordinarily potent endogenous inhibitors of HDACs [363, 371, 551]. By actively maintaining an open and transcriptionally permissive chromatin state, butyrate facilitates targeted histone acetylation at the *Foxp3* locus [491]. This epigenetic imprinting directly drives the differentiation, maturation, and stable expansion of colonic RORγt^+^ Tregs [303, 490, 491, 492]. This mechanism establishes a deeply tolerogenic mucosal environment that effectively restrains pathological hyperreactivity to commensal antigens and novel dietary proteins, fundamentally lowering the host's longitudinal risk of developing atopic diseases, allergies, and autoimmune conditions [303, 304].

The neurodevelopmental impact of microbially derived SCFAs is equally profound and structurally determinative. Systemically circulating SCFAs readily cross the BBB, exerting direct structural, immunological, and functional control over the developing CNS [363, 392]. Paradoxically, they are indispensable architects of the BBB itself. Early‐life butyrate exposure restores and maintains BBB tightness by upregulating the expression of tight junction proteins, such as claudin‐5 and occludin, through HDAC inhibition within brain endothelial cells [392]. Within the deep brain parenchyma, SCFAs provide critical epigenetic licensing for neurogenesis, neuroplasticity, and cellular survival. By inhibiting HDACs at the promoters of neurotrophic genes, SCFAs actively promote the robust transcription of BDNF and NGF, both of which are absolute prerequisites for neuronal survival, dendritic arborization, and synaptic circuitry formation [363]. Furthermore, specific SCFAs such as acetate serve as direct and preferential bioenergetic substrates for microglial mitochondria. This actively rescues bioenergetic dysfunction and guides microglial functional maturation into a homeostatic, neuroprotective surveillance phenotype [372]. In the context of the brain's white matter, SCFAs such as butyrate can reduce oligodendrocyte precursor cell loss by inhibiting astrocyte activation through the SGK1–IL‐6 signaling pathway [390]. Separately, SGK1 signaling has also been implicated in hippocampal demyelination and diabetes‐associated cognitive dysfunction, further supporting its relevance to metabolic regulation of myelin integrity [637].

Metabolically, SCFAs serve as central programmers of early‐life appetite regulation and systemic energy balance. By binding to and activating GPR41 and GPR43 receptors located on enteroendocrine l‐cells in the gut epithelium, propionate and butyrate trigger the rapid secretion of potent satiety hormones, most notably GLP‐1 and PYY [572]. This sophisticated gut–brain signaling axis effectively delays gastric emptying and directly suppresses hypothalamic feeding centers. This provides a physiological defense mechanism against excessive weight gain and childhood obesity [572, 573]. Additionally, SCFAs continuously modulate the gut–liver axis. By acting on hepatic receptors, they reduce de novo lipogenesis and systemic inflammation, thereby offering early architectural defense against the progression of pediatric MAFLD [580]. In summary, SCFAs act as master metabolic rheostats that perfectly synchronize intestinal energy harvest with systemic immune tolerance and profound neuroplasticity.

#### Bile acids: Metabolic synchronizers and inflammatory rheostats

BAs are initially synthesized in the host liver as primary BAs and are subsequently secreted into the intestinal lumen, where they are extensively biotransformed by the gut microbiota into a highly diverse pool of secondary BAs [583]. This critical microbial biotransformation dictates the ultimate signaling capacity of the BA pool across various host systemic receptors, primarily the FXR and TGR5. During late pregnancy, a period when the maternal system faces its peak metabolic demands and rising inflammatory loads, BA signaling acts as a vital and dynamic homeostatic buffer. The targeted activation of placental FXR and TGR5 receptors helps maintain maternal–fetal metabolic stability while actively restraining excessive, potentially dangerous inflammation [93]. Specifically, BA‐related signaling can suppress NLRP3 inflammasome activation and pro‐inflammatory immune responses at the maternal–fetal interface, thereby helping maintain a less inflammatory environment during pregnancy [106].

Postnatally, the microbial regulation and conversion of the BA pool critically dictate both gastrointestinal survival and systemic health. In the highly vulnerable context of the premature infant, profound microbial dysbiosis severely alters the BA profile, frequently elevating the intestinal levels of cytotoxic primary BAs, such as chenodeoxycholic acid (CDCA), which can rapidly induce an inflammatory “leaky gut” state [222]. Conversely, commensal‐mediated biotransformation of BAs provides potent, life‐saving protection against catastrophic neonatal morbidities such as NEC. Specific pioneer microbes carrying *bsh* genes possess the functional capacity to aggressively lower cytotoxic taurodeoxycholic acid (TDCA) levels, thereby attenuating severe intestinal epithelial injury through the protective FXR–NLRP3 pathway [258]. This precise interaction highlights the indispensable role of secondary BAs as direct mediators of mucosal survival under conditions of extreme inflammatory stress.

Beyond the local gastrointestinal tract, microbially modified BAs exert far‐reaching and durable effects on systemic lipid metabolism and early neural development. Within the gut–liver axis, the altered BA profiles characteristic of early‐life dysbiosis can actively antagonize intestinal FXR signaling. This antagonism paradoxically increases hepatic lipogenesis while simultaneously reducing fatty acid oxidation, propelling the early pathogenesis and fibrotic progression of pediatric MAFLD [584, 585]. Within the developing brain, specifically modified secondary BAs, such as TDCA, are capable of crossing the BBB to directly regulate cellular mitochondrial function and modulate Notch signaling pathways [364]. These pathways are absolute prerequisites for neural stem cell proliferation, fate determination, and ongoing postnatal neurogenesis [364]. Through their dual, highly specialized roles as lipid solubilizers and pleiotropic signaling molecules, BAs form an indispensable, dynamically regulated link between the microbiota, hepatic lipid metabolism, and central neuro‐immune regulation.

#### Indoles and tryptophan metabolites: Neuro‐immune calibrators

The essential amino acid tryptophan serves as a critical and competitive node in host–microbe metabolic crosstalk. Gut bacteria directly metabolize unabsorbed luminal tryptophan into a wide variety of indole derivatives, including indole‐3‐acetic acid and indole‐3‐lactic acid. These derivatives act as exceptionally potent exogenous ligands for the host's AhR [85, 373, 394]. The “microbiota–tryptophan–AhR” axis constitutes a fundamental and evolutionarily conserved mechanism of early‐life immune programming and neurological defense.

During the highly sensitive first trimester of pregnancy, maternal gut‐derived indoles are transported to the MFI, where they engage AhR that is highly expressed on uterine natural killer (uNK) cells, resident Tregs, and invading trophoblasts [85, 87]. This precise molecular activation actively redirects uNK cells away from a default cytotoxic posture toward a specialized angiogenic phenotype. This promotes the robust secretion of VEGF and facilitates crucial spiral artery remodeling [90, 91, 92]. Furthermore, this specific pathway establishes selective maternal–fetal immune tolerance via the IDO pathway, firmly preventing maternal immune rejection of the allogeneic fetus and securing early pregnancy viability [88, 89].

In the rapidly developing neonate, gut‐derived indole metabolites are equally vital for ongoing immune and neurological calibration. Within the intestinal mucosa, microbial AhR ligands strongly stimulate the proliferation and functional maturation of ILC3s, driving the continuous baseline secretion of IL‐22 [472, 475]. This specific interleukin is critical for inducing Paneth cells to secrete high concentrations of potent AMPs [475, 476]. These AMPs maintain the physical and chemical segregation of commensal bacteria from the sensitive intestinal epithelium, thereby preventing systemic bacterial translocation and the onset of neonatal sepsis [475, 476].

Within the CNS, microbial indoles govern local glial cell phenotypes and directly activate AhR signaling in brain‐resident astrocytes and microglia [395]. This targeted activation upregulates critical neuroprotective factors, such as SOCS2, while simultaneously and potently suppressing NF‐κB‐mediated transcription of neurotoxic cytokines [373, 374, 375, 395, 396]. By actively pacifying the local glial environment, the intestinal microbiota prevents aberrant microglial activation and protects against secondary BBB breakdown, effectively locking out systemic inflammation from the exquisitely vulnerable developing brain [395].

Additionally, the microbiota directly regulates the alternative, host‐driven pathway of tryptophan metabolism: the synthesis of 5‐HT. Specific spore‐forming commensals upregulate the expression of the *Tph1* enzyme in colonic enterochromaffin cells, driving massive peripheral 5‐HT production [366]. Because 5‐HT acts as a vital trophic and guidance factor during early brain development, it explicitly guides neurogenesis, circuitry formation, and the programming of the HPA stress response. Consequently, dysbiosis‐induced fluctuations in the tryptophan–5‐HT axis can directly alter the structural wiring of the CNS [365, 366, 367, 638]. This dysregulation provides a clear mechanistic basis for how early‐life microbial imbalances predispose infants to complex neurodevelopmental and neuropsychiatric disorders, including ASD and ADHD [638, 639].

#### Integration: The multi‐system crosstalk

The physiological impact of these core microbial metabolites is neither isolated, linear, nor redundant; rather, they form a highly integrated, tightly coordinated multi‐system regulatory network. SCFAs, BAs, and indoles operate continuously in tandem to construct and secure a synchronized host developmental program. For instance, while SCFAs epigenetically open the neuronal chromatin landscape to actively permit neurotrophin expression [363], secondary BAs simultaneously ensure the metabolic and mitochondrial fitness of neural stem cells, allowing them to appropriately respond to these trophic signals [364]. Concurrently, indoles actively suppress the neuroinflammatory glial responses that would otherwise excessively prune or destroy these nascent, highly vulnerable neural networks [373, 374, 375].

Similarly, within the gastrointestinal tract, the precisely coordinated action of SCFA‐driven Treg induction [491] and indole‐driven ILC3–IL‐22 mucosal defense [475] operates collectively to secure lifelong immune homeostasis. This deeply integrated metabolic signaling network underscores that the early‐life microbiota functions not as a collection of localized bystanders, but as a widely distributed, life‐sustaining endocrine organ. Deficits or imbalances in any single arm of this core metabolite triad can rapidly precipitate cascading functional failures across the neuro–immuno–metabolic axes, manifesting clinically as distinct and severe pediatric pathologies [83, 304]. Consequently, future translational diagnostics and therapeutic interventions must decisively pivot from targeting singular microbial taxa toward functionally restoring this comprehensive metabolic network. Utilizing defined microbial consortia, targeted prebiotics, or precision postbiotics to re‐establish the balance of SCFAs, BAs, and indoles will be essential to ensuring the harmonious structural and functional maturation of the human holobiont across its most critical developmental windows.

### Translational aspects

**FIGURE 10:**
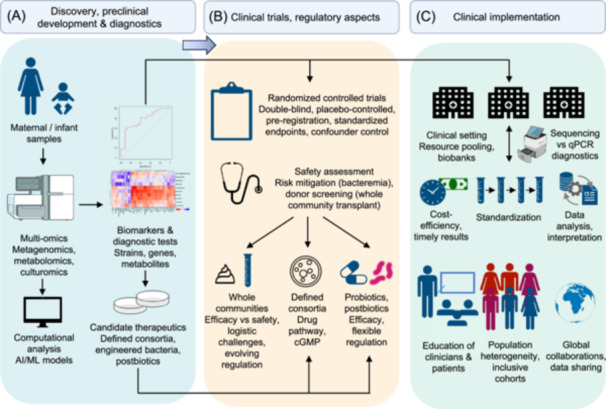
Translational framework for microbiome‐based diagnostics and therapeutics in maternal and infant health. (A) Discovery, preclinical development, and diagnostics. Clinical integration begins with the collection of maternal and infant samples, followed by multi‐omics profiling (metagenomics, metabolomics, and culturomics). These data are processed using computational analysis and artificial intelligence (AI) and machine learning (ML) models to identify robust microbial biomarkers or diagnostic tests based on specific strains, genes, or metabolites. This phase also includes the development of candidate therapeutics, such as defined bacterial consortia, engineered microbes, and postbiotics. (B) Clinical trials and regulatory aspects. To ensure clinical value, microbiome interventions must be evaluated through rigorous, double‐blind, placebo‐controlled randomized clinical trials with pre‐registered, standardized endpoints and careful control of confounders (e.g., diet, medications). Safety assessments and risk mitigation strategies are paramount, particularly regarding donor screening for whole‐community transplants. Interventions span a spectrum from whole‐community transplants to defined bacterial consortia (manufactured under current good manufacturing practice (cGMP) conditions) and probiotics/postbiotics, each requiring tailored regulatory frameworks and efficacy/safety validation. (C) Clinical implementation. Successfully translating microbiome tools into clinical settings requires overcoming logistical and economic bottlenecks. Essential steps include pooling resources (e.g., biobanks) across centers, moving toward cost‐effective and standardized diagnostics (e.g., quantitative polymerase chain reaction (qPCR) vs. sequencing), and optimizing data analysis pipelines for timely clinical interpretation. Widespread adoption also depends on inclusive cohorts that account for global population heterogeneity, robust international data sharing, and the continuous education of both clinicians and patients regarding the limitations and potential of microbiome technology.

Microbiome characterization in humans has advanced substantially since the pioneering efforts of the Human Microbiome Project (HMP) [640] and Metagenomics of the Human Intestinal Tract (MetaHIT) [641]. However, translating this knowledge into the clinic remains an open problem, particularly in diagnosing conditions or predicting their likely course in mothers and infants. Although microbial therapeutics in maternal–infant health have shown promise in research settings [182, 642], they are generally not yet approved for clinical use. In this section, we will describe translational microbiome applications, including diagnosis and interventions in mothers and infants. We will then review safety and regulatory considerations, the need for interpretable trials that can be generalized, and finally discuss bottlenecks and pathways for implementation (Figure 10).

### From bench to bedside: Are microbiome‐based diagnostics ready for the clinic?

Multiple studies have identified microbial biomarkers across diseases, including IBD [643], vaginal health [644], rheumatoid arthritis [645], psoriatic arthritis [646], allergies [524], or sepsis risk [205]. Recent studies have also proposed health signatures derived from microbiome data [647, 648, 649]. Despite these results, there are currently no clinically approved microbiome diagnostics.

Several factors contribute to the lack of translation of microbiome research into clinical practice. For instance, microbiome diagnostics might be logistically challenging or less cost‐effective than existing clinical standards, such as the culture‐based detection of pathogens. Approaches that refine research findings into easily implementable tests, such as quantitative polymerase chain reaction (qPCR)‐based diagnostics, could facilitate clinical translation. Furthermore, factors such as diet [570, 650], medications [651], and genetics [652, 653] can all impact the microbiome. These factors might bias microbiome‐based diagnostics in specific cohorts or populations [654]. External validation cohorts are thus fundamental to identify robust microbial biomarkers associated with disease. This is particularly relevant since omics research has generally focused on cohorts from developed countries [655], a bias that also pervades microbiome work [656]. More inclusive microbiome studies across diverse human populations are therefore strongly needed [657, 658].

Changes in the microbiome may also be a consequence rather than a cause of disease, which might result in poor diagnostic accuracy in early‐stage patients. Studies of treatment‐naïve, newly diagnosed patients are thus critical for developing accurate microbiome diagnostics [659, 660]. While machine learning (ML) methods are commonly used in microbiome diagnostics [661, 662], their performance often does not improve upon existing clinical algorithms. Notably, for ML approaches to yield accurate predictions, microbiome studies will require cohorts of thousands of samples, as is common in genetics [663, 664, 665]. Although microbiome meta‐analyses have been explored to increase statistical power [643, 666, 667], they are often constrained by the limited amount of confounder information available in the original publications. Well‐established confounders, such as diet or antibiotic use, should be uniformly reported following community standards [668, 669] to facilitate the identification of robust biomarkers. Finally, diagnostics based on microbial functions might be more generalizable than those based on taxonomy. Bacterial strains are highly unique to individuals [670] and less likely to be shared among unrelated patients. This individuality could partially explain the inconsistencies in biomarkers reported across different studies. Pathogenic functions, however, can be conserved across different strains, suggesting that they could serve as more accurate, mechanistic biomarkers.

### Microbiome interventions in infants and mothers

Delivery mode, breastfeeding, and the introduction of solid foods represent “natural” interventions shaping the early‐life microbiome. To mimic how vaginal delivery modulates colonization of the newborn, interventions that transfer the maternal vaginal microbiome to cesarean‐delivered infants have been proposed [181]. These interventions modify the microbiome of cesarean‐delivered infants to resemble that of vaginally delivered infants, alter their metabolomic profile, and improve neurodevelopmental outcomes [182]. Their use in other conditions, such as food allergies, is currently being investigated [671]. The use of formula milk mixed with maternal stool has also been shown to restore the microbiome of cesarean‐delivered infants, making them more similar to vaginally delivered infants [672].

While not explicitly tested in mothers, vaginal microbiome transplantation from healthy women to patients with intractable bacterial vaginosis has been explored [642]. Four out of five patients experienced long‐term remission, although three required repeated transplants. Promoting changes in the maternal microbiome has also been considered as a strategy to benefit infants. The colonization of dams with specific microbes can modulate the immune development of the offspring through maternal antibodies transmitted during pregnancy and via breast milk [472]. In humans, maternal dietary interventions are being tested as a strategy to modulate the microbiome of pregnant women and their infants, although the benefits of such approaches remain to be established [673].

While microbiome therapeutics have shown efficacy in randomized trials for preventing recurrent Clostridioides difficile infection (rCDI) [674, 675], no such interventions are approved specifically for infants or mothers in any condition. Various studies have claimed the benefits of prebiotics and probiotics in infants, although results are often inconsistent and high‐quality randomized trials are lacking. Investigating the mechanisms by which bacteria exert a therapeutic effect can provide more robust evidence. For example, we have recently demonstrated that bacteria encoding a DL‐endopeptidase modulate the expression of the NOD2 receptor and protect against sepsis in preterm infants [205]. Importantly, the question of why only certain subjects respond to microbial interventions remains unanswered. Although the environment is a major determinant of microbiome composition [676, 677], host genetics can also modulate microbial colonization [652, 653, 678]. Evidence from FMTs suggests that an intensive therapeutic regimen is likely important to induce a response [679]. Consequently, we hypothesize that the inconsistencies observed in previous studies could be attributable to dose effects. Finally, the baseline microbiome of the patient prior to intervention could also explain response heterogeneity, as some microbiomes might be more tolerant to foreign colonization [570, 680, 681].

### Interpretable, generalizable microbiome clinical trials

For microbiome interventions to be of value, clinical trials need to be interpretable and generalizable. Pre‐registration in public registries with well‐defined hypotheses, methods, and endpoints should be required, with primary endpoints based on clinical (e.g., Mayo score in IBD) or physiological measures (e.g., IgE levels in food allergies). Mechanistic or secondary endpoints can quantify changes in biochemical properties of interest, such as microbiome or immune profiles. While understanding the effect of an intervention on the microbiome can be scientifically significant, we argue that the microbiome should be strictly considered as a secondary endpoint in clinical trials.

When possible, microbiome interventions should adopt a double‐blind, placebo‐controlled randomized design. The use of heat‐killed bacteria and inert substances as placebos, alongside stratified randomization to account for heterogeneity, should also be encouraged. The use of adaptive trial designs can also improve efficiency compared to traditional implementations: they are more ethical, as preliminary findings can identify participants likely to benefit from reallocation, and trials can be terminated early if a lack of efficacy is demonstrated [682].

The inherent tradeoff between the homogeneity of the target population and the sample size in clinical trials requires a careful consideration of inclusion and exclusion criteria. Age, sex, weight, diet, or medications can act as confounders in microbiome studies [683], although their relative effect sizes in the microbiomes of mothers and infants remain unclear. We thus propose that, except for clearly established factors such as antibiotics or pre‐existing gastrointestinal disorders, the rigorous recording of confounders might be preferable to strict exclusion criteria. Trials with more inclusive enrollment have several advantages, including higher statistical power and the possibility of longitudinal sampling from a larger pool of participants, and confounders can be adjusted for a posteriori. Importantly, the relative importance of confounders can change over time: a recent meta‐analysis of food allergies in infants identified delayed solid food introduction, antibiotics, and Cesarean delivery as risk factors, but, surprisingly, not breastfeeding [684]. Given the known effect of these variables on the microbiome [204], we argue for the need to design trials that reflect a wider proportion of the population under realistic scenarios.

### Safety and regulatory aspects

Translating microbiome research to the clinic requires the safe, regulated implementation of diagnostic and therapeutic approaches. Gut microbiome diagnostics generally have a safe profile, as fecal specimens do not require invasive sampling. Microbial therapeutics, however, require a careful assessment of various safety aspects. Prebiotics, probiotics, and synbiotics have been extensively used in clinical and research settings, and although bacteremia is rare, cases have been reported. In a large pediatric cardiac‐surgery cohort in China, probiotic‐strain bacteremia was identified in six children, including cases suggesting possible cross‐contamination or catheter‐related transmission [685]. Reports have described bacteremia by *Bifidobacterium longum* and *B. longum* subsp. *infantis* [686, 687, 688], as well as *Saccharomyces cerevisiae* fungaemia related to probiotic use [689], highlighting safety concerns when live biotherapeutics are used in vulnerable infants.

Importantly, the definition of “‐biotics” can vary depending on the regulatory framework. Probiotics are defined as “food” in most countries, with some classifying them as “health supplements.” The health benefit claims are also highly dependent on the specific country, resulting in a regulatory paradox: while probiotics are defined as “live microorganisms that confer a health benefit on the host” [134], the health claims need to be backed by evidence only in some countries, the nature of the evidence is not always agreed upon, and the health benefit is often loosely defined. In the United States, probiotics are categorized based on their intended health outcome, yet the FDA has not approved probiotics for clinical use in infants, while in the European Union, the term “probiotic” is not even recognized as a health claim [690]. In China, probiotics can be marketed as “health foods” with approved functional claims, but the regulatory framework is particularly stringent for pediatric applications, with usage restricted to a narrow list of strains. Japan uses two different systems for claim substantiation: one requiring demonstrated safety and efficacy in a clinical trial, and a more flexible path that can be simply based on systematic reviews. Overall, the regulatory landscape of “‐biotics” is highly complex and remains an important hurdle for translational applications.

The transfer of whole microbiome communities introduces additional safety and regulatory considerations. The transplant of a healthy vaginal microbiome to women with bacterial vaginosis is effective in treating the condition [642], but it remains unclear whether this approach can be safely used in pregnant women. Because the transplanted community is not manufactured, it is not possible to establish a quality control process equivalent to that of traditional drugs. This issue also affects procedures in which newborns are colonized with maternal microbiomes, such as those involving vaginal seeding [181, 182] or formula mixed with maternal stool [672]. The maternal donors in these approaches are rigorously screened for pathogens, and neither method has found differences in adverse events compared to control groups, supporting the idea that the interventions are safe when appropriately performed. However, these procedures remain exploratory, and further studies will be required to characterize safety and potential health benefits.

Patient safety is a fundamental medical precept of particular importance in vulnerable populations such as mothers and infants, and we advocate for the careful consideration of lessons learned from microbiome transplant studies. While controlled trials and systematic reviews generally suggest that adverse events are uncommon and not consistently higher than placebo in selected settings [182, 691, 692], safety evidence in broader immune‐mediated diseases remains under evaluation, including ongoing systematic review protocols [693].

The criteria for selecting microbiome donors continue to evolve. Donor exclusion criteria now consider the risk of gastrointestinal comorbidities, infections, or antibiotic resistance, among many others [694]. Frameworks for end‐to‐end donor screening that evaluate medical history and include comprehensive laboratory testing in conjunction with manufacturing controls (minimization of contaminants, inactivation/removal of extraneous agents, bioburden quantification) can further minimize microbiome transplant risks [695]. In addition, rigorous criteria should be established to exclude certain recipients from microbiome transplants, such as immunocompromised patients [696]. As an alternative to whole microbiota transplantation, the use of defined bacterial consortia has gained traction recently, given their superior safety profile, advantages for product consistency, and flexibility to test specific mechanisms. However, despite their success in the treatment of rCDI, their efficacy remains limited in multifactorial diseases such as ulcerative colitis, in which FMTs have demonstrated better efficacy [697]. The production of bacterial consortia also requires current good manufacturing practice facilities, which are costly and generally focused on producing defined molecules. Translational impact can be maximized if both whole and defined microbial community transplants continue to be investigated while maintaining appropriate safety standards. Whole community transplants have demonstrated efficacy in complex diseases [679] and also as co‐adjuvants of immunotherapy [698]. The precise mechanisms of action, however, remain poorly understood. Exploring parallel approaches based on whole and defined communities is thus likely to provide complementary insights that will benefit patients.

### Gaps in translational microbiome research

Important gaps remain in the implementation of microbiome‐based assays in the clinic. Most studies continue to be primarily focused on bacteria, as they make up most of the microbial biomass in fecal samples. Viruses, fungi, and archaea have also been shown to be important modulators of host responses [699], yet their role during pregnancy and early life remains understudied. The absolute abundance and viability of microbial cells in samples also remain largely unaddressed. The use of relative abundance is a well‐known issue in microbiome analysis [700], and different software tools have been developed to limit this bias, although disagreements exist on the merits of each method [701, 702]. Alternatively, experimental approaches can be used to obtain absolute quantification [703]. Microbial viability represents a more challenging task, as sequencing‐based approaches cannot determine the physiological state of microbial cells. This distinction is fundamental to determining the capacity of bacteria to produce specific metabolites, as well as host responses through microbial viability‐associated pathogen‐associated molecular patterns [704]. Microbial interventions should also consider whether the transferred microbes remain viable after transplantation and how viability might associate with health outcomes.

Several practical aspects limit the integration of microbiome assays into clinical workflows. Sequencing‐based microbiome diagnostics require a large number of samples to be cost‐effective, which in turn requires automated preparation to reduce processing time and heterogeneity [157]. Smaller hospitals are unlikely to have the sequencing and computational resources to perform high‐throughput microbiome testing, and the pooling of resources across centers through centralized repositories would lower costs and reduce turnaround time, thus facilitating clinical implementation. The development of qPCR‐based diagnostics based on initial biomarker discovery, as exemplified in IBD [643], would further reduce cost and effort. However, tests purely based on taxonomic abundance might be limited in their predictive accuracy for specific populations [654]. Thus, it will be critical to develop truly generalizable models, which will also guide the development of interventions specifically tailored to different populations. Facilitating international cooperation, the adoption of common standards, and the sharing of results will thus be fundamental to maximizing the translational impact of microbiome research.

Education of clinicians and patients will also be critical to ensure that the benefits of clinical implementation are based on robust scientific evidence. Microbiome technology has changed radically and quickly: from pyrosequencing [705] to sequencing by synthesis [706] and long‐read sequencing [707, 708]. It also involves the transition from amplicons to metagenomics and metatranscriptomics [709], which allows for characterizing “unculturable” species [710, 711] or identifying host–microbiome interactions [712, 713]. Stakeholders should be educated on the limitations and interpretation of results derived from these technologies. In this context, companies offering direct‐to‐consumer microbiome tests present an important challenge given the reproducibility issues of such tests [714]. This highlights the need to establish solid frameworks to regulate their development [715, 716].

Finally, microbial interventions need to address important bottlenecks before their implementation in the clinic. Beyond whole microbial transplants and defined communities, two additional approaches are currently being explored as potential therapies. Postbiotics, inactivated microbial cells or microbial metabolites, have lower potential risks compared to transferring live bacteria to a patient. Microbial metabolites represent a particularly promising direction, as they can be precisely dosed, and mechanisms of action can be studied in animal models prior to human interventions. Among them, SCFAs have been comprehensively studied given their well‐known anti‐inflammatory effects [717, 718]. Tryptophan can also exert beneficial effects by activating immune responses, stimulating gut hormone secretion, and increasing the expression of genes involved in epithelial cell integrity [719]. Numerous studies have also demonstrated the importance of BAs in modulating host immunity and pathogen suppression [720]. Finally, the use of engineered strains synthesizing therapeutic molecules has gained significant attention [721]. Recent attempts at directly editing native gut bacteria inside the host represent an exciting novel approach that overcomes the issue of engraftment by foreign species [722]. However, the use of genetically engineered microbes in mothers and infants will require strict testing to ensure safety.

## CONCLUSION

The maternal–infant microbiome continuum represents a profoundly dynamic and intricately orchestrated period of human development, bridging the coupled ecosystems of preconception through the pivotal windows of pregnancy, birth, and early childhood. Despite its foundational role in dictating lifelong health trajectories, research focusing on intestinal immune disorders and dysbiosis remains disproportionately confined to adult cohorts. Furthermore, the majority of published data concerning the maternal–infant microbiome are heavily concentrated in populations from developed countries. This geographical bias leaves a substantial gap in our understanding of how diverse global environments, diets, and socioeconomic factors shape these critical early‐life trajectories. A defining hallmark of the early‐life micro‐ecosystem is its extreme temporal plasticity and dynamic succession. This implies that the microbiome's influence operates on a strict schedule of critical and sensitive windows. Consequently, capturing the essence of this host–microbe co‐evolution requires a paradigm shift away from cross‐sectional snapshots toward densely sampled, longitudinal birth cohorts.

To decode the complex, high‐dimensional data generated from these highly dynamic interactions, the field urgently needs advanced computational methodologies and AI‐guided multiscale modeling. These algorithmic updates are crucial for extracting key physiological patterns from complex datasets, allowing us to distinguish transient environmental noise from true, disease‐predictive microbial signals.

Fundamentally, this field addresses core ecological questions. The development of the infant gut microbiota is a predictable, nonrandom process of ecological succession. Unraveling the fundamental rules of pioneer colonization, competitive exclusion, and community stabilization in early life provides profound insights into basic microbial ecology. Concurrently, this ecological understanding holds immense translational value. By moving beyond generic definitions of “dysbiosis” to identify stage‐specific functional deficits, we can pave the way for precision microbiome‐targeted diagnostics and therapeutics. From defined microbial consortia to targeted postbiotics and metabolic modulators, these strategies hold the promise of safely correcting developmental trajectories and solving intractable clinical issues ranging from preterm complications and refractory malnutrition to immune and neurodevelopmental disorders.

However, the translation of these discoveries into clinical practice demands extraordinary prudence. Patient safety is a fundamental precept in healthcare, carrying particular weight given the inherent vulnerability of pregnant mothers, neonates, and developing infants. The regulatory landscape must rigorously balance the urgent need for novel therapies with stringent safety assessments, carefully evaluating risks such as bacteremia from live biotherapeutics or the complex challenges of whole‐community transplants.

Ultimately, viewing the early‐life microbiome not merely as a collection of transient passengers, but as a plastic, developing organ, offers a transformative opportunity. By safeguarding the delicate temporal coupling between host ontogeny and microbial ecological succession, we can shift the medical paradigm from managing chronic diseases in adulthood to engineering resilient physiological health from its very biological origin.

## AUTHOR CONTRIBUTIONS

**Huidi Wang**: Conceptualization; writing—original draft; writing—review and editing. **Felicia Tin**: Conceptualization; writing—original draft. **Hui Chen**: Conceptualization; writing—original draft. **Fuxin Jiao**: Conceptualization; writing—original draft. **Min Wu**: Conceptualization; writing—original draft. **Shanshan Sun**: Conceptualization; writing—original draft. **Lisha Lin**: Conceptualization; writing—original draft. **Die Li**: Conceptualization; writing—original draft. **Huimin Zheng**: Conceptualization; writing—original draft. **Zhenxin Niu**: Conceptualization; writing—original draft. **Mengyao Lan**: Conceptualization; writing—original draft. **Bahtiyar Yilmaz**: Writing—review and editing. **Johan G. Eriksson**: Writing—review and editing. **Mengjia Wang**: Conceptualization; writing—original draft. **Andrew Macpherson**: Writing—review and editing. **Jose C. Clemente**: Conceptualization; writing—original draft; writing—review and editing. **Jia Xu**: Conceptualization; writing—original draft; writing—review and editing. **Ri‐hua Xie**: Conceptualization; writing—original draft; writing—review and editing. **Xiaojiao Zheng**: Conceptualization; writing—original draft; writing—review and editing. **Tao Zhou**: Conceptualization; writing—original draft; writing—review and editing. **Jinfeng Wang**: Conceptualization; writing—original draft; writing—review and editing. **Wei Shen**: Conceptualization; writing—original draft; writing—review and editing. **Bing Huang**: Conceptualization; writing—original draft; writing—review and editing. **Xia Chen**: Conceptualization; writing—original draft; writing—review and editing. **Hai Li**: Conceptualization; writing—original draft; writing—review and editing. **Yan He**: Conceptualization; writing—original draft; writing—review and editing.

## CONFLICT OF INTEREST STATEMENT

The authors declare no conflicts of interest.

## ETHICS STATEMENT

No animals or humans were involved in this study.

## Data Availability

Data sharing is not applicable to this article as no datasets were generated or analyzed during the current study. Supplementary materials (graphical abstract, slides, videos, Chinese translated version, and updated materials) may be found in the online DOI or iMeta Science http://www.imeta.science/.
